# Perturbing LSD1 and WNT rewires transcription to synergistically induce AML differentiation

**DOI:** 10.1038/s41586-025-08915-1

**Published:** 2025-04-16

**Authors:** Amir Hosseini, Abhinav Dhall, Nemo Ikonen, Natalia Sikora, Sylvain Nguyen, Yuqi Shen, Maria Luisa Jurgensen Amaral, Alan Jiao, Felice Wallner, Philipp Sergeev, Yuhua Lim, Yuanqin Yang, Binje Vick, Kimihito Cojin Kawabata, Ari Melnick, Paresh Vyas, Bing Ren, Irmela Jeremias, Bethan Psaila, Caroline A. Heckman, M. Andrés Blanco, Yang Shi

**Affiliations:** 1 Ludwig Institute for Cancer Research, Nuffield Department of Medicine, University of Oxford, Oxford, UK; 2 Division of Newborn Medicine, Department of Medicine, Boston Children’s Hospital, Harvard Medical School, Boston, MA USA; 3 Institute for Molecular Medicine Finland (FIMM), Helsinki Institute of Life Science (HiLIFE), iCAN Digital Precision Cancer Medicine Flagship, University of Helsinki, Helsinki, Finland; 4 Medical Research Council Weatherall Institute of Molecular Medicine (MRC WIMM), University of Oxford, John Radcliffe Hospital, Headington, Oxford, UK; 5 Cell and Molecular Medicine, University of California San Diego, La Jolla, CA USA; 6 Research Unit Apoptosis in Hematopoietic Stem Cells, Helmholtz Munich, German Research Center for Environmental Health, Munich, Germany; 7 German Cancer Consortium (DKTK), partner site Munich, a partnership between DKFZ and University Hospital LMU Munich, Munich, Germany; 8 Department of Medicine, Division of Hematology and Medical Oncology, Weill Cornell Medicine, New York, NY USA; 9 Department of Haematology, Oxford University Hospitals NHS Foundation Trust, Oxford, UK; 10 Department of Pediatrics, Dr. von Hauner Children’s Hospital, University Hospital, LMU Munich, Munich, Germany; 11 Department of Biomedical Sciences, School of Veterinary Medicine, University of Pennsylvania, Philadelphia, PA USA

**Keywords:** Targeted therapies, Acute myeloid leukaemia

## Abstract

Impaired differentiation is a hallmark of myeloid malignancies^1,2^. Therapies that enable cells to circumvent the differentiation block, such as all-*trans* retinoic acid (ATRA) and arsenic trioxide (ATO), are by and large curative in acute promyelocytic leukaemia^3^, but whether ‘differentiation therapy’ is a generalizable therapeutic approach for acute myeloid leukaemia (AML) and beyond remains incompletely understood. Here we demonstrate that simultaneous inhibition of the histone demethylase LSD1 (LSD1i) and the WNT pathway antagonist GSK3 kinase^4^ (GSK3i) robustly promotes therapeutic differentiation of established AML cell lines and primary human AML cells, as well as reducing tumour burden and significantly extending survival in a patient-derived xenograft mouse model. Mechanistically, this combination promotes differentiation by activating genes in the type I interferon pathway via inducing expression of transcription factors such as IRF7 (LSD1i) and the co-activator β-catenin (GSK3i), and their selective co-occupancy at targets such as STAT1, which is necessary for combination-induced differentiation. Combination treatment also suppresses the canonical, pro-oncogenic WNT pathway and cell cycle genes. Analysis of datasets from patients with AML suggests a correlation between the combination-induced transcription signature and better prognosis, highlighting clinical potential of this strategy. Collectively, this combination strategy rewires transcriptional programs to suppress stemness and to promote differentiation, which may have important therapeutic implications for AML and WNT-driven cancers beyond AML.

## Abstract (web summary)

Simultaneous inhibition of LSD1 and GSK3 kinase promotes cell differentiation, providing a therapeutic strategy for treating acute myeloid leukaemia.

## Main

AML is a devastating disease with approximately 44,000 new cases diagnosed each year in the USA and EU, and with a 5-year survival rate varying considerably with age of the patients and genetic characteristics of the disease^1^. The standard of care includes intensive combination chemotherapy, which can be consolidated with allogeneic stem and immune cell transplant. For patients ineligible for this option, targeted inhibitors such as hypomethylating agents combined with the BCL-2 inhibitor venetoclax^2^ are used for specific genetic subgroups. Nevertheless, the median survival is still only 8.5 months^2,5–7^. Accordingly, there is an outstanding need for novel AML treatments.

Although genetically heterogenous, AMLs are universally characterized by a prominent differentiation block that disrupts normal myeloid maturation and promotes leukaemia cell self-renewal^2,3^. Although differentiation arrest is a manifestation of the clinical phenotype, it also represents an AML vulnerability that can be leveraged for therapeutic purposes. Unlike most chemotherapy, which eliminates blasts via cytotoxicity, differentiation therapy aims to derepress terminal myeloid maturation programs that reduce the competitive clonal advantage of leukaemia cells^3^. A notable example is acute promyelocytic leukaemia (APL), an AML subtype resistant to standard cytotoxic therapies^3^. In APL, ATRA induces leukaemia cell differentiation^8^ and leads to temporary remission^9^. However, when combined with ATO, the treatment is often curative in 95% of cases by degrading the PML–RARα fusion protein and eliminating leukaemia stem cells (LSCs)^10–12^. This highlights the importance of pursuing combination therapies that promote terminal maturation while inhibiting self-renewal^3,13^.

Developing differentiation therapy for non-APL AMLs to approach the level of success of APL treatment remains a major goal. In this regard, inhibition of chromatin regulators represents an emerging, promising approach to induce the maturation of AML cells. Inhibitors of menin and DOT1L can induce varying degrees of differentiation in MLL-rearranged AML, and IDH1/2 inhibitors also induce differentiation and are highly effective in *IDH1/2*-mutant AML^14–16^. In addition, histone demethylases are potential targets for AML therapy due to their role in AML development and progression^17^. In particular, the histone H3K4me1/2 demethylase LSD1 (ref. ^18^), whose expression is elevated in AML, is crucial for LSC maintenance and proliferation^19^, and inhibition of LSD1 has been shown to induce AML differentiation^19,20^. Consequently, inhibitors of LSD1 are being actively investigated in clinical trials for haematological malignancies, including AML^20^. However, therapeutic efficacy with LSD1 inhibitors alone is limited due to associated toxicities (NCT02177812)^21,22^.

**Fig. 1:**
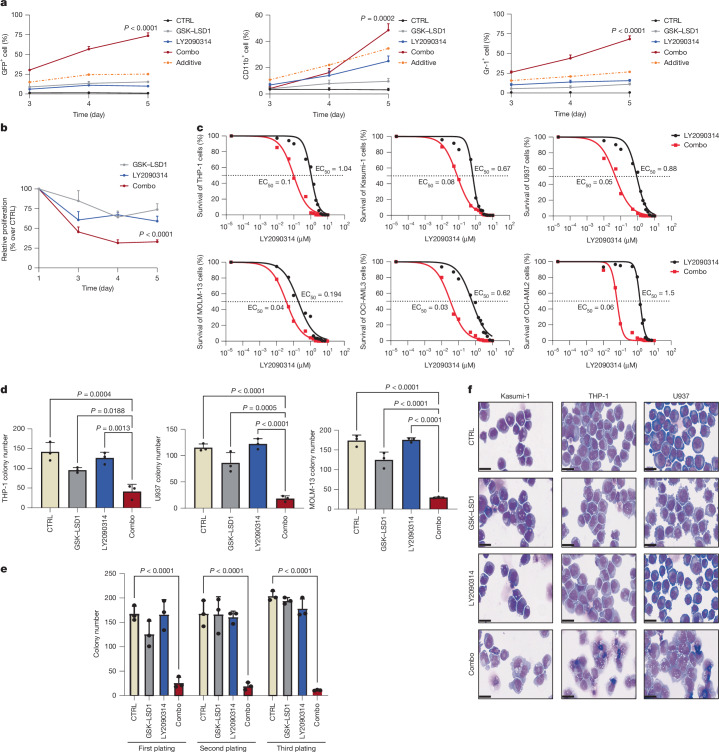
Combination of GSK–LSD1 and LY2090314 inhibits proliferation and impairs clonogenic activity of AML cell lines by inducing differentiation. **a**, Time course measurement of monocyte differentiation markers in the ER-HOXA9 cell line treated with vehicle, GSK–LSD1 (50 nM), LY2090314 (100 nM) and a combination of both inhibitors for 5 days. Data are presented as mean ± s.d. from three biological independent experiments. *P* values were determined using two-way analysis of variance (ANOVA). CTRL, control. **b**, Time course measurement of ER-HOXA9 cell proliferation as in panel **a**. Data are presented as mean ± s.d. from three biological independent experiments. *P* values were determined using two-way ANOVA. **c**, Survival of human AML cell lines treated with different concentrations of LY2090314 (black) and a combination of LY2090314 with 50 nM GSK–LSD1 (red) for 3 days. The luminescence signal was normalized, and dose–response curves and EC_50_ values were calculated using a non-linear regression curve fit. **d**, Quantification of colonies formed by the indicated human cell lines treated with DMSO, GSK–LSD1 (50 nM), LY2090314 (100 nM) and a combination of both inhibitors. Data are presented as mean ± s.d. from three biological independent experiments. *P* values were determined using two-way ANOVA. **e**, Analysis of the clonogenic activity of THP-1 cells by a serial replating assay. Data are presented as mean ± s.d. from three biological independent experiments. *P* values were determined using two-way ANOVA. **f**, Representative images of Kasumi-1, THP-1 and U937 cells treated with the indicated inhibitors for 5 days and stained with Wright–Giemsa. Scale bars, 25 μm. The experiment was repeated three times with similar results.

**Extended Data Fig. 1:**
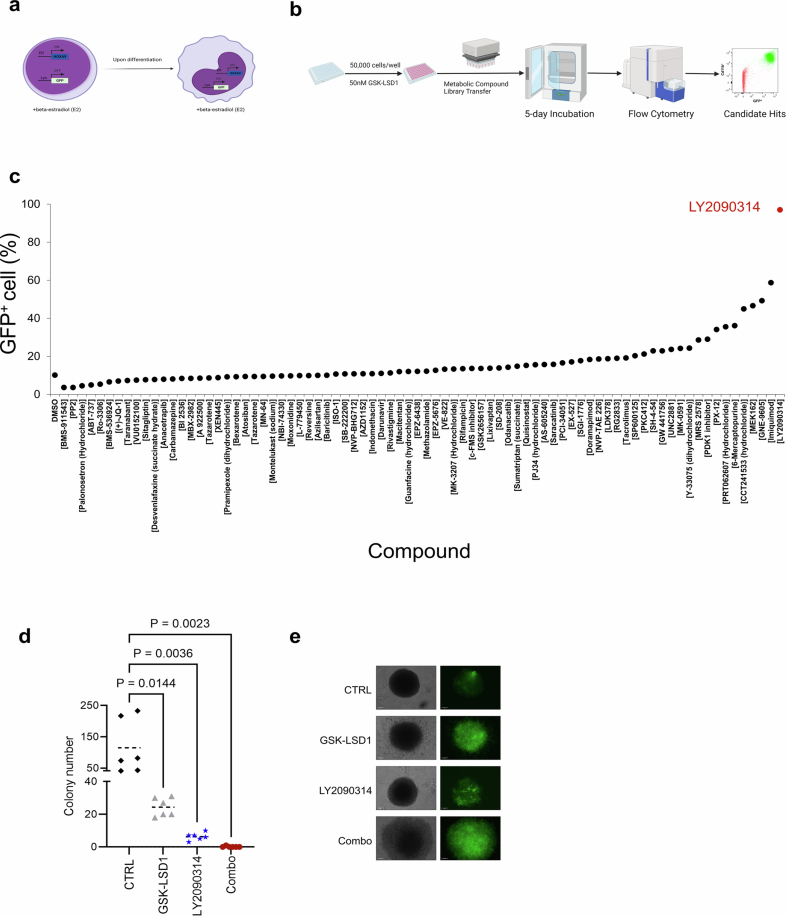
High-throughput screening identifies LY2090314 as an enhancer of LSD1i-mediated differentiation. **a**, ER-HoxA9 as a cellular model for a phenotypic screen of AML differentiation. upon differentiation, ER-HoxA9 cells upregulate GFP fluorescence. The illustrations of the cells were created using BioRender (https://biorender.com). **b**, The small molecule library was pin-transferred into 96-well plates containing ER-HoxA9 cells in media with 50 nM GSK-LSD1. Plates were incubated for 5 days, and differentiation was evaluated by GFP expression and cell-surface marker CD11b. The schematic was created using BioRender (https://biorender.com). **c**, The GSK3 inhibitor, LY2090314, showed the strongest synergy with 50 nM GSK-LSD1 to induce differentiation in ER-HoxA9 cells over 5 days of in vitro culture. **d**, Quantification of colonies formed by ER-HoxA9 cells treated with indicated inhibitors. Data are ± SD of six biological independent experiments. *P*-values were determined using two-way analysis of variance (ANOVA). **e**, Morphology of ER-HoxA9 colonies treated with indicated inhibitors. Representative micrographs from (**d**). Scale bars, 50 μm. The experiment was repeated three times with similar results.

To enhance the efficacy and to mitigate the toxicity issue of LSD1 inhibitors, we screened small molecules for synergistic activity with the LSD1 inhibitor GSK–LSD1 in inducing AML cell differentiation^23^ (Extended Data Fig. 1a,b). Using a collection of bioactive molecules, we performed the screen in ER-HOXA9 cells, a mouse bone marrow model in which ER-HOXA9 blocks myeloid differentiation and features a lysozyme–GFP reporter to assess differentiation^24^ (Extended Data Fig. 1a). Among the compounds screened, the GSK3 inhibitor LY2090314 induced maturation with the most synergy in combination with a low dose of GSK–LSD1 (Extended Data Fig. 1c). Validation experiments confirmed that 50 nM GSK–LSD1 combined with LY2090314 robustly enhanced the levels of lysozyme–GFP and of the differentiation-associated markers CD11b and Gr-1 over 5 days (Fig. 1a).

GSK3 mediates degradation of the transcriptional co-activator β-catenin (encoded by *CTNNB1*)^4^. Upon GSK3i, stabilized β-catenin translocates into the nucleus and typically complexes with the transcription factors TCF and LEF to activate WNT pathway targets^25^. The WNT pathway is associated with self-renewal and oncogenesis^25^. As GSK3 negatively regulates the WNT pathway, it has not been traditionally thought of as an oncogene. However, in some AML subtypes, GSK3 has been shown to have oncogenic functionality by positively regulating the cell cycle^26^ and the HOXA9–MEIS1 transcriptional program^27^. Its inhibition induces cell-cycle arrest and differentiation and has thus been studied as a potential cancer therapeutic target^26–28^. Unlike GSK–LSD1, LY2090314 was well tolerated by patients with AML and had robust on-target activity (more than 450% increase in β-catenin levels)^29^. However, single treatment with LY2090314 showed less than desirable clinical efficacy^29^.

To further test the efficacy of the GSK–LSD1 and LY2090314 drug combination (hereafter referred to as ‘combo’) in differentiation induction, we used orthogonal, functional readouts of myeloid maturation and found that combo treatment synergistically arrested proliferation, eliminated self-renewal as determined by colony formation assays and markedly induced a monocytic differentiation GFP reporter (Fig. 1b and Extended Data Fig. 1d,e). Of note, combo-treated colonies that did form displayed a distinct, diffuse architecture, indicating maturation (Extended Data Fig. 1e). These findings suggest that the combo induces the functional and physiological myeloid differentiation program in ER-HOXA9 cells.

**Extended Data Fig. 2:**
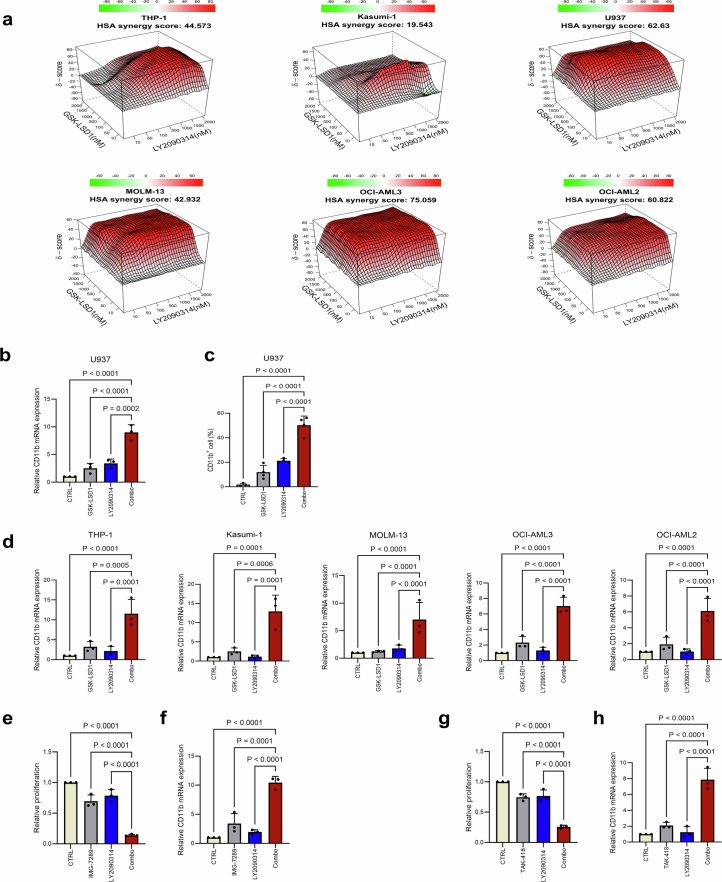
LSD1 and GSK3 inhibitors potently synergize in different AML cell lines. **a**. Synergy plot using an HSA model for THP-1, Kasumi-1, U937, MOLM-13, OCI-AML3 and OCI-AML2 cells treated with different concentrations of GSK-LSD1 and LY2090314 for 5 days. **b**, Analysis of *CD11b* mRNA relative levels in U937 cells treated with the indicated inhibitors for 5 days. Values were normalized against *GAPDH*. Data are presented as mean ± SD of 3 biological independent experiments (n = 3). *P*-values were determined using two-way analysis of variance (ANOVA). **c**, Analysis of CD11b cell-surface protein relative levels in U937 cells treated with the indicated inhibitors for 5 days. Data are presented as mean ± SD of 3 biological independent experiments (n = 3). *P*-values were determined using two-way analysis of variance (ANOVA). **d**, Analysis of *CD11b* mRNA relative levels in human AML cells treated with the indicated inhibitors for 5 days. Values were normalized against *GAPDH*. Data are presented as mean ± SD of 3 biological independent experiments (n = 3). *P*-values were determined using two-way analysis of variance (ANOVA). **e**, Analysis of THP-1 cell viability after treatment with IMG-7289, LY2090314 or combination of both inhibitors for 5 days by Cell-Titer-Glo assay. Data are presented as mean ± SD of 3 biological independent experiments (n = 3). *P*-values were determined using two-way analysis of variance (ANOVA). **f**, Analysis of *CD11b* mRNA relative levels in THP-1 cells treated with IMG-7289, LY2090314 or combination of both inhibitors for 5 days. Values were normalized against *GAPDH*. Data are presented as mean ± SD of 3 biological independent experiments (n = 3). *P*-values were determined using two-way analysis of variance (ANOVA). **g**, Analysis of THP-1 cell viability after treatment with TAK-418, LY2090314 or combination of both inhibitors for 5 days by Cell-Titer-Glo assay. Data are presented as mean ± SD of 3 biological independent experiments (n = 3). *P*-values were determined using two-way analysis of variance (ANOVA). **h**, Analysis of *CD11b* mRNA relative levels in THP-1 cells treated with TAK-418 or LY2090314 and combination of both inhibitors for 5 days. Values were normalized against *GAPDH*. Data are presented as mean ± SD of 3 biological independent experiments (n = 3). *P*-values were determined using two-way analysis of variance (ANOVA).

To assess whether human AML cell lines can undergo differentiation by the combo treatment, we examined a mutationally diverse panel of six cell lines. In all cases, the combo treatment synergistically reduced the half-maximal effective concentration (EC_50_) of the LY2090314 dose–response curve, demonstrated strong synergy (Fig. 1c and Extended Data Fig. 2a) and robustly reduced colony formation ability (Fig. 1d). To investigate self-renewal further, we performed serial colony formation assays with drug washouts. Cells isolated from the first plating of drug-treated colonies were harvested, washed and seeded serially for two additional rounds of plating without drug treatment. If self-renewal is lost after the first seeding, its depletion should remain without continued drug treatment. Indeed, after drug washout, colony formation ability was continually and progressively exhausted over the second and third plating in combo-treated cells, but not in control or single-drug-treated cells (Fig. 1e). Finally, the combo treatment synergistically induced high levels of CD11b in all cell lines and produced visibly mature cells (Fig. 1f and Extended Data Fig. 2b–d).

**Fig. 2:**
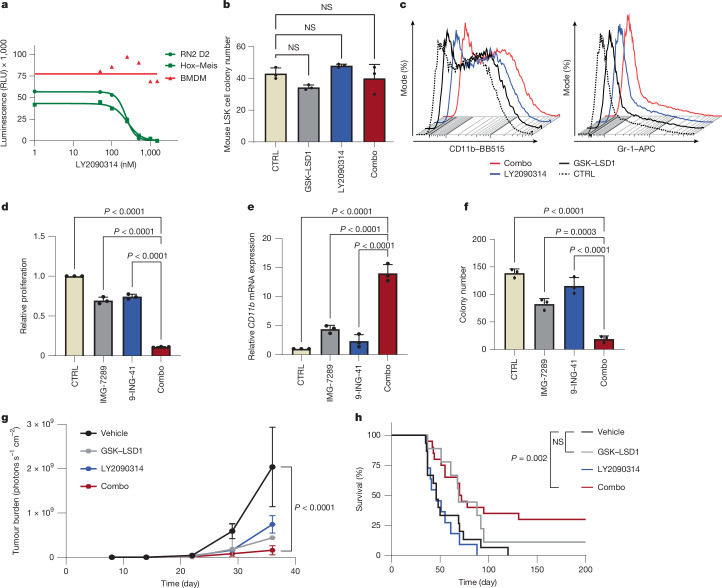
Combo treatment specifically inhibits proliferation of leukaemia cells in vitro and in vivo*.* **a**, Mouse AML cells treated with 50 nM GSK–LSD1 and different concentrations of LY2090314 for 5 days; cell growth was determined by the Cell-Titer-Glo assay. Data are presented as mean ± s.d. from two biological independent experiments. BMDM, bone marrow-derived macrophage; RLU, relative light unit. **b**, Quantification of colonies formed by normal mouse LSK cells treated with the indicated inhibitors. Data are presented as mean ± s.d. from three biological independent experiments. *P* values were determined using two-way ANOVA. NS, not significant. **c**, Flow-cytometric quantification of CD11b and Gr-1 expression in the cells harvested from methylcellulose in panel **b**. **d**, Analysis of cell viability in THP-1 cells treated with the indicated inhibitors for 5 days by the Cell-Titer-Glo assay. Data are presented as mean ± s.d. from three biological independent experiments. *P* values were determined using two-way ANOVA. **e**, Analysis of *CD11b* mRNA relative level in THP-1 cells treated with the indicated inhibitors for 5 days. Values were normalized against *GAPDH*. Data are presented as mean ± s.d. from three biological independent experiments. *P* values were determined using two-way ANOVA. **f**, Quantification of colonies formed by THP-1 cells treated with the indicated inhibitors. Data are presented as mean ± s.d. from three biological independent experiments. *P* values were determined using two-way ANOVA. **g**,**h**, Tumour burden (**g**) and Kaplan–Meier survival curves (**h**) of mice treated with vehicle (*n* = 15), GSK–LSD1 (*n* = 9), LY2090314 (*n* = 11) or a combination of both inhibitors (*n* = 20) in a syngeneic model of a HOXA9–MEIS1-driven AML model. *P* values were determined using the log-rank test. The error bars represent mean ± s.e.m. (**g**). *P* values were determined using two-way ANOVA. Source data

To investigate the selectivity of the combo treatment for leukaemia cells, we performed dose–response proliferation assays in mouse leukaemia cells and normal bone marrow-derived macrophages. Although RN2 (MLL-AF9/*Nras*^*G12V*^) and HOXA9–MEIS1-overexpressing AML cells showed high sensitivity to the combo treatment, bone marrow-derived macrophage proliferation was unaffected even at the highest doses (Fig. 2a). Similarly, the combo treatment had no significant effect on the clonogenic activity and differentiation of normal mouse haematopoietic stem and progenitor-enriched Lin^−^Sca^+^Kit^+^ (LSK) populations (Fig. 2b,c). This suggests that the drug combo treatment has selectivity for leukaemic blasts and may be well tolerated in vivo.

As there are multiple inhibitors of LSD1 and GSK3, we next sought to confirm that the efficacy of combo treatment was not unique to GSK–LSD1 and LY2090314. We first tested bomedemstat (IMG-7289), an irreversible LSD1 inhibitor in clinical trials for myeloid malignancies^30^, and TAK-418 (ref. ^31^). Like GSK–LSD1, bomedemstat or TAK-418 synergistically reduced proliferation and induced CD11b in combination with LY2090314 in THP-1 cells (Extended Data Fig. 2e–h). Although GSK–LSD1 has been shown to function mainly by disrupting the important LSD1–GFI1 interaction^32–34^, TAK-418 mainly inhibits LSD1 demethylase activity with minimal disruption of the LSD1–GFI1 interaction^31^. Thus, these data suggest that disrupting the LSD1–GFI1 interaction or inhibiting LSD1 catalytic activity can both synergize with GSK3i to induce AML cell differentiation. We next tested the efficacy of another GSK3 inhibitor, 9-ING-41, which is also currently in clinical trials (NCT03678883)^35^. As with LY2090314, a low dose of 9-ING-41 synergized with IMG-7289 to halt proliferation, clonogenic activity and markedly induce CD11b (Fig. 2d–f).

We next performed in vivo studies. We used a syngeneic HOXA9–MEIS1 retroviral overexpression transplant model that recapitulates much of MLL-AF9 AML biology while retaining applicability to non-MLL-rearranged AML types with prominent HOXA9 and MEIS1 activity^24^. Although GSK–LSD1 and LY2093014 alone had a modest effect on disease progression and survival, the combo treatment provided the greatest reduction of disease progression (Fig. 2g) and yielded a significant lifespan extension (Fig. 2h).

**Fig. 3:**
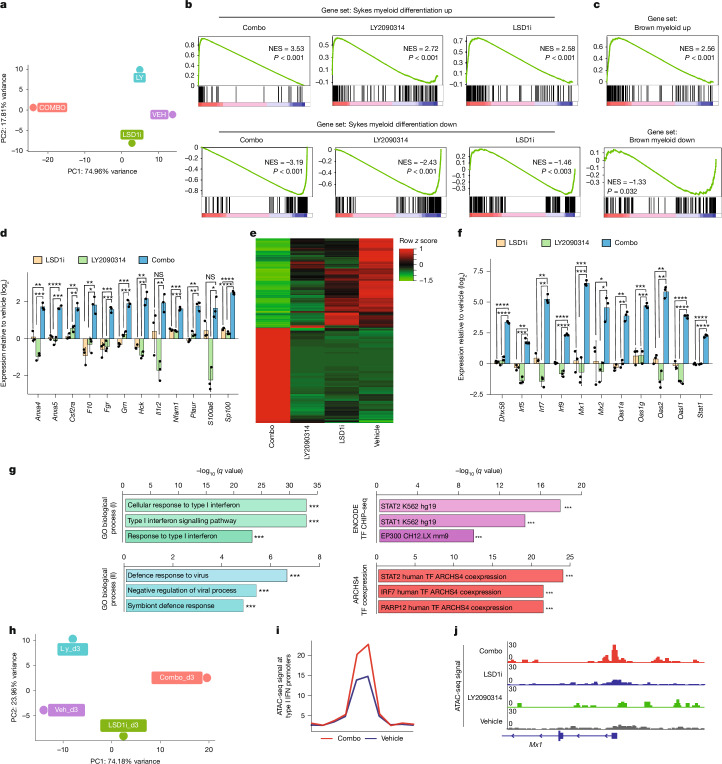
Combo treatment triggers alterations in the transcriptional and chromatin accessibility profiles associated with myeloid differentiation and IFN response in ER-HOXA9 cells. **a**, PCA of RNA sequencing (RNA-seq) of ER-HOXA9 cells treated with vehicle (VEH), GSK–LSD1, LY2090314 (LY) and a combination of both inhibitors (COMBO). **b**, GSEA of myeloid maturation signatures in drug-treated ER-HOXA9 cells. NES, normalized enrichment score. **c**, GSEA of additional myeloid maturation signatures in ER-HOXA9 cells treated with combo versus vehicle. **d**, Expression of genes relating to myeloid differentiation in drug-treated ER-HOXA9 cells. Data are presented as mean ± s.d. from three independent biological replicates. *P* values indicate the significance of unpaired, two-tailed Student’s *t*-tests. See source data for individual *P* values. **e**, Heatmap of expression of genes synergistically upregulated or downregulated upon combo treatment. **f**, Expression of genes relating to the type I IFN response in drug-treated ER-HOXA9 cells. Data are presented as mean ± s.d. from three independent biological replicates. See source data for individual *P* values. **g**, EnrichR database pathways enriched in upregulated combo synergy signature genes. Significance of enrichment *z* scores is shown as *q* values (corresponding to *P* values adjusted for significance by the Benjamini–Hochberg method). ChIP–seq, chromatin immunoprecipitation followed by sequencing; GO, Gene Ontology; TF, transcription factor. **h**, PCA of ATAC-seq in drug-treated ER-HOXA9 cells. **i**, ATAC-seq signal at promoters of type I IFN response genes in combo-treated and vehicle-treated ER-HOXA9 cells. **j**, Tracks showing ATAC-seq signal at the *Mx1* promoter in drug-treated cells. The asterisks indicate significance: *P < 0.05, **P < 0.01, ***P < 0.001, ****P < 0.0001 and NS (*P* > 0.05). Source data

**Extended Data Fig. 3:**
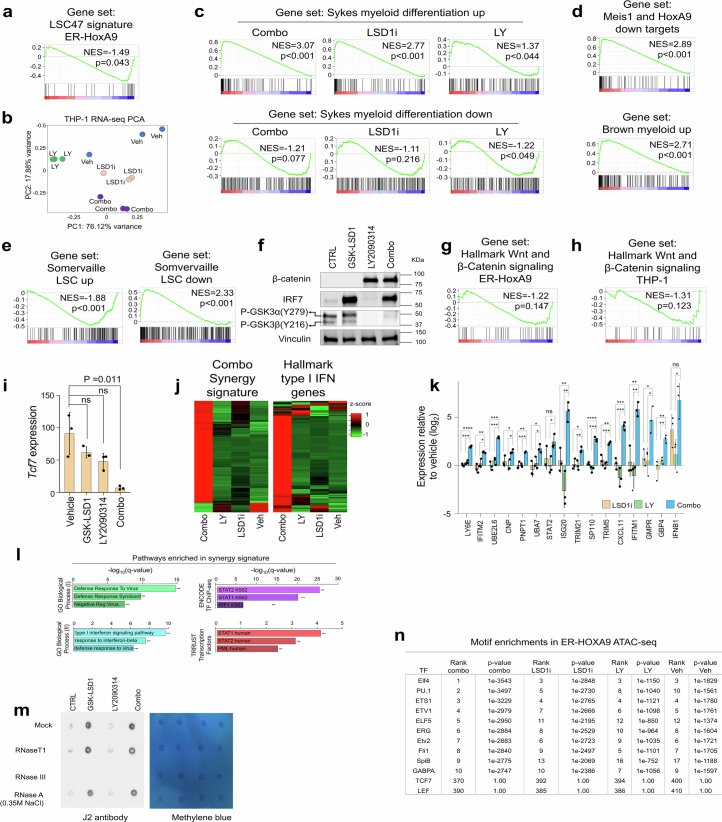
Combo treatment triggers transcriptional changes associated with myeloid differentiation and interferon response in AML cells. **a**, GSEA of 47-gene leukemia stem cell (LSC) signature in ER-HoxA9 cells treated with drug combo vs. vehicle. **b**, PCA of RNA-seq of THP-1 cells treated with vehicle, GSK-LSD1, LY2090314 or combination of both inhibitors. **c**, GSEA of myeloid maturation signatures in drug-treated THP-1 cells. **d**, GSEA of additional myeloid maturation signatures in THP-1 cells treated with combo vs. vehicle. **e**, GSEA of Somervaille leukemia stem cell (LSC) signatures in THP-1 cells treated with combo vs. vehicle. **f**, Western immunoblots for β-catenin, phospho-GSK3α/β ^(Y279/Y216)^, IRF7 and vinculin after treatment with the indicated inhibitors for 3 days in THP-1 cells. Vinculin was used as a loading control. The experiment was repeated three times with similar results. **g**, GSEA of Wnt and β-catenin signaling genes in ER-HoxA9 cells treated with drug combo vs. vehicle. **h**, GSEA of Wnt and β-catenin signaling genes in THP-1 cells treated with drug combo vs. vehicle. **i**, Expression of *TCF7* (TCF1) in drug-treated ER-HoxA9 cells. Data are presented as mean values ± SD from three independent biological replicates. *P*-values indicate the significance of unpaired, two-tailed Student *t*-tests. “ns” indicates not significant. **j**, Heatmap of expression of genes synergistically upregulated or downregulated upon combo treatment (left), and type I interferon signature genes (right). **k**, Expression of genes relating to the type I interferon response in drug-treated THP-1 cells. Data are presented as mean values ± SD from three independent biological replicates. *P*-values indicate the significance of unpaired, two-tailed Student *t*-tests. See source data for individual *P*-values. Asterisks indicate significance at these levels: *P < 0.05, **P < 0.01, ***P < 0.001, ****P < 0.0001, “ns” indicates not significant. **l**, EnrichR database pathways enriched in upregulated combo synergy signature genes. Significance of enrichment z-scores is shown as *q*-values (corresponding to *p*-values adjusted for significance by Benjamini-Hochberg method). **m**, Dot blot for dsRNA using total RNA from THP-1 cells treated with indicated inhibitors for 3 days. Total RNA extract treated with mock, RNase T1, RNase III, or RNase A (350 mM NaCl) was dotted on Hybond N+ membranes, visualized by methylene blue staining and immunoblotted with J2 antibody. **n**, Table shows rank and *P*-value of enrichment of top transcription factor (TF) motifs in accessible chromatin of ER-HoxA9 cells treated with vehicle, GSK-LSD1, LY2090314 or combination of both inhibitors. Motifs shown in all treatments are the top 10 motifs from combo-treated cells. Enrichment rank and *P*-values of *TCF7* (TCF1) and LEF motifs are also shown. Motif enrichment significance was determined via hypergeometric tests. Source data

Next, we wanted to explore what the molecular mechanism underlying the synergistic effect of the combo treatment to induce AML cell differentiation is. To address this, we first investigated the effects of drug treatment on the transcriptome and epigenome in ER-HOXA9 cells. As expected, after 5 days of treatment, more genes were differentially expressed in response to the combo treatment (*n* = 2,201) than to each drug alone (*n* = 772 for GSK–LSD1 and *n* = 1,224 for LY2093014; Supplementary Table 1). Principle component analysis (PCA) suggests that the combo treatment induced a chromatin state that is distant and distinct from the disparate states induced by single-agent treatment (Fig. 3a). Gene set enrichment analysis (GSEA) and clustering confirmed that the combo treatment synergistically upregulated myeloid differentiation expression signatures and downregulated LSC signatures (Fig. 3b–d and Extended Data Fig. 3a). These findings were also found in THP-1 cells (Extended Data Fig. 3b–e and Supplementary Table 2). Although LY2093014 demonstrated its on-target inhibitory effect, evidenced by reduced GSK3α/β autophosphorylation on Tyr279/216 and the subsequent elevation of β-catenin levels^36,37^ (Extended Data Fig. 3f), canonical WNT pathway signatures were weakly, if at all, enriched in LY2093014 or combo-treated ER-HOXA9 and THP-1 cells (Extended Data Fig. 3g,h, and data not shown). In addition, TCF1, a key transcription factor that interacts with β-catenin to activate canonical WNT pathway target genes, was downregulated upon combo treatment (Extended Data Fig. 3i). Collectively, these results provide compelling evidence that the combo treatment induces the differentiation program and impairs LSC activity, potentially by suppressing the WNT pathway.

We next focused on genes upregulated upon combo treatment. Enrichment analyses and manual inspection revealed a marked overrepresentation of genes in the type I interferon (IFN) signalling pathway, including *Stat1*, *Irf9*, *Irf7* and a panel of IFN-stimulated genes (ISGs) such as *Mx1*, *Ddx58* and *Oasl1*, which we termed the ‘synergy signature’ (Fig. 3e–g and Supplementary Table 3). These results were also found in THP-1 cells (Extended Data Fig. 3j–l). This suggests the possibility that activation of genes in the type I IFN pathway could be promoting combo-induced maturation, as IRF-family transcription factors can upregulate genes mediating the antimicrobial response component of functional granulocytic differentiation^38^. We have recently demonstrated that LSD1i induces double-stranded RNA (dsRNA) and the IFN pathway in melanoma, which stimulates antitumour immunity^39^. Similarly, we also observed elevated levels of dsRNA and *IFNB1* expression in AML cells treated with GSK–LSD1 alone or in combination with LY2090314 (Extended Data Fig. 3k,m). This suggests a conserved role of LSD1 in suppressing dsRNA and the IFN pathway across haematopoietic and select solid tumours and provides evidence of on-target effects of GSK–LSD1.

To determine the effect of the combo on the chromatin landscape, we performed ATAC-seq. We found that global levels of chromatin accessibility did not appear to change dramatically under any treatments, and PCA again showed that the combo treatment induced large chromatin-state changes distinct from single-agent treatment (Fig. 3h). Although motifs for differentiation-associated transcription factors such as ETS and ETV family factors were for the most part already enriched in open chromatin before drug treatments (Extended Data Fig. 3n and Supplementary Tables 4–6), significant increases in chromatin accessibility were observed at promoters of type I IFN pathway genes in the combo treatment compared with single-agent treatment (Fig. 3i,j). Of note, motifs for the WNT pathway transcription factors TCF and LEF were not strongly enriched in any treatments (Extended Data Fig. 3n). These findings are not unprecedented, as β-catenin can interact with transcription factors other than TCF1–LEF1, such as HIF1α and IRF3 (refs. ^40,41^). For instance, in colorectal cancer cells exhibiting elevated hypoxia levels, the canonical β-catenin–TCF4 signalling pathway is redirected towards a β-catenin–HIF1α signalling pathway^40^. In addition, IRF3 and β-catenin interact and colocalize to the promoter region of IFNβ in response to synthetic dsRNA^41^.

**Fig. 4:**
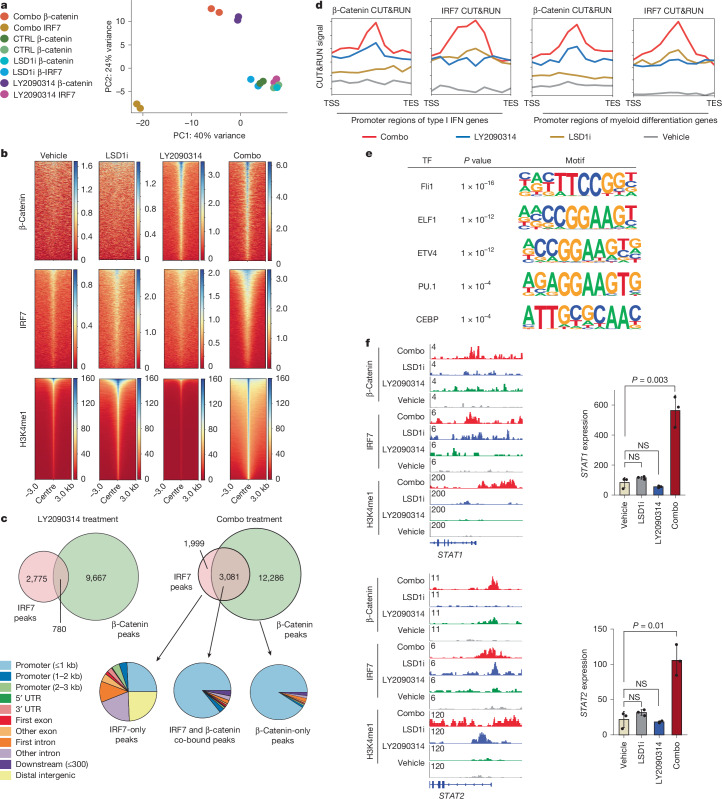
Combo treatment induces selective co-occupancy of IRF7 and β-catenin at the promoter of the type I IFN signalling pathway genes. **a**, PCA of IRF7 and β-catenin CUT&RUN in THP-1 cells treated with vehicle, GSK–LSD1, LY2090314 and a combination of both inhibitors. **b**, Heatmaps showing global signal intensity of IRF7, β-catenin and H3K4me1 CUT&RUN in THP-1 cells treated with vehicle, GSK–LSD1, LY2090314 or a combination of both inhibitors. **c**, Venn diagrams showing overlap between IRF7 and β-catenin binding in LY2090314-treated and combo-treated THP-1 cells (top), and genomic distributions of singly and co-bound peaks shown for combo-treated cells (below). UTR, untranslated region. **d**, CUT&RUN signal of IRF7 and β-catenin at promoters of type I IFN response genes (left two panels) and myeloid differentiation signature genes (right two panels) after drug treatments. TES, transcription end site; TSS, transcription start site. **e**, Enrichment of top motifs relating to myeloid differentiation in genomic regions co-bound by IRF7 and β-catenin. Motif enrichment significance was determined via hypergeometric tests. **f**, Tracks of IRF7, β-catenin and H3K4me1 CUT&RUN at the *STAT1* and *STAT2* loci in drug-treated THP-1 cells. Gene expression of each gene after drug treatments is shown to the right of the corresponding CUT&RUN track. Data are presented as mean ± s.d. from three independent biological replicates. *P* values indicate the significance of unpaired, two-tailed Student’s *t*-tests.

We hypothesized that β-catenin and IRF7 may form a critical regulatory unit that further drives the genes in the IFN pathway to induce differentiation upon combo treatment based on (1) the enrichment of type I IFN pathway regulatory elements in open chromatin, (2) the upregulation of IRF7, and (3) the stabilization of β-catenin in response to LY2093014 treatment. To test this hypothesis, we performed CUT&RUN on β-catenin and IRF7 in THP-1 cells. β-Catenin showed chromatin binding only in LY2093014 and combo-treated cells, as expected. IRF7 peaks, which are almost exclusively localized to promoters, were significantly higher in combo-treated cells than in single-agent-treated cells and were absent in control cells (Fig. 4a–c and Supplementary Tables 7, 8). β-Catenin and IRF7 also had higher binding signals at genes relating to the type I IFN pathway and myeloid differentiation upon combo treatment than upon single-agent treatment, consistent with combo-induced synergistic activation of genes in these pathways (Fig. 4d). In LY2093014-treated cells, IRF7 and β-catenin showed moderate colocalization, with 3,555 IRF7 peaks and 10,447 β-catenin peaks having 780 overlapping peaks (22% of IRF7 peaks and 8% of β-catenin peaks). However, combo treatment led to dramatically increased colocalization of β-catenin and IRF7, with 5,080 IRF7 peaks and 15,367 β-catenin peaks having 3,081 overlapping peaks (61% of IRF7 peaks and 20% of β-catenin peaks; Fig. 4c and Supplementary Table 9). Co-bound peaks localized primarily to promoters and were enriched for motifs of transcription factors that regulate myeloid differentiation such as PU.1, MYB and several ETS family factors (Fig. 4c,e and Supplementary Table 10).

**Extended Data Fig. 4:**
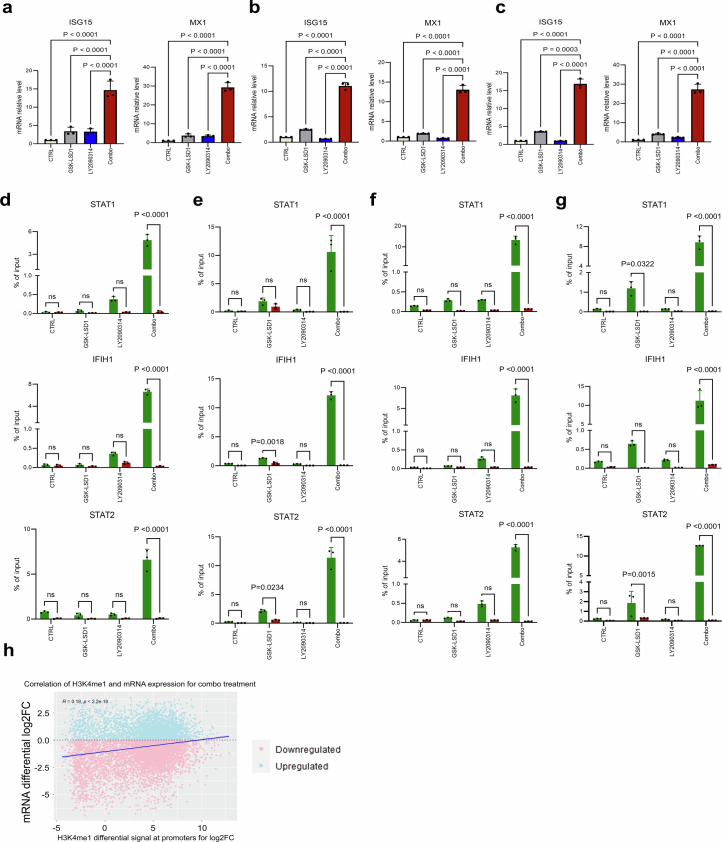
Combo treatment activates type I interferon signaling in AML cells. **a**, **b**, **c**, Analysis of *ISG15* and *MX1* mRNA relative levels in THP-1 (**a**), MOLM-13 (**b**) and U937 (**c**) cells treated with indicated inhibitors. Values were normalized against *GAPDH*. Data are presented as mean ± SD of 3 biological independent experiments (n = 3). *P*-values were determined using two-way analysis of variance (ANOVA). **d**, CUT&RUN-qPCR occupancy analysis of β-catenin on promoter of *STAT1*, *IFIH1* and *STAT2* in OCI-AML3 cell treated with indicated inhibitors. Data are presented as mean ± SD of 3 biological independent experiments (n = 3). *P*-values were determined using two-way analysis of variance (ANOVA). **e**, CUT&RUN-qPCR occupancy analysis of IRF7 on promoter of *STAT1*, *IFIH1* and *STAT2* in OCI-AML3 cell treated with indicated inhibitors. Data are presented as mean ± SD of 3 biological independent experiments (n = 3). *P*-values were determined using two-way analysis of variance (ANOVA). **f**, CUT&RUN-qPCR occupancy analysis of β-catenin on promoter of *STAT1*, *IFIH1* and *STAT2* in MOLM-13 cell treated with indicated inhibitors. Data are presented as mean ± SD of 3 biological independent experiments (n = 3). *P*-values were determined using two-way analysis of variance (ANOVA). **g**, CUT&RUN-qPCR occupancy analysis of IRF7 on promoter of *STAT1*, *IFIH1* and *STAT2* in MOLM-13 cell treated with indicated inhibitors. Data are presented as mean ± SD of 3 biological independent experiments (n = 3). *P*-values were determined using two-way analysis of variance (ANOVA). **h**, Scatter correlation plot of H3K4me1 and mRNA expression in THP-1 cells treated with combo. R indicates Pearson correlation value. “ns” indicates not significant.

Among the numerous changes detected in β-catenin and IRF7 localization upon drug treatments, the most notable was their co-occupancy at the promoters of several of the most critical drivers of the IFN response, such as *STAT1* and *STAT2*, upon combo, but not single-agent, treatment (Fig. 4f). This colocalization correlated with transcriptional outputs, as these genes were synergistically upregulated by combo treatment (Fig. 4f and Extended Data Fig. 4a–c). Consistently, in two additional AML cell lines, OCI-AML3 and MOLM-13, we observed strong co-enrichment of both β-catenin and IRF7 on the promoters of key type I IFN genes — *STAT1*, *IFIH1* and *STAT2* — only in cells treated with combo (Extended Data Fig. 4d–g). Consistent with the function of LSD1 as an H3K4me1/2 demethylase^18^, inhibiting LSD1 resulted in a global increase in H3K4me1 levels, with this effect being more pronounced in combo-treated cells (Fig. 4b). This was associated with enhanced chromatin accessibility (Fig. 3h–j) and an increase in gene expression following combo treatment (Extended Data Fig. 4h).

**Extended Data Fig. 5:**
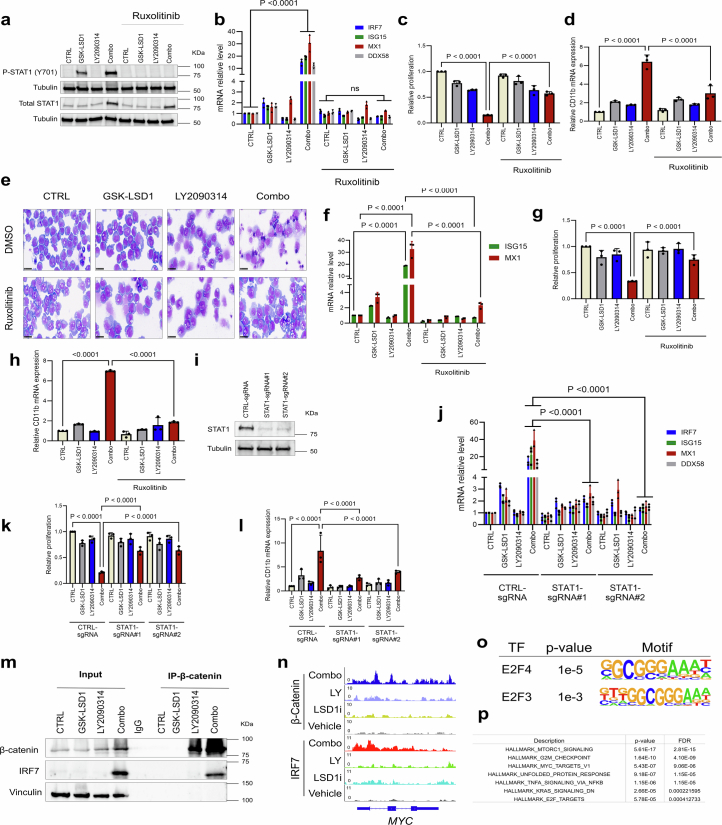
The impact of combo treatment is significantly diminished by the inhibition of STAT1 activation. **a**, Western blot analysis of p-STAT1 (Y701) and total STAT1 from THP-1 cells treated with indicated inhibitors in the presence or absence of ruxolitinib for 5 days. Tubulin was used as a loading control. The experiment was repeated three times with similar results. **b**, Analysis of *IRF7*, *ISG15*, *MX1* and *DDX58* mRNA relative levels in THP-1 cells treated with indicated inhibitors in the presence or absence of ruxolitinib for 5 days. Values were normalized against *GAPDH*. Data are presented as mean ± SD of 3 biological independent experiments (n = 3). *P*-values were determined using two-way analysis of variance (ANOVA). **c**, Analysis of cell viability in THP-1 cells treated with the indicated inhibitors in the presence or absence of ruxolitinib for 5 days by Cell-Titer-Glo assay. Data are presented as mean ± SD of 3 biological independent experiments (n = 3). *P*-values were determined using two-way analysis of variance (ANOVA). **d**, Analysis of *CD11b* mRNA relative levels in THP-1 cells treated with the indicated inhibitors in the presence or absence of ruxolitinib for 5 days. Data are presented as mean ± SD of 3 biological independent experiments (n = 3). *P*-values were determined using two-way analysis of variance (ANOVA). **e**, Representative images of THP-1 cells treated with indicated inhibitors in the presence or absence of ruxolitinib for 5 days and stained with Wright-Giemsa. Scale bars, 25 μm. The experiment was repeated three times with similar results. **f**, Analysis of *ISG15* and *MX1* mRNA relative levels in MOLM-13 cells treated with indicated inhibitors in the presence or absence of ruxolitinib for 5 days. Values were normalized against *GAPDH*. Data are presented as mean ± SD of 3 biological independent experiments (n = 3). *P*-values were determined using two-way analysis of variance (ANOVA). **g**, Analysis of cell viability in MOLM-13 cells treated with the indicated inhibitors in the presence or absence of ruxolitinib for 5 days by Cell-Titer-Glo assay. Data are presented as mean ± SD of 3 biological independent experiments (n = 3). *P*-values were determined using two-way analysis of variance (ANOVA). **h**, Analysis of *CD11b* mRNA relative levels in MOLM-13 cells treated with the indicated inhibitors in the presence or absence of ruxolitinib for 5 days. Values were normalized against *GAPDH*. Data are presented as mean ± SD of 3 biological independent experiments (n = 3). *P*-values were determined using two-way analysis of variance (ANOVA). **i**, Western blot analysis of STAT1 protein level in THP-1 cells. Two independent clones were used for *STAT1*-KO. Tubulin was used as a loading control. The experiment was repeated three times with similar results. **j**, Analysis of *IRF7*, *ISG15*, *MX1* and *DDX58* mRNA relative levels in *STAT1*-KO THP-1 cells treated with indicated inhibitors for 5 days. Values were normalized against *GAPDH*. Data are presented as mean ± SD of 3 biological independent experiments (n = 3). *P*-values were determined using two-way analysis of variance (ANOVA). **k**, Analysis of cells viability in *STAT1*-KO THP-1 cells treated with indicated inhibitors for 5 days by Cell-Titer-Glo assay. Data are presented as mean ± SD of 3 biological independent experiments (n = 3). *P*-values were determined using two-way analysis of variance (ANOVA). **l**, Analysis of *CD11b* mRNA relative levels in *STAT1*-KO THP-1 cells treated with indicated inhibitors for 5 days. Data are presented as mean ± SD of 3 biological independent experiments (n = 3). *P*-values were determined using two-way analysis of variance (ANOVA). **m**, Analysis of β-catenin and IRF7 interactions by co-immunoprecipitation in THP-1 cells treated with indicated inhibitors for 5 days. Vinculin was used as a loading control. The experiment was repeated three times with similar results. **n**, CUT&RUN tracks of IRF7 and β-catenin at the *MYC* locus in drug-treated THP-1 cells. **o**, Enrichment of E2F motifs in genomic regions co-bound by IRF7 and β-catenin in combo-treated THP-1 cells. Motif enrichment significance was determined via hypergeometric tests. **p**, Top 7 ChipEnrich Hallmark database pathway enrichments in genes mapping to genomic regions co-bound by IRF7 and β-catenin in combo-treated THP-1 cells. *P*-values were determined via ChIP-Enrich method.

Activation of STAT1, a critical transcription factor in the IFN pathway, has been linked to monocytic differentiation and the maturation of macrophages^42–44^. This raises the possibility that the combo may promote differentiation by activating key IFN response and differentiation-promoting genes such as *STAT1*. To test this hypothesis, we first confirmed that the combo synergistically activated ISGs such as *ISG15* and *MX1* in all cell lines tested (Extended Data Fig. 4a–c). Combo treatment also synergistically upregulated the expression of *STAT1* transcripts, total STAT1 protein and activated phospho-STAT1 (Fig. 4f and Extended Data Fig. 5a). To determine the importance of STAT1 in mediating combo-induced differentiation, we treated THP-1 cells with single agents or the combo treatment and delivered the JAK1/JAK2 inhibitor ruxolitinib, which reduces the activated phospho-Y701 form of STAT1 (ref. ^45^) (Extended Data Fig. 5a). Ruxolitinib completely abrogated the induction of ISGs, indicating that combo-driven IFN pathway gene activation was dependent on STAT1 activation (Extended Data Fig. 5b). Ruxolitinib treatment also suppressed the combo-induced synergistic upregulation of CD11b, cell differentiation and losses of proliferation (Extended Data Fig. 5c–e). Similar results were also observed in MOLM-13 cells (Extended Data Fig. 5f–h).

To confirm that this was not due to off-target effects of ruxolitinib, we also genetically knocked out *STAT1* by CRISPR and found that *STAT1* knockout phenocopied all aspects of ruxolitinib treatment (Extended Data Fig. 5i–l). Finally, epigenomic co-occupancy data suggest that IRF7 and β-catenin may physically interact to coordinate transcriptional regulation at promoters of key regulators such as STAT1 (Fig. 4f). Consistently, co-immunoprecipitation experiments showed physical interactions between β-catenin and IRF7 only upon combo treatment (Extended Data Fig. 5m). Collectively, these data support our model that the combo treatment not only enhances the expression of type I IFN genes but also triggers the activation of IRF7 and stabilization of β-catenin, with their physical interaction and co-occupancy at promoters, particularly STAT1, leading to the activation of myeloid differentiation and stable and strong activation of the type I IFN pathway.

In addition to β-catenin localization to IFN pathway gene promoters, we also observed β-catenin and IRF7 colocalizing at promoters and occasionally gene bodies of cell-cycle regulators, including the classical β-catenin transcriptional targets such as *MYC* (Extended Data Fig. 5n). Indeed, co-bound β-catenin and IRF7 CUT&RUN peaks were enriched for G2/M checkpoint genes, MYC gene sets and E2F-binding motifs (Extended Data Fig. 5o,p and Supplementary Table 11). Cell-cycle and MYC-related genes with co-bound β-catenin and IRF7 were almost universally downregulated upon combo treatment, which is consistent with the functional effect of the combo treatment on reducing stemness and promoting differentiation, and further suggests that β-catenin and IRF7 could have context-specific transcriptionally repressive activity, contributing to suppressing oncogenesis.

**Extended Data Fig. 6:**
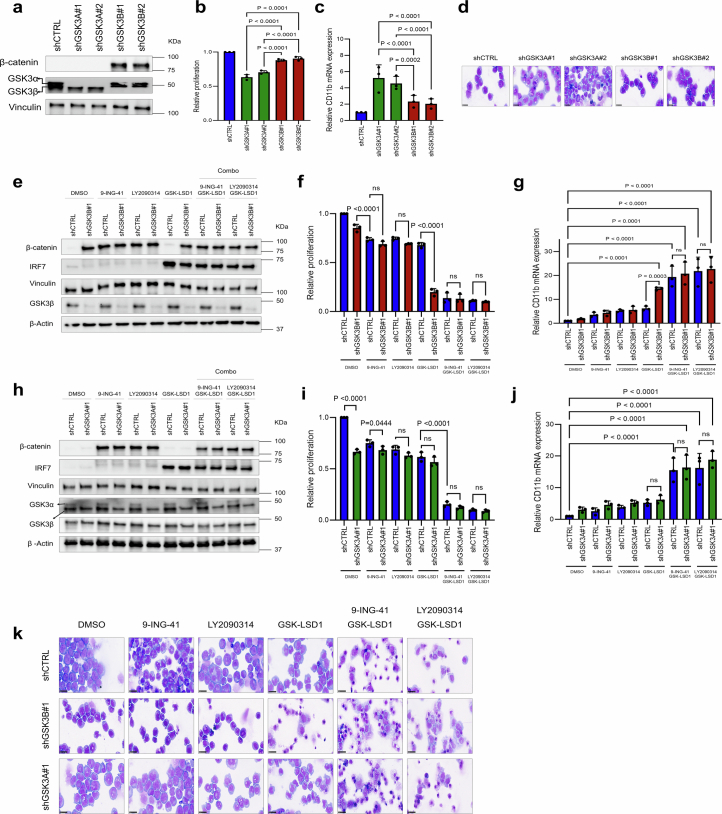
GSK3α and GSK3β depletion have distinct effects on β-catenin stabilization and their synergy with LSD1 inhibitor. **a**, Western blot analysis of GSK3α and GSK3β and β-Catenin protein level in THP-1 cells. Two independent clones were used for *GSK3A*-KD and *GSK3B*-KD. Vinculin was used as a loading control. The experiment was repeated three times with similar results. **b**, Analysis of cell viability in GSK3α- and GSK3β-depleted THP-1 cells using the Cell-Titer-Glo assay. Data are presented as mean ± SD of 3 biological independent experiments (n = 3). *P*-values were determined using two-way analysis of variance (ANOVA). **c**, Analysis of *CD11b* mRNA relative levels in GSK3α- and GSK3β-depleted THP-1 cells. Values were normalized against *GAPDH*. Data are presented as mean ± SD of 3 biological independent experiments (n = 3). *P*-values were determined using two-way analysis of variance (ANOVA). **d**, Representative images of GSK3α- and GSK3β-depleted THP-1 cells stained with Wright-Giemsa. Scale bars, 25 μm. The experiment was repeated three times with similar results. **e**, Western blot analysis of β-Catenin, IRF7, Vinculin, β-Actin and GSK3β protein level in THP-1 cells infected with shCTRL and shGSK3B and treated with indicated inhibitors for 3 days. Vinculin and β-Actin were used as loading controls. The experiment was repeated three times with similar results. **f**, Analysis of cell viability in THP-1 cells infected with shCTRL and shGSK3B and treated with indicated inhibitors for 5 days using the Cell-Titer-Glo assay. Data are presented as mean ± SD of 3 biological independent experiments (n = 3). *P*-values were determined using two-way analysis of variance (ANOVA). **g**, Analysis of *CD11b* mRNA relative levels in THP-1 cells infected with shCTRL and shGSK3B and treated with indicated inhibitors for 5 days. Values were normalized against *GAPDH*. Data are presented as mean ± SD of 3 biological independent experiments (n = 3). *P*-values were determined using two-way analysis of variance (ANOVA). **h**, Western blot analysis of β-Catenin, IRF7, Vinculin, β-Actin, GSK3β and GSK3α protein level in THP-1 cells infected with shCTRL and shGSK3A and treated with indicated inhibitors for 3 days. Vinculin and β-Actin were used as loading controls. The experiment was repeated three times with similar results. **i**, Analysis of cell viability in THP-1 cells infected with shCTRL and shGSK3A and treated with indicated inhibitors for 5 days using the Cell-Titer-Glo assay. Data are presented as mean ± SD of 3 biological independent experiments (n = 3). *P*-values were determined using two-way analysis of variance (ANOVA). **j**, Analysis of *CD11b* mRNA relative levels in THP-1 cells infected with shCTRL and shGSK3A and treated with indicated inhibitors for 5 days. Values were normalized against *GAPDH*. Data are presented as mean ± SD of 3 biological independent experiments (n = 3). *P*-values were determined using two-way analysis of variance (ANOVA). **k**, Representative images of THP-1 cells infected with shCTRL, shGSK3B or shGSK3A, treated with the indicated inhibitors for 5 days, and stained with Wright-Giemsa. Scale bars, 25 μm. The experiment was repeated three times with similar results. “ns” indicates not significant.

The GSK3 gene family consists of two related kinases: GSK3α and GSK3β^36,37^. To determine whether the observed synergy is specific to GSK3α, GSK3β or both, we performed short hairpin RNA (shRNA) knockdowns (Extended Data Fig. 6a). Consistent with previous studies^36,37^, knockdown of the gene encoding GSK3β, but not the gene encoding GSK3α, resulted in an increase in β-catenin levels, whereas depletion of GSK3α led to moderate differentiation in AML cells^36,46^ (Extended Data Fig. 6a–d). Subsequently, we treated the GSK3α-knockdown and GSK3β-knockdown cells with inhibitors. GSK3β knockdown did not affect cell proliferation or differentiation (Extended Data Fig. 6e–g), but increased β-catenin levels (Extended Data Fig. 6e) and synergized with the LSD1i, which elevated IRF7 levels (Extended Data Fig. 6e–g). By contrast, although knockdown of the gene encoding GSK3α led to moderate differentiation, it did not show any synergistic effects with LSD1i (Extended Data Fig. 6h–k), probably because it did not increase the levels of the co-activator β-catenin (Extended Data Fig. 6h). In summary, the synergistic effects of GSK3i with LSD1i are primarily driven by GSK3βi, which elevates the level of β-catenin, thus providing a co-activator to work with key transcription factors (for example, IRF7) to regulate transcription networks that suppress stemness and promote differentiation. As GSK3αi also modestly promotes differentiation, its inhibition by pan-GSK3i may also contribute to the overall effect of the combo treatment in inducing differentiation.

**Fig. 5:**
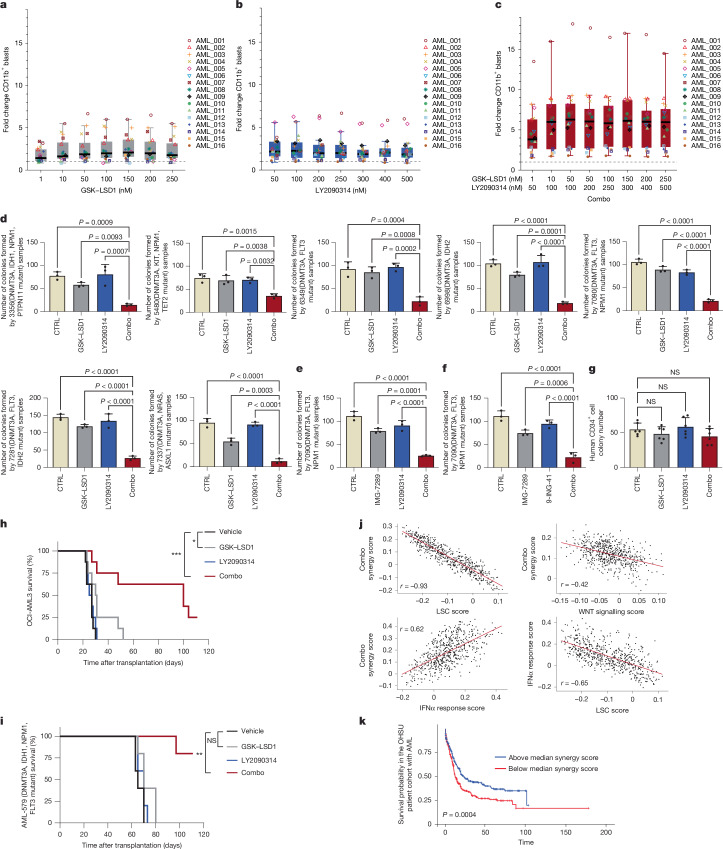
Combo treatment promotes differentiation, reduces the clonogenic potential of human primary AML cells ex vivo and enhances survival in vivo. **a**–**c**, Fold change induction of CD11b^+^ cells in primary AML samples (*n* = 16 biologically independent samples) cultured with varying concentrations of GSK–LSD1 (**a**) or LY2090314 (**b**) or a combination of both inhibitors (**c**) relative to the vehicle (dashed line). Each dot represents one primary sample. See the note for statistical analyses in the statistical analysis section in the Methods. **d**–**f**, Quantification of colonies formed by *DNMT3A*-mutant primary AML samples treated with the indicated inhibitors. Data are presented as mean ± s.d. from three biological independent experiments. *P* values were determined using two-way ANOVA. **g**, Quantification of colonies formed by normal haematopoietic progenitor cells treated with the indicated inhibitors. *P* values were determined using two-way ANOVA. Data are mean ± s.d. from six biologically independent experiments. **h**, Kaplan–Meier survival curves of mice treated with vehicle (*n* = 8), GSK–LSD1 (*n* = 8), LY2090314 (n = 8) or a combination of both inhibitors (*n* = 8) in the OCI-AML3 model. *P* values were determined using the log-rank test. *P = 0.0306 and ***P = 0.0003. **i**, Kaplan–Meier survival curves of mice treated with vehicle (*n* = 5), GSK–LSD1 (*n* = 5), LY2090314 (*n* = 5) or a combination of both inhibitors (*n* = 5) in the *DNMT3A*-mutant AML-579 PDX model. *P* values were determined using the log-rank test. **P = 0.0025. **j**, Correlations between OHSU patient combo synergy enrichment scores, type I IFN response gene enrichment scores, WNT pathway gene enrichment scores and LSC signature gene enrichment scores in the OHSU patient cohort. *r* refers to the Pearson correlation coefficient. **k**, Kaplan–Meier plot showing overall survival of OHSU patients stratified by above and below the median combo synergy signature scores. *P* values were determined using the log-rank test. Source data

We next investigated the effect of combo on a cohort of primary samples from patients with AML cultured ex vivo. Among the 16 patient samples examined for their differentiation potential, 11 exhibited a more than fivefold increase in CD11b^+^ cells and responded more strongly to the combo treatment than to each inhibitor alone (Fig. 5a–c and Supplementary Table 12). *MLL* rearrangements and mutations in *DNMT3A* and *NPM1* were more frequently detected among the sensitive samples, consistent with previous findings that *MLL*-leukaemia are highly sensitive to LSD1i^19^. Out of the 16 samples, 5 did not show a significant response. None had *DNMT3A* mutations. Collectively, these data raise the possibility that *DNMT3A* mutation may have an important role in the response to the combo treatment. In addition, two of these four non-responding patient samples had *TP53* mutations. AMLs with *TP53* mutation have previously been shown to be resistant to LSD1i^47^, which may contribute to their insensitivity to the combo treatment.

**Extended Data Fig. 7:**
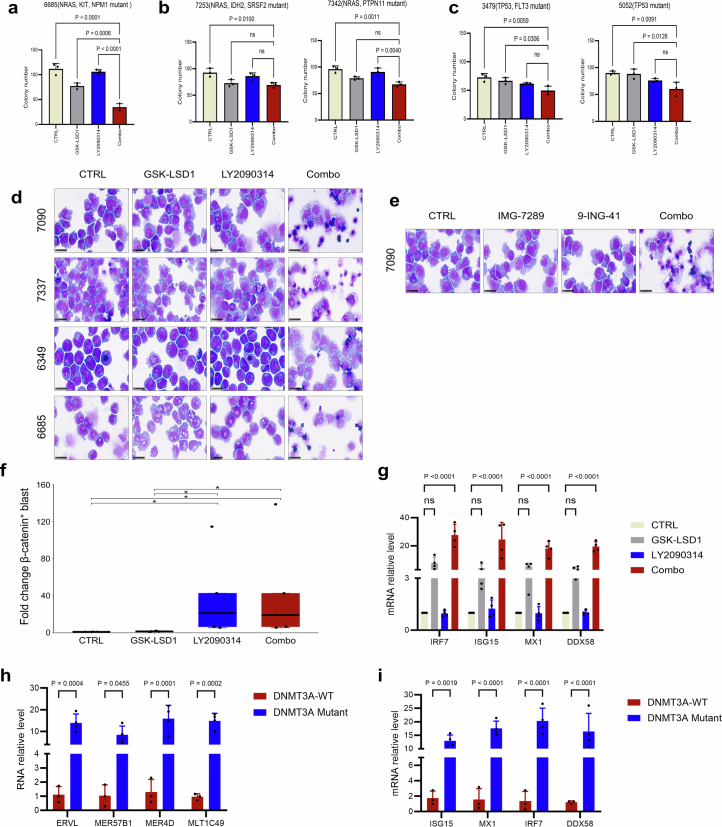
Effect of the combo treatment in different primary AML samples. **a**, Quantification of colonies formed by a *NPM1*-mutant primary AML sample treated with DMSO, GSK-LSD1 (50 nM), LY2090314 (100 nM) or combination of both inhibitors. Data are presented as mean ± SD of 3 biological independent experiments (n = 3). *P*-values were determined using two-way analysis of variance (ANOVA). **b**, Quantification of colonies formed by two different *DNMT3A*-WT primary AML samples treated with DMSO, GSK-LSD1 (50 nM), LY2090314 (100 nM) or combination of both inhibitors. Data are presented as mean ± SD of 3 biological independent experiments (n = 3). *P*-values were determined using two-way analysis of variance (ANOVA). **c**, Quantification of colonies formed by two different *TP53*-mutant primary AML samples treated with DMSO, GSK-LSD1 (50 nM), LY2090314 (100 nM) and combination of both inhibitors. Data are presented as mean ± SD of 3 biological independent experiments (n = 3). *P*-values were determined using two-way analysis of variance (ANOVA). **d**, Wright-Giemsa-stained cytospins for primary AML samples treated with DMSO, GSK-LSD1 (50 nM), LY2090314 (100 nM) and combination of both inhibitors. Scale bars, 25 μm. The experiment was repeated three times with similar results. **e**, Wright-Giemsa-stained cytospins for a *DNMT3A*-mutant primary AML sample treated with DMSO, IMG-7289 (50 nM), 9-ING-41 (100 nM) and combination of both inhibitors. Scale bars, 25 μm. The experiment was repeated three times with similar results. **f**, Fold change induction of β-catenin-positive cells in primary AML samples (n = 5 biologically independent samples) treated with Vehicle, GSK-LSD1 (50 nM), LY2090314 (100 nM) or combination of both inhibitors. For statistical analyses two-sided Mann Whitney U tests were used * = *P* < 0.05. Comparisons: DMSO - LY2090314 *p*-value = 0.042, DMSO – Combo *p*-value = 0.042, GSK-LSD1 - LY2090314 *p*-value = 0.048, GSK-LSD1- Combo *p*-value 0.048. *P*-values were adjusted with Bonferroni method. In the boxplot, the middle line represents the median, the lower and upper hinges represent the 25th and 75th percentiles, the lower whisker extends from the lower hinge to the smallest value at most 1.5*inter-quartile range (IQR) of the hinge, the upper whisker extends from the upper hinge to the largest value no further than 1.5*IQR of the hinge. Data beyond the whiskers are outliers that are plotted individually. **g**, Analysis of *IRF7*, *ISG15*, *DDX58* and *MX1* mRNA relative levels in *DNMT3A*-mutant primary AML samples treated with DMSO, GSK-LSD1 (50 nM), LY2090314 (100 nM) or combination of both inhibitors. Values were normalized against *GAPDH*. Each dot represents one primary sample and n = 3 biological independent experiments. *P*-values were determined using two-way analysis of variance (ANOVA). **h**, Analysis of ERVs mRNA relative levels in *DNMT3A*-WT vs *DNMT3A*-mutant primary AML samples. Each dot represents one primary sample and n = 3 biological independent experiments. *P*-values were determined using two-way analysis of variance (ANOVA). **i**, Analysis of *IRF7*, *ISG15*, *DDX58* and *MX1* mRNA relative levels in a *DNMT3A*-WT vs *DNMT3A*-mutant primary AML samples. Values were normalized against *GAPDH*. Each dot represents one primary sample and n = 3 biological independent experiments. *P*-values were determined using two-way analysis of variance (ANOVA). “ns” indicates not significant.

**Extended Data Fig. 8:**
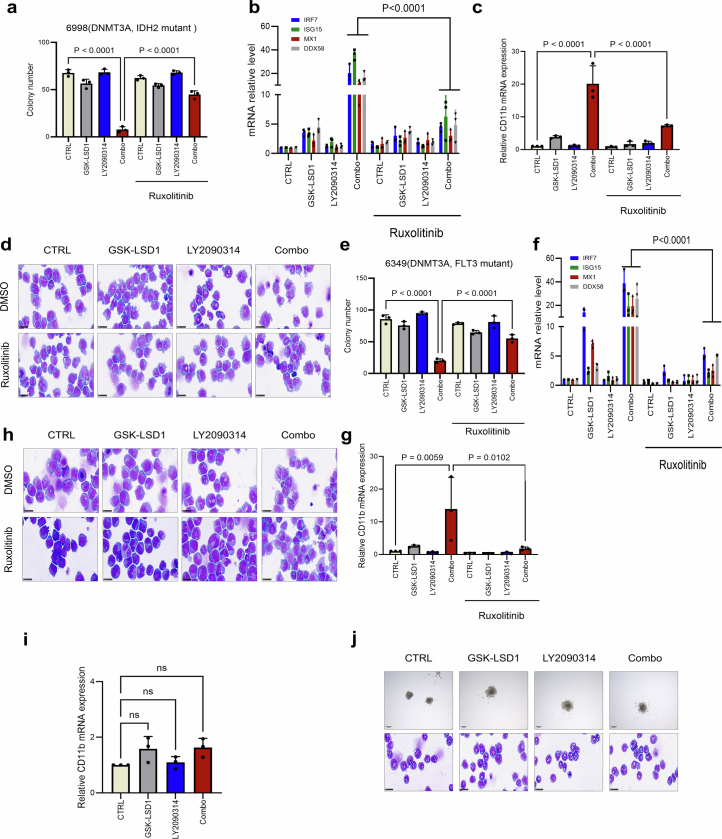
Ruxolitinib treatment abrogates the effect of the combo treatment in primary AML samples. **a**, Quantification of colonies formed by a primary AML sample (6998) treated with DMSO, GSK-LSD1 (50 nM), LY2090314 (100 nM) or combination of both inhibitors in the presence or absence of ruxolitinib. Data are presented as mean ± SD of 3 biological independent experiments (n = 3). *P*-values were determined using two-way analysis of variance (ANOVA). **b**, Analysis of *IRF7*, *ISG15, MX1* and *DDX58* mRNA relative levels in the primary sample (6998) treated with indicated inhibitors in the presence or absence of ruxolitinib. Values were normalized against *GAPDH*. Data are presented as mean ± SD of 3 biological independent experiments (n = 3). *P*-values were determined using two-way analysis of variance (ANOVA). **c**, Analysis of *CD11b* mRNA relative levels in the primary sample (6998) treated with the indicated inhibitors in the presence or absence of ruxolitinib. Values were normalized against *GAPDH*. Data are presented as mean ± SD of 3 biological independent experiments (n = 3). *P*-values were determined using two-way analysis of variance (ANOVA). **d**, Representative images of the AML primary sample (6998) treated with indicated inhibitors in the presence or absence of ruxolitinib and stained with Wright-Giemsa. Scale bars, 25 μm. The experiment was repeated three times with similar results. **e**, Quantification of colonies formed by a primary AML sample (6349) treated with DMSO, GSK-LSD1 (50 nM), LY2090314 (100 nM) and combination of both inhibitors in the presence or absence of ruxolitinib. Data are presented as mean ± SD of 3 biological independent experiments (n = 3). *P*-values were determined using two-way analysis of variance (ANOVA). **f**, Analysis of *IRF7*, *ISG15, MX1* and *DDX58* mRNA relative levels in the primary sample (6349) treated with indicated inhibitors in the presence or absence of ruxolitinib. Values were normalized against *GAPDH*. Data are presented as mean ± SD of 3 biological independent experiments (n = 3). *P*-values were determined using two-way analysis of variance (ANOVA). **g**, Analysis of *CD11b* mRNA relative levels in the primary sample (6349) treated with the indicated inhibitors in the presence or absence of ruxolitinib. Values were normalized against *GAPDH*. Data are presented as mean ± SD of 3 biological independent experiments (n = 3). *P*-values were determined using two-way analysis of variance (ANOVA). **h**, Representative images of the AML primary sample (6349) treated with indicated inhibitors in the presence or absence of ruxolitinib and stained with Wright-Giemsa. Scale bars, 25 μm. *P*-values were determined using two-way analysis of variance (ANOVA). The experiment was repeated three times with similar results. **i**, Analysis of *CD11b* mRNA relative levels in CD34^+^ cells treated with the indicated inhibitors. Values were normalized against *GAPDH*. Data are presented as mean ± SD of 3 biological independent experiments (n = 3). *P*-values were determined using two-way analysis of variance (ANOVA). “ns” indicates not significant. **j**, Morphology of CD34^+^ cells and their colonies treated with indicated inhibitors. Scale bars, 50 μm for colonies morphology and 25 μm for morphology of cells. The experiment was repeated three times with similar results.

We further investigated the effect of the inhibitors on the clonogenic potential of an additional 12 primary samples from patients with AML, 7 of which had a *DNMT3A* mutation (Fig. 5d), whereas the other 5 were *DNMT3A* wild type (WT; Extended Data Fig. 7a–c). The combo treatment significantly suppressed the clonogenic potential of all *DNMT3A*-mutant samples, regardless of secondary mutations (Fig. 5d). In addition to *DNMT3A*, samples with an *NPM1* mutation also appeared to respond to the combo treatment (Extended Data Fig. 7a), whereas *DNMT3A*-WT and *TP53*-mutant samples appeared insensitive (Extended Data Fig. 7b,c). Combo-treated cells also had morphological characteristics of mature granulocytes (Extended Data Fig. 7d). Similar results were obtained using the combination of bomedemstat and 9-ING-41 (Fig. 5e,f and Extended Data Fig. 7e). Similar to human AML cell lines, in these primary human AML samples, we also found that LY2090314 and combo treatment stabilized β-catenin, with the combo strongly upregulating ISG expression (Extended Data Fig. 7f,g). It has been shown that AML samples with a *DNMT3A* mutation exhibit elevated levels of endogenous retroelements and increased susceptibility to viral mimicry induced by azacytidine^48^. We confirmed elevated expression of repetitive elements and ISGs in our *DNMT3A*-mutant AML samples (Extended Data Fig. 7h,i), suggesting that activity of an already elevated IFN pathway may sensitize the responsiveness of patient cells to further pathway activation induced by combo treatment. Consistent with the findings in AML cell lines, ruxolitinib also suppressed the effects of the combo treatment in primary cells (Extended Data Fig. 8a–h). Treatment with the inhibitors at the same concentrations had no effect on the colony formation and differentiation of normal human haematopoietic cells (Fig. 5g and Extended Data Fig. 8i,j). This suggests that the combo treatment selectively targets leukaemia cells with minimal effects on normal haematopoietic cells.

**Extended Data Fig. 9:**
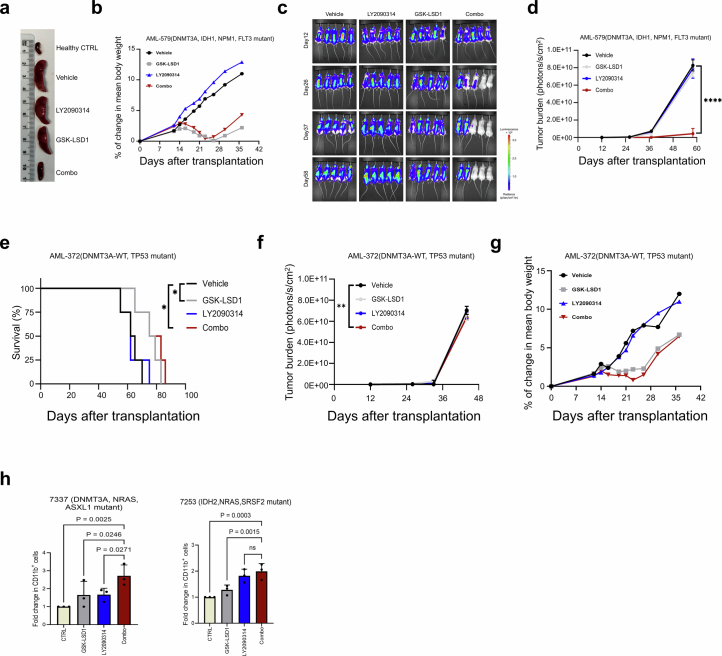
Combo treatment efficacy in vivo. **a**, Related to Fig. 5h. Representative images of spleen harvested from mice at the end of treatment in OCI-AML3 model. **b**, **c**, **d**, related to Fig. 5i. Body weight variance (**b**), representative bioluminescence images (**c**) and quantitative bioluminescence imaging data (**d**) for mice treated with indicated inhibitor in *DNMT3A*-mutant PDX sample, AML-579. **** = p < 0.0001. **e**, **f**, **g**, Kaplan–Meier survival curves (**e**), tumor burden (**f**) and body weight variance (**g**) of vehicle (n = 4), GSK-LSD1 (n = 4), LY2090314 (n = 4) or combination of both inhibitors (n = 4)–treated mice in the *DNMT3A*-WT PDX sample, AML-372. Treatment was initiated 13 days after transplantation for two weeks. *P*-values were determined using the log-rank test. * = p = 0.0344, ** = p = 0.0096. **h**, Fold change in CD11b^+^ cells among patient-derived cells isolated from human organoids after 5 days of treatment with vehicle, GSK-LSD1, LY2090314 or combination therapy. Data are presented as mean ± SD of 3 biological independent experiments (n = 3). *P*-values were determined using two-way analysis of variance (ANOVA). Source data

Next, we assessed the therapeutic potential of the combo treatment in in vivo models of an AML cell line and *DNMT3A*-mutated and *DNMT3A*-WT patient-derived xenografts (PDXs)^49–51^. We first used the OCI-AML3 xenotransplantation model, which carries both *NPM1* and *DNMT3A* mutations and serves as an aggressive model of *DNMT3A*–*NPM1*-mutated AML, in which terminal disease develops within 3 weeks of transplantation^49^. Vehicle-treated mice rapidly developed terminal leukaemia, with a median survival of 26 days (Fig. 5h). However, only the combo treatment significantly extended survival (Fig. 5h) and reduced splenomegaly (Extended Data Fig. 9a). We further evaluated the combo in a PDX model of *DNMT3A*-mutated leukaemia (AML-579)^50,51^. Treatment began 13 days post-transplantation and continued for 2 weeks, during which no overt toxicity or body weight loss was observed (Extended Data Fig. 9b). In mice bearing AML-579, the combo treatment significantly reduced tumour burden, as measured by in vivo bioluminescence imaging (Extended Data Fig. 9c,d), and markedly increased survival, with three out of five mice achieving complete leukaemia clearance (Fig. 5i). By contrast, in a PDX model of *DNMT3A*-WT leukaemia (AML-372), although both LSD1i and the combo treatment led to a slight increase in survival and reduction in tumour burden, the combo treatment did not show any additional benefits over LSD1i alone (Extended Data Fig. 9e,f). Consistently, no significant toxicity or weight loss was observed in combo-treated mice (Extended Data Fig. 9g).

To validate the effectiveness of the combo treatment in promoting differentiation within a multicellular haematopoietic microenvironment, we used a three-dimensional model that more accurately mimics the human bone marrow tissue environment^52,53^. The combo treatment significantly increased the population of CD11b^+^ cells compared with the DMSO-treated control and single-treatment conditions in the *DNMT3A*-mutant sample (Extended Data Fig. 9h). In the *DNMT3A*-WT sample, although the combo treatment led to a higher number of CD11b^+^ cells than the control, the single treatment, LY2090314, also resulted in a similar response (Extended Data Fig. 9h). In summary, results from multiple AML models demonstrate that the proposed combo treatment exhibits significant in vivo activity, evidenced by reduced leukaemia growth and prolonged survival in *DNMT3A*-mutated xenograft models. In *DNMT3A*-WT samples, although the combo treatment modestly increased survival and induced CD11b expression, its effect was not superior to that of single-agent treatment. Further research is warranted to investigate how we can enable patients with non-responsive, *DNMT3A*-WT respond to combo treatment, potentially by combining the combo treatment with hypomethylating agents^54–56^.

**Extended Data Fig. 10:**
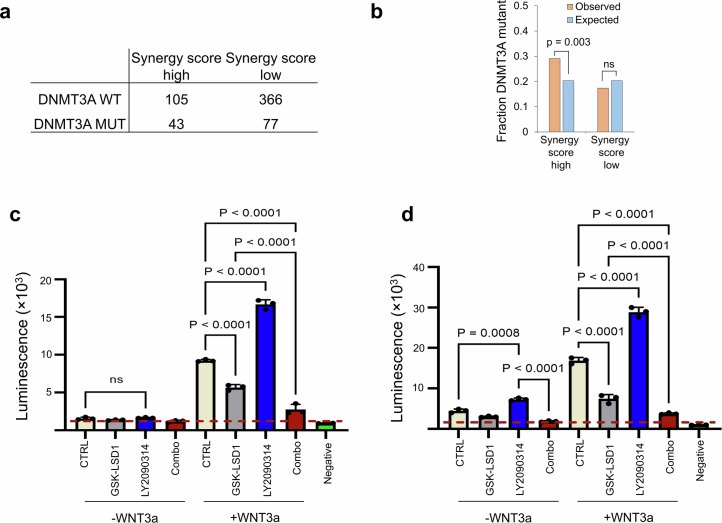
Combo treatment suppresses the Wnt pathway in AML and colorectal cells. **a**, Number of *DNMT3A-*WT and *DNMT3A-*mutant OHSU patients with high (upper quartile) combo synergy scores and low (quartiles 1-3) combo synergy scores. **b**, data in (**a**) shown via bar graph. P-value reports significance of overrepresentation of DNMT3A mutant patients in the synergy score high population using one-proportion z-tests. “ns” indicates not significant. **c**, Analyzing the TCF/LEF reporter activity in THP-1 cells following treatment with indicated inhibitors, with and without the presence of WNT3a. Data are presented as mean ± SD of 3 biological independent experiments (n = 3). *P*-values were determined using two-way analysis of variance (ANOVA). **d**, Analyzing the TCF/LEF reporter activity in HCT-116 cells following treatment with indicated inhibitors, with and without the presence of WNT3a. Data are presented as mean ± SD of 3 biological independent experiments (n = 3). *P*-values were determined using two-way analysis of variance (ANOVA).

Finally, we investigated whether our findings have clinical relevance by scoring patients of the OHSU dataset according to enrichment of the drug combo synergy signature, as well as signatures for LSCs, the type I IFN and WNT signalling. Consistent with predictions from our experimental studies, patient synergy signature scores positively correlated with the type I IFN pathway signature scores (*r* = 0.62; *P* < 0.0001) and strongly negatively correlated with the LSC signature (*r* = −0.93; *P* < 0.0001) and the WNT signalling pathway (*r* = −0.42; *P* < 0.0001) scores. As expected, the type I IFN pathway scores also negatively correlated with the LSC signature scores (*r* = −0.65, *P* < 0.0001; Fig. 5j). As patients with *DNMT3A* mutation were most responsive to combo treatment, we also investigated the correlation between *DNMT3A* status and synergy scores in the OHSU cohort. Patients with *DNMT3A* mutation were significantly more likely to enrich the synergy signature than patients with *DNMT3A*-WT (Extended Data Fig. 10a,b). Finally, we investigated whether the synergy signature had prognostic value, reasoning that greater blast maturation would correspond to less-aggressive disease. Indeed, patients with above median synergy had significantly longer overall survival than patients with below median synergy (Fig. 5k).

The possibility that combo treatment may be actively suppressing the WNT pathway has profound ramifications for cancers beyond AML that are driven by canonical WNT signalling^57^. However, interpretation of WNT pathway-related results in ER-HOXA9 and THP-1 cells is complicated by the fact that they have low endogenous WNT pathway activity (Extended Data Fig. 10c). To circumvent this, we generated a THP-1 line carrying a TCF/LEF reporter, which reports WNT activity^58^. Although the reporter showed no detectable expression under basal condition, the addition of recombinant WNT3A strongly activated reporter expression, which was suppressed by the combo treatment (Extended Data Fig. 10c). To further determine whether the combo treatment can suppress WNT signalling, we used a WNT pathway-driven HCT116 colorectal cancer cell line carrying a TCF/LEF reporter. Combo treatment not only repressed the TCF reporter under basal condition but also actively suppressed recombinant WNT3A-induced WNT hyperactivation (Extended Data Fig. 10d). It will be of notable interest to determine whether this observation can be recapitulated in different WNT-driven oncogenic contexts in future experiments.

## Discussion

Differentiation arrest is a hallmark of AML and represents a therapeutic vulnerability^1,2^. Although differentiation therapy with ATRA/ATO is effective in APL^3^, its broader applicability in AML remains unclear. LSD1 inhibitors induce AML differentiation but have shown limited clinical success due to associate toxicity^19,21,22^. Here we demonstrate that simultaneous LSD1i and GSK3i robustly promotes therapeutic differentiation of AML cells.

Although GSK3i has shown preclinical promise^26,27,59^, its clinical utility is complicated by insufficient efficacy and by β-catenin stabilization and activation of WNT–β-catenin signalling, as this signalling pathway is crucial for maintaining the LSC population^60^ and drug resistance^61^. Suppressing β-catenin delays disease progression in MLL-rearranged leukaemia^61^, but its role in primary AML varies^62^, suggesting subtype-specific effects. Furthermore, although GSK3 loss in haematopoietic progenitors has been linked to aggressive myelodysplasia and AML^63^, recent studies have argued that these effects may stem from tamoxifen use in mouse models and GSK3βi could be a viable therapeutic strategy^64,65^. Further research is needed to fully understand how GSK3i alone affects AML and if there are AML subtypes that would respond more favourably to GSK3i. As GSK3i alone has not shown sufficient clinical efficacy^29^, our findings are therefore important, as they suggest a new strategy that involves simultaneous GSK3i and LSD1i, which leads to therapeutically important maturation of AML cells while suppressing the WNT pathway. We also discovered the underlying molecular mechanism in which only the combo treatment induces expression and promotes co-occupancy of key transcription factors such as IRF7 and the co-activator β-catenin to drive transcription of genes in the type I IFN signalling pathway such as STAT1, which is critical for AML differentiation^42–44^. STAT1, which we showed to be necessary for the combo treatment to induce differentiation, not only mediates an IFN response but also activates IFN-independent signalling^66,67^. STAT1 has been reported to control the cell cycle by modulating the expression of cyclin kinase inhibitors as well as various cyclins^68^. In addition, STAT1 is important in inhibiting the expression of MYC^68^. Therefore, the ability of STAT1 to activate IFN-independent signalling in addition to IFN response and cell-cycle regulation may also contribute to the overall differentiation response of AML cells.

As LSD1 and GSK3 inhibitors are both in clinical trials for a range of myeloid malignancies and advanced/metastatic cancer, respectively, their use as a combination therapy could conceivably enter the clinic in the near term. Finally, the unique ability of this combination strategy to suppress the canonical WNT pathway and re-route transcriptional programs to promote differentiation may also represent an unexplored therapeutic avenue of promise for numerous WNT-driven cancers.

## Methods

### Small-molecule inhibitor screen

ER-HOXA9 cells were prepared in media with 50 nM GSK–LSD1 (Selleck Chemical) and seeded at a density of 50,000 cells per millilitre in 200 µl volume per well of a flat-bottom 96-well plastic plate (Genesee Scientific) using a Combi Reagent Dispenser (Thermo Fisher). Drugs in 100% DMSO (Sigma-Aldrich) were pin transferred (V&P Scientific) from 384-well stock plates into the 96-well plates containing our cells at approximately 300 nl drug stock per well. Cells treated with GSK–LSD1 alone served as negative control, whereas cells treated with GSK–LSD1 and 100 nM cytarabine (Sigma-Aldrich), a known synergistic combination, served as a positive control. Plates were incubated for 5 days and analysed on an iQue Screener Plus-VBR flow cytometer (Intellicyt) running the Forecyt acquisition and analysis software (v9.0). Monocytic differentiation was assessed using an internal Lyz2–GFP marker (blue laser channel at 488-nm excitation and 530-nm emission). Viability was calculated by dividing the number of live cells by the number of total cells, and differentiation was calculated by dividing the number of Lyz2–GFP^+^ cells by the number of live cells.

### Cell culture

ER-HOXA9 cells were grown in RPMI-1640 medium supplemented with 10% of fetal bovine serum (FBS), 100 ng ml^−1^ stem cell factor (SCF; 78064, Stemcell Technologies), 4 mM glutamine, 1% penicillin–streptomycin and 0.5 mM β-oestradiol (E2; E4389, Sigma-Aldrich). HOXA9–MEIS1 cells were similarly passaged as ER-HOXA9 cells except without E2. RN2 cells were cultured in RPMI-1640 medium supplemented with 10% FBS, 20 mM l-glutamine, 10 mM sodium pyruvate, 10 mM HEPES (pH 7.3), 1% penicillin–streptomycin and 50 μM β-mercaptoethanol.

THP-1 and U937 were grown in RPMI-1640 medium supplemented with 10% of FBS, 4 mM glutamine and 1% penicillin–streptomycin. MOLM-13 and Kasumi-1 were grown in RPMI-1640 medium supplemented with 20% of FBS, 4 mM glutamine and 1% penicillin–streptomycin. OCI-AML2 and OCI-AML3 were grown in α-MEM (with ribonucleosides and deoxyribonucleosides) with 20% FBS, 4 mM glutamine and 1% penicillin–streptomycin. Cells were maintained at 37 °C and 5% CO_2_ with routine testing to confirm lack of mycoplasma infection.

### Drug combination assay and synergy score analysis

The drug synergy assay was performed as previously described^69^. In brief, cells were seeded into 96-well plates and exposed to various concentrations of inhibitors, both individually and in combination. Cell viability was quantified using Cell-Titer-Glo (Promega) and normalized to DMSO to calculate the inhibitory response. The resulting data were analysed using the SynergyFinder web application (https://synergyfinder.fimm.fi), which generated dose–response matrices for each drug combination. Synergy scores were calculated using the highest single-agent model to assess drug interactions. Heatmaps were generated to visualize the results, with the following interpretation thresholds: synergy scores below −10 indicated antagonism, scores between −10 and 10 suggested an additive effect, and scores above 10 indicated synergy.

### Human primary AML samples

Bone marrow or peripheral blood samples were collected after informed consent from patients with AML using protocols approved by an Institutional Review Board at the Helsinki University Hospital (permit numbers 239/13/03/00/2010 and 303/13/03/01/2011) in compliance with the Declaration of Helsinki. Mononuclear cells were isolated from bone marrow or peripheral blood samples by Ficoll-Paque Premium (GE Healthcare) density gradient separation and viably frozen and stored in liquid nitrogen before further analyses.

### LSK cell sorting and colony formation

Eight-week-old C57BL/6J mice were euthanized, and bone marrow was harvested from the femurs and tibias of both legs. Haematopoietic progenitor cells were enriched using the EasySep Mouse Hematopoietic Progenitor Cell Isolation Kit (19856, StemCell Technologies). Haematopoietic progenitor-enriched cells were then stained with antibodies to mouse KIT (clone: ACK2, 567818, BD Bioscience), Sca-1 (clone: E13-161.7, 753334, BD Bioscience), lineage markers (CD3, CD11b, CD19, B220, Gr1 and Ter119; 155606, 101212, 115512, 103212, 108412 and 116212, respectively, BioLegend), and Fixable Viability Stain 575V (565694, BD Bioscience). LSK cells were sorted using a FACS Aria Fusion (BD Bioscience). For the colony formation assay, 2,000 LSK cells were resuspended in 100 μl of IMDM and added to 1.5 ml of methylcellulose media (M3434, StemCell Technologies). Colonies were counted after 10 days of incubation at 37 °C. ImageJ (v1.54g) was used for the analysis of colonies morphology images.

### Ex vivo drug sensitivity testing of primary AML cells

GSK–LSD1 and LY2090314 (MedChem Express) were dissolved in 100% DMSO and dispensed on Nunc 96-well polystyrene V-bottom plates (Thermo Fisher Scientific) using the Echo 550 Acoustic Dispenser (Labcyte) in seven different concentrations. GSK–LSD1 was plated in a concentration range of 1–250 nM and LY2090314 in a range of 50–500 nM as single agents and in combination. 0.1% DMSO was used as a negative control, and 100 µM benzethonium chloride (Sigma-Aldrich) was used as a positive control for total cell death.

Frozen mononuclear cells were thawed and suspended in 12.5% conditioned medium composed of RPMI-1640 medium (Corning) supplemented with 12.5% HS-5 cell-derived conditioned medium, 10% FBS 2 mM l-glutamine and penicillin–streptomycin (100 U ml^−1^)^70^, and then treated with DENARASE (250 U µl^−1^, c-LEcta) to degrade DNA released from dead cells; the cells were left to recover for 4 h in 12.5% conditioned medium. The cells were plated onto pre-drugged plates at a density of 50,000 cells per well and incubated with the drugs for 5 days (at 37 °C at 5% CO_2_). After incubation, the cells were centrifuged (at 500*g* for 5 min) and resuspended in staining buffer (RPMI-1640, 10% FBS, 2 mM l-glutamine and 100 U ml^−1^ penicillin–streptomycin). The cells were stained with antibodies to CD45–FITC (BD Pharmingen), CD34–APC (BD Pharmingen), CD15–PE–Cy7 (BioLegend), CD14–BV421 (BD Biosciences) and CD11b–BV605 (BD Horizon) for 30 min at room temperature in the dark. Subsequently, the cells were centrifuged (at 500*g* for 5 min) and excess antibodies were removed. The cells were resuspended and stained with PE annexin V and seven-amino actinomycin D in annexin V-binding buffer (BD Pharmingen) for 15 min at room temperature in the dark. The cells were analysed using the iQue Screener Plus-VBR flow cytometer, and gating was done with ForeCyt software (version 9.0, Intellicyt). Data were processed and analysed using R software (v4.2).

### Seeding of organoids with primary patient samples and drug treatment

Human bone marrow organoids were generated from a fluorescent human induced pluripotent stem (iPS) cell line as previously described^52,53^. The fluorescent iPS cell line, MCND-TENS2-mScarlet3, was obtained through CRISPR–Cas9-mediated knock-in of mScarlet3 at the *AAVS1* safe harbour locus in the parental line MCND-TENS2 (registered at https://hpscreg.eu/cell-line/RTIBDi001-A), performed by the iPS Cell Core Facility at the US National Institutes of Health (NIH). On day 14 of organoid differentiation, individual mScarlet^+^ organoids were seeded into 96-well ultra-low attachment plates. Cryopreserved mononuclear cells from samples from patients with AML were then engrafted into the organoids at a density of 10,000 cells per organoid, with 8 organoids for each treatment condition. The organoids were subsequently cultured in StemPro-34 SFM medium (10639011, Thermo Fisher Scientific) supplemented with 2% KnockOut serum (10828028, Thermo Fisher Scientific), 2% chemically defined lipids (11905031, Thermo Fisher Scientific), 0.5% penicillin–streptomycin and cytokines (10 ng ml^−1^ of SCF, FLT3-L, TPO, IL-6, G-CSF and GM-CSF, and 5 ng ml^−1^ of IL-3). Twenty-four hours post-engraftment, the engrafted organoids were treated with either vehicle control, 25 nM GSK–LSD1, 50 nM LY2090314 or a combination of inhibitors for 5 days. Following treatment, the eight organoids from the same condition were pooled to minimize variations, and they were then dissociated using collagenase D (11088866001, Roche)^71^ and analysed by flow cytometry to determine the percentage of mScarlet^−^CD11b^+^ cells.

### Phosphoflow analysis

After thawing and DENARASE treatment as previously described, mononuclear cells from samples from patients with AML were plated onto pre-drugged Nunc 96-well V-bottom plate at a density of 200,000 cells per well and incubated with 50 nM GSK–LSD1, 100 nM LY2090314 and the combination of both drugs for 5 days (at 37 °C at 5% CO_2_). After incubation with the drugs, the cells were washed with PBS, centrifuged (at 1,000*g* for 4 min) and stained with Zombie Yellow (BioLegend) viability marker for 30 min in the dark at room temperature. The cells were washed with staining buffer (5% FBS in Dulbecco’s phosphate-buffered saline (DPBS)) and stained with surface markers for CD45–BV786 (BD Biosciences), CD38–BV421 (BD Biosciences), CD34–APC–Cy7 (BioLegend) and CD11b–BV605 (BD Horizon) for 30 min at room temperature. The cells were fixed in 1.5% paraformaldehyde solution in PBS pre-warmed to 37 °C for 15 min at room temperature. Fixed cells were centrifuged (at 1,000*g* for 4 min), washed with staining buffer and centrifuged again with the same settings. The cells were resuspended in ice-cold methanol and incubated at 4 °C for 30 min, after which the cells were washed twice with staining buffer with centrifugation (at 1,000*g* for 4 min). The cells were stained with β-catenin–AF488 (BD Pharmingen) for 1 h at room temperature. After incubation, the cells were washed with staining buffer, centrifuged (at 1,000*g* for 4 min), then resuspended and analysed on an iQue PLUS flow cytometer, and data were analysed using the Forecyt software (v9.0). Data were processed and analysed using R software (v4.2).

### In vivo study

HOXA9–MEIS1-overexpressing leukaemia cells previously developed by Sykes et al.^24^ were virally transduced to express Luciferase and GFP for in vivo tracking. Cells expressing GFP were twice sorted and used to establish syngeneic mouse models of AML in 6–8-week-old female C57BL/6J mice purchased from Jackson Laboratory. Animals were maintained at Boston Children Hospital’s ARCH facility and treated according to all protocols approved by the Institutional Animal Care and Use Committee under protocol number 16-09-3230R. The mice received sublethal radiation of 350 cGy 16–20 h before tail-vein injection of 0.5 × 10^4^ leukaemia cells in 100 µl PBS to establish a measurable residual disease model of AML. Leukaemia engraftment and therapy response were monitored using whole-body IVIS imaging through retro-orbital injection of luciferin. Mice were randomized into four treatment groups after engraftment was observed 7 days post-injection.

NSG (NOD.Cg-Prkdc^SCID^ Il2rgt^m1Wjl^/SzJ) mice were purchased from Charles River and irradiated with a sublethal dose of 1.5 Gy 24 h before intravenous injection. For the OCI-AML3 model, approximately 1 million cells were transplanted via tail-vein injection into 6–8-week-old male or female NSG recipient mice. To assess leukaemia development, peripheral blood was collected from the mice, stained for human CD45 and analysed by flow cytometry; treatment was initiated 13 days after transplantation, once hCD45^+^ cells were detected in the peripheral blood. For luciferase-expressing PDX samples, AML-372 (*DNMT3A*-WT) and AML-579 (*DNMT3A*-mutant) models^50,51^, about 700,000 and 1 million cells, respectively, were transplanted via tail-vein injection into 6–8-week-old female NSG recipient mice and treatment was initiated 13 days after transplantation. Engraftment and leukaemia burden were evaluated using a bioluminescence imaging system following the intravenous administration of d-luciferin (P1043, Promega). For in vivo treatments, either GSK–LSD1 (0.25 mg kg^−1^), LY2090314 (10 mg kg^−1^) or a combination of both was administered via intraperitoneal injections every alternate day for 1 week (HOXA9–MEIS1 model) or 2 weeks (OCI-AML3 model and PDX models). Animals were monitored daily, and body weights were measured throughout the treatment period. Mice exhibiting signs of distress, rough fur, hunchback and reduced motility were euthanized by a schedule 1 method. Kaplan–Meier survival curves were generated using GraphPad Prism (v10) software. All cages were on a 12-h–12-h light–dark cycle (lights on at 07:00) in a temperature-controlled and humidity-controlled room. Room temperature was maintained at 19–23 °C, and room humidity was maintained at 45–65%. All mouse procedures were carried out in accordance with UK Animals (Scientific Procedures) Act 1986 and University of Oxford Animal Welfare and Ethical Review Body approval under Project license (PPL) number PP4128654.

### Dot blots

Purified total RNA from treated cells were subjected to digestion with mock, RNase T1 (AM2283, Thermo Fisher Scientific) and RNase III (AM2290, Thermo Fisher Scientific) in their respective buffers and according to the manufacturer’s instructions, or RNase A (EN0531, Thermo Fisher Scientific) under high-salt condition (350 mM NaCl). The digestion was deactivated by the addition of TRIzol and RNA samples extracted using the TRIzol manufacturer’s protocol (R2053, Zymo Research). Equal volumes (3 µl) of purified RNA were dotted on Hybond N^+^ membrane (RPN119B, GE Healthcare), air dried for 10–15 min at room temperature, then UV crosslinked in a UV stratalinker 2400 (Stratagene) two times. The membrane was blocked for 1 h in blocking buffer (5% milk diluted in 0.01% PBS-T) and probed with J2 antibody (RNT-SCI-10010500, Jena Bioscience) rocking overnight at 4 °C. On the next day, the membrane was washed three times in PBS-T, rocking for 10 min at room temperature per wash and probed with secondary goat-anti-mouse horseradish peroxidase (HRP) antibody in 5% milk at room temperature for 1 h. Membrane was washed three times in PBS-T, rocking for 10 min at room temperature per wash, and enhanced chemiluminescence (ECL) was applied for chemiluminescent development. To detect total nucleic acid loading, the membrane was then incubated in 0.5% methylene blue in 30% EtOH to visualize the presence of RNA.

### CRISPR–Cas9 gene knockouts

CRISPR gene editing was performed using the Integrated DNA Technologies (IDT) Alt-R CRISPR–Cas9 System as per the manufacturer’s protocol. In brief, Alt-R CRISPR–Cas9 CRISPR RNA (crRNA) was mixed with Alt-R CRISPR–Cas9 trans-activating crRNA (tracrRNA) and Alt-R HiFi S.p. Cas9 Nuclease V3 to assemble the ribonucleoprotein complex. Subsequently, this complex was electroporated into target cells using the Neon transfection system, using a pulse voltage of 1,400, a width of 10 ms and three pulses. Alt-R CRISPR–Cas9 negative control crRNA #2 was used for the creation of non-targeted controls. Specific gene knockouts were generated using guide RNAs listed in Supplementary Table 13 that were selected using the IDT predesign and selection tool.

### Gene knockdown by shRNA

Target sequences for shRNA knockdown of the genes encoding GSK3α and GSK3β were sourced from existing literature^36^ and oligos were ordered from IDT. In brief, shRNA oligos were annealed and ligated into the pLKO.1-Puro (Addgene #10878) plasmid backbone (digested with AgeI and EcoRI) overnight and subsequently transformed into NEB stable-competent *Escherichia* *coli* (C3040H, NEB). Colonies were screened for correct insertion using primers flanking approximately 100 bp upstream and downstream of the AgeI and EcoRI restriction sites. Positive clones were isolated and sent for sequencing before co-transfection with pCMV-dR8.2 and pCMV-VSVG into HEK293T cells for lentivirus production.

Target sequences used for shRNA knockdown experiments listed in Supplementary Table 14.

### Total RNA extraction and RT–PCR

Total RNA isolation and DNaseI treatment was performed using the Direct-zol RNA MiniPrep kit (R2053, Zymo Research) as per the manufacturer’s protocol. Reverse transcription of 1 μg of RNA per sample was performed using SuperScript IV Vilo (11756050, Thermo Fisher Scientific) as per the manufacturer’s protocol and quantified by spectrophotometer (ND1000 NanoDrop). From 5 ng to 10 ng of cDNA was used to perform quantitative PCR (qPCR) using SYBR Select Master Mix (4472908, Thermo Fisher Scientific). All the qPCR amplifications were performed in the Step One Plus system (Applied Biosystems).

Gene expression values were calculated by the ΔCq method, using *GAPDH* as the housekeeping gene, and resulting experimental target values were normalized to the global mean of the control group. Normalized fold change was plotted using GraphPad Prism software. The sequences of the primers used in this study are listed in Supplementary Table 15.

### In vitro studies and viability assays

Approximately 2,500 cells were plated in triplicates in 96-well plates for 5 days. For in vitro experiments, cells were treated with 50 nM GSK–LSD1 (SML1072, Sigma-Aldrich), 50 nM bomedemstat (IMG-7289; HY-109169B, MedChem Express), 500 nM TAK-418 (HY-138830, MedChem Express), 100 nM LY2090314 (HY-16294, MedChem Express) and 100 nM elraglusib (9-ING-41; HY-113914, MedChem Express).

Cell viability was determined using a Cell Titer-Glo luminescent cell viability assay (G7572, Promega). Data were presented as proliferation present by comparing the treated groups with the vehicle-treated cells.

### Colony-forming unit assay

#### Leukaemia cell lines

Approximately 1,000 cells (for human leukaemia cell lines) and 500 cells (for mouse leukaemia cell lines) were initially plated in triplicates in the methylcellulose medium (MethoCult GF, H4435, StemCell Technologies) pre-added with vehicle, GSK-LSD1, LY2090314 or a combination of inhibitors. For serial replating, cells isolated from colonies in the previous plating were seeded again in the same semi-solid medium. Colony-forming units were scored every 7–10 days post-seeding.

#### Human CD34^+^ umbilical cord blood cells

Human cord blood CD34^+^ cells (200-0000, StemCell Technologies) were plated in methylcellulose (MethoCult H4534 Classic, StemCell Technologies). For each condition 5,000 cells were plated in 35-mm dishes in the presence of inhibitors. After 14 days, haematopoietic colonies were scored.

#### Human primary AML samples

Patient samples were thawed and cultured in StemSpan SFEM II (StemCell Technologies) supplemented with human recombinant Flt3/Flk-2, human recombinant IL-3, human recombinant GM-CSF, human recombinant IL-6, human stem cell factor and human recombinant G-CSF (StemCell Technologies) for 24 h. The cells were then treated with DMSO or inhibitors in methylcellulose medium (MethoCult H4535 or MethoCult H4534 Classic, StemCell Technologies) plated at 25,000 cells per millilitre on 35-mm culture dish and cultured for 7–10 days to form colonies.

### Wright–Giemsa staining

The cells collected from culture plates were spun onto a cytological slide by using a cytospin centrifuge (Cytospin 4 Cytocentrifuge). Then, slides were stained using the May–Grünwald–Giemsa staining method. The fixed cells were stained for 8 min in May–Grünwald stain (MG500, Sigma-Aldrich), then slides were sequentially washed 6 times in deionized water and then incubated for 30 min with Giemsa stain (1092041000, Sigma-Aldrich) and diluted with 19 volumes of distilled water. After this step, the cytological slides were rinsed again three times in distilled water and air dried. For long-time storage, a coverslip was attached to the slides by Eukitt mounting medium, which is an adhesive and specimen preservative that can be used manually and in automated coverslipping equipment. The slides were scanned using the NanoZoomer S210 slide scanner.

### TCF/LEF luciferase reporter assay

HCT116 and THP-1 cells were plated at 100,000 cells per well in 12-well plates, and following a 24-h incubation were infected with 30 µl TCF/LEF luciferase reporter lentivirus (BPS Biosciences). Following 48 h, cells were replated and selected for 3 days using puromycin. Then, cells treated with inhibitors in the presence and absence of WNT3A (40 ng ml^−1^) for 5 days. TCF/LEF activity was assessed using ONE-Step Luciferase reagent per recommended protocol (BPS Biosciences).

### RNA-seq protocol and analysis

For freshly cultured cells, total RNA isolation and DNaseI treatment were performed using the Direct-zol (TM) RNA MiniPrep kit (R2053, Zymo Research) as per the manufacturer’s protocol. Library preparation was conducted using the NEBNext UltraII RNA library kit (E7770S/L, NEB). Sequencing was conducted on an Illumina NextSeq 2000.

For ER-HOXA9 cell RNA-seq analysis, Fastq reads were aligned to the mouse genome (mm10) using STAR 2.7.0f. Read counts were mapped to genes using featureCounts. Differential analysis of gene expression was performed using DESeq2 (1.34.0). Only genes with more than 10 total counts when summed across all samples were considered. For THP-1 RNA-seq analysis, RNA-seq analysis was conducted using the EdgeR (3.50.3) limma (3.36.0) workflow. Reads were quantified using featureCounts, creating the raw gene count matrix. Data-quality metrics were investigated, and the limma voom normalization was applied to obtain counts per million (CPM) normalization and trimmed means of M values and normalization to finalize the differential expression analysis. The normalization accounted for sequencing depth. The log-CPM values were calculated, and adjusted *P* < 0.01 were considered significant. Genes responding synergistically to combo treatment were defined as those with adjusted *P* < 0.01 and fold change > 3 in combo versus vehicle and were also not significant at adjusted *P* < 0.01 and fold change > 1.5 in either GSK–LSD1 or LY2090314 versus vehicle. In THP-1 cells, genes responding synergistically to combo treatment were defined as those with adjusted *P* < 0.01 and more than 3× change in combo versus vehicle and not significant at *P* < 0.05 (non-adjusted) in either GSK–LSD1 or LY2090314 versus vehicle in THP-1 cells. Heatmaps of synergy genes and other gene lists were generated using Heatmapper.ca using Pearson correlation and average linkage settings. For pathway-level analysis, gene lists were either submitted to EnrichR^72–74^ or GSEA^75,76^ (4.3.3) was used. For GSEA, CPM-normalized data were used as inputs and GSEA MSigDB (2024.1) gene set compendia, or manually curated gene sets were used for enrichment using genes for permutations and default settings. Manually curated gene sets not in the MSigDB database included the Sykes terminal differentiation gene sets^24^, the LSC47 leukaemia stem cell signature^77^ and the Somervaille leukemia stem cell signatures^78^.

### ATAC-seq protocol and analysis

ATAC-seq was performed as previously described^23^. In brief, cells were treated with DNAseI (EN0521, Life Tech) to remove genomic DNA contamination. Live cell samples were quantified and assessed for viability and after cell lysis and cytosol removal, nuclei were treated with Tn5 enzyme (20034197, Illumina) for 30 min at 37 °C and purified with the Minelute PCR Purification Kit (28004, Qiagen) to produce tagmented DNA samples. Tagmented DNA was barcoded with Nextera Index Kit v2 (FC-131-2001, Illumina) and amplified via PCR before an SPRI Bead cleanup to yield purified DNA libraries. Sequencing was performed on an Illumina HiSeq instrument (4000 or equivalent). Fastq files were subjected to quality control with FastQC (0.11.9) and then trimmed with Cutadapt (2.1) with reads less than 20 nucleotides being filtered out. Reads were then mapped against mm10 with Bowtie2 (2.4.4), and duplicate reads were removed with samtools (1.15.1) rmdup, and bam files were converted to bed files with bedtools (2.30.0) bamtobed. Peaks were then called with MACS2 (2.2.7.1) with replicates being merged for downstream analyses. For heatmaps and PCAs, matrices were generated with deeptools (3.5.1) computeMatrix, and heatmaps and PCAs were generated with deeptools plotHeatmap and ggplot2 (3.4.2), respectively. IFNα signal profiles were generated with deeptools plotHeatmap using the *IFNA* promoter regions as an input. *IFNA* promoters were extracted using the ChIPseeker (1.42.0) R (4.3.0) package. ATAC-seq tracks were visualized with Integrative Genomics Viewer (2.16.2). Homer (5.1) was used for motif enrichment analyses using default settings. All operations were performed using default settings unless otherwise noted.

### CUT&RUN protocol and analysis

In brief, 250,000 cells were washed in 1 ml of wash buffer (20 mM HEPES pH 7.5, 0.5 mM spermidine and 120 mM NaCl) and centrifuged at 600*g* at room temperature three times, and the pellet were resuspended in 100 μl of wash buffer per reaction. Following this, 10 μl activated concavalin A beads per reaction was added, and the mixture was incubated at room temperature for 10 min with intermittent shaking. Captured cells were resuspended in 50 μl of antibody-binding buffer (wash buffer with digitonin 0.05% and EDTA). Primary antibodies, including negative and positive controls, were added, and the tubes were nutated overnight at 4 °C. On the following day, the samples were washed twice in dig wash buffer (wash buffer with 0.05% digitonin), and a master mix of pAG-MNase was prepared and added to each sample. After nutating at 4 °C for 1 h, the samples were washed to remove unbound pAG-MNase. Tubes were cooled to 0 °C for 5 min, then supplemented with CaCl_2_ to promote MNase digestion at 4 °C for 2 h followed by the addition of 2X STOP buffer (340 mM NaCl, 20 mM EDTA, 4 mM EGTA and 0.05% digitonin) and incubation at 37 °C for 10–15 min. Following a brief spin and magnetic separation, the supernatant containing enriched target-bound chromatin was proceeded directly with DNA cleanup with the Zymo DNA Clean&Concentrator-5 kit (Zymo Research). DNA libraries were prepared by the NEBNext Ultra II DNA library kit (E7645S) as per the manufacturer’s instructions.

CUT&RUN samples were processed via Nextflow (21.10.6), using the nf-core CUT&RUN pipeline (v3.0.0)^79^. Samples were aligned to the hg38 reference genome. Adapters were trimmed using Trim Galore (0.6.6), and paired-end alignment was performed using Bowtie2 (2.4.4). Mapping rates, GC content and other sample quality metrics were derived from nf-core via MultiQC. Peak calling was finalized using SEACR^80^ (1.3) with a standard peak threshold of 0.05 and spike-in calibration performed with the *Saccharomyces cerevisiae* genome. Heatmap and PCA analyses by gene and peak were performed using deepTools (3.5.1). Downstream peak-based analyses were done with peak bed files from replicate experiments being merged. Merging was done using bedTools (2.30.0) concatenate to combine peak files, bedTools sort to order peaks, and bedTools merge to merge peak regions. Motif enrichment analysis, track visualization and signal over gene set promoter regions (IFNα and Sykes myeloid differentiation top 200 gene promoter regions) were done as described in the ATAC-seq analysis section in the Methods. Genomic distribution analyses were done with the ChIPseeker (1.42.0) R (4.3.0) package, and peak and gene overlaps were quantified with bedTools intersect. The sequences of the primers used for CUT&RUN-qPCR are listed in Supplementary Table 16.

### Clinical dataset survival analysis

Patient data used in this study were taken from the cohort used in Bottomly et al.^81^ and referred to as the OHSU patient dataset. For signature score analyses, patients were scored according to their match in expression to a list of signature genes (upregulated genes only, downregulated genes only or both upregulated and downregulated genes) using the singscore (1.26.0) R (4.3.0) package^82^. If upregulated signature genes only were used, the patient score is high if the patient upregulates those genes. If downregulated signature genes only were used, the patient score is high if the patient downregulates those genes. If both upregulated and downregulated signature genes were used, the upregulated gene score and downregulated gene scores are combined. Pearson correlation was used to quantify correlations between signature scores among the patients. Components of signatures used for score correlations in Fig. 5j were: upregulated genes of the ER-HOXA9 synergy signature and downregulated genes of the Somervaille LSC signature (upper left); downregulated genes of the ER-HOXA9 synergy signature and total score of the MSigDB Wnt Signaling signature (upper right); upregulated and downregulated genes of the ER-HOXA9 synergy signature and upregulated genes of the MSigDB Hallmark Interferon Alpha Response signature (lower left); and upregulated genes of the MSigDB Hallmark Interferon Alpha Response signature and downregulated genes of the Somervaille LSC signature (lower right). For survival analyses, Kaplan–Meier plotting was performed using the ggsurvplot function of the survminer R (4.3.0) package. Patients were stratified by median synergy score enrichment using the downregulated genes of the ER-HOXA9 synergy signature. Statistical significance of survival data was tested with log-rank tests using the survdiff function of the Survival (3.8-3) R (4.3.0) package.

### Immunoblotting

Cells were lysed in a lysis buffer (0.1% SDS, 400 mM NaCl, 1 mM EDTA, 50 mM Tris-HCl and 1% Triton) plus protease inhibitor cocktail (11836170001, Sigma-Aldrich). Protein quantification was performed using a BCA assay (Promega). Of proteins, 20–40 μg was mixed with Laemmli (Bio-Rad) and denatured for 10 min at 95 °C. Cell lysates were loaded onto each lane of a 4–20% Mini-PROTEAN TGX Gel (Bio-Rad). The proteins were then transferred to a Trans-Blot Turbo Midi Nitrocellulose Transfer membrane (Bio-Rad). Following this, the membrane was blocked using 5% BSA and incubated with primary antibodies overnight at 4 °C. The next day, after three washes with 1% TBS-T (each wash 10 min), membrane was incubated with the proper secondary HRP antibodies, diluted in 5% BSA, for 30–60 min at room temperature. The membrane was washed again with 1% TBS-T three times, and ECL was applied for membrane development. The Bio-Rad ChemiDoc was used for the acquisition of western blot images. Antibodies included: β-catenin (D10A8) (dilution 1:1,000; 8480S, Cell Signaling), IRF-7 antibody (F-1; dilution 1:1,000; sc-74471, Santa Cruz Biotechnology), α-tubulin (DM1A; dilution 1:1,000; sc-32293, Santa Cruz Biotechnology), vinculin (42H89L44; dilution 1:1,000; 700062 Thermo Fisher Scientific), β-actin (13E5; dilution 1:1,000; 4970S, Cell Signaling), GSK3α (dilution 1:1,000; 9338, Cell Signaling), GSK3β (D5C5Z; dilution 1:1,000; 12456, Cell Signaling), anti-GSK3α and anti-GSK3β (phospho-Y216 + Y279) antibody (M132; dilution 1:1,000; ab45383, Abcam), STAT1 (dilution 1:1,000; 9172, Cell Signaling), phospho-STAT1 (Tyr701) monoclonal antibody (ST1P-11A5; dilution 1:1,000; 33-3400, Thermo Fisher Scientific), anti-rabbit IgG, HRP-linked antibody (dilution 1:5,000; 7074S, Cell Signaling) and anti-mouse IgG, HRP-linked antibody (dilution 1:5,000; 7076S, Cell Signaling).

### Immunoprecipitation

Of cleared protein lysate, 1.5–2 mg was used per immunoprecipitation (IP) in IP buffer (10 mM Tris-HCl pH 7.6, 150 mM NaCl and 0.2% NP-40) supplemented with Halt Protease and Phosphatase Inhibitor Cocktail (78445, Thermo Fisher Scientific). Of IP volume, 10% was designated for input assessments. Protein lysate was immunoprecipitated with 10 µg of antibody pre-bound to 30 µl of washed protein G Dynabeads (Invitrogen) per IP. IPs were conducted overnight at 4 °C, washed three times with IP buffer and once with the IP wash Buffer (10 mM Tris-HCl pH 7.6, 250 mM NaCl and 0.2% NP-40), both supplemented with Halt Protease and Phosphatase Inhibitor Cocktail (78445, Thermo Fisher Scientific). Then IPs were eluted in Laemmli buffer by boiling, then isolated from beads and transferred to new tubes for western blot analysis.

### Software and statistical analysis

All experiments were performed with at least three replicates, with the specific number of replicates stated in the figure legends. Unless otherwise stated, statistical analyses were performed using GraphPad prism (v10.3.0) using two-way ANOVA, and statistical significance was determined at a *P* < 0.05.

Related to Fig. 5a–c, note that in the boxplots, the middle line represents the median, the lower and upper hinges represent the 25th and 75th percentiles, respectively, the lower whisker extends from the lower hinge to the smallest value at most 1.5× the interquartile range of the hinge, and the upper whisker extends from the upper hinge to the largest value no further than 1.5× the interquartile range of the hinge. Data beyond the whiskers are outliers that are plotted individually.

### Reporting summary

Further information on research design is available in the Nature Portfolio Reporting Summary linked to this article.

## Online content

Any methods, additional references, Nature Portfolio reporting summaries, source data, extended data, supplementary information, acknowledgements, peer review information; details of author contributions and competing interests; and statements of data and code availability are available at 10.1038/s41586-025-08915-1.

## Supplementary information

Supplementary Information This file contains the raw immunoblot images relating to Extended Data Figures 3, 5 and 6, Supplementary Tables 13–16, and flow cytometry gating/sorting strategies.

Reporting Summary

Supplementary Table 1 RNAseq-DEGs.

Supplementary Table 2 Gene expression signatures used in study.

Supplementary Table 3 Combo synergy genes.

Supplementary Table 4 ER-HoxA9 ATAC-seq peaks-d1.

Supplementary Table 5 ER-HoxA9 ATAC-seq peaks-d3.

Supplementary Table 6 ER-HoxA9 ATAC-seq motif enrichments.

Supplementary Table 7 THP-1 β-catenin CUT&RUN peaks.

Supplementary Table 8 THP-1 IRF7 CUT&RUN peaks.

Supplementary Table 9 THP-1 IRF7 and β-catenin peak intersections.

Supplementary Table 10 THP-1 IRF7 and β-catenin CUT&RUN peak intersection motif enrichments.

Supplementary Table 11 THP-1 IRF7 and β-catenin peak intersection Hallmark gene set database enrichments.

Supplementary Table 12 Flow results GSK-LSD1 50 nM and LY2090314 100 nM in Primary samples.

## Source data

Source Data Fig. 2

Source Data Fig. 3

Source Data Fig. 5

Source Data Extended Data Fig. 3

Source Data Extended Data Fig. 9

## Data Availability

RNA-seq data for ER-HOXA9 cells treated with inhibitors have been deposited under GSE249879. ATAC-seq data for ER-HOXA9 cells treated with inhibitors have been deposited under GSE249773. CUT&RUN and RNA-seq data for THP-1 cells treated with inhibitors have been deposited under GSE251860. The OHSU clinical dataset of patients with AML^81^ was downloaded from cBioPortal (https://www.cbioportal.org/study/summary?id=aml_ohsu_2022). GSEAs utilized the Molecular Signatures Database (MSigDB) (https://www.gsea-msigdb.org/gsea/msigdb)^75,83,84^. Additional pathway enrichment analyses utilized the EnrichR database (https://maayanlab.cloud/Enrichr/)^72–74^. Source data are provided with this paper.

## References

[1] DiNardo, C. D., Erba, H. P., Freeman, S. D. & Wei, A. H. Acute myeloid leukaemia. *Lancet* **401**, 2073–2086 (2023). 37068505 10.1016/S0140-6736(23)00108-3

[2] Döhner, H. et al. Diagnosis and management of AML in adults: 2022 recommendations from an international expert panel on behalf of the ELN. *Blood* **140**, 1345–1377 (2022). 35797463 10.1182/blood.2022016867

[3] De Thé, H. Differentiation therapy revisited. *Nat. Rev. Cancer* **18**, 117–127 (2018). 29192213 10.1038/nrc.2017.103

[4] Liu, J. et al. Wnt/β-catenin signalling: function, biological mechanisms, and therapeutic opportunities. *Signal Transduct Target Ther.* **7**, 3 (2022). 34980884 10.1038/s41392-021-00762-6 PMC8724284

[5] Yi, M. et al. The global burden and attributable risk factor analysis of acute myeloid leukemia in 195 countries and territories from 1990 to 2017: estimates based on the global burden of disease study 2017. *J. Hematol. Oncol.* **13**, 72 (2020). 32513227 10.1186/s13045-020-00908-z PMC7282046

[6] Stahl, M. et al. Hypomethylating agents in relapsed and refractory AML: outcomes and their predictors in a large international patient cohort. *Blood Adv.* **2**, 923–932 (2018). 29685952 10.1182/bloodadvances.2018016121 PMC5916007

[7] Döhner, H., Weisdorf, D. J. & Bloomfield, C. D. Acute myeloid leukemia. *N. Engl. J. Med.* **373**, 1136–1152 (2015). 26376137 10.1056/NEJMra1406184

[8] Liu, T. X. et al. Gene expression networks underlying retinoic acid-induced differentiation of acute promyelocytic leukemia cells. *Blood* **96**, 1496–1504 (2000). 10942397

[9] Ablain, J. et al. Uncoupling RARA transcriptional activation and degradation clarifies the bases for APL response to therapies. *J. Exp. Med.* **210**, 647–653 (2013). 23509325 10.1084/jem.20122337 PMC3620357

[10] Zhang, X. W. et al. Arsenic trioxide controls the fate of the PML-RARα oncoprotein by directly binding PML. *Science* **328**, 240–243 (2010). 20378816 10.1126/science.1183424

[11] Ablain, J. et al. Activation of a promyelocytic leukemia-tumor protein 53 axis underlies acute promyelocytic leukemia cure. *Nat. Med.* **20**, 167–174 (2014). 24412926 10.1038/nm.3441

[12] De Thé, H., Pandolfi, P. P. & Chen, Z. Acute promyelocytic leukemia: a paradigm for oncoprotein-targeted cure. *Cancer Cell* **32**, 552–560 (2017). 29136503 10.1016/j.ccell.2017.10.002

[13] McKenzie, M. D. et al. Interconversion between tumorigenic and differentiated states in acute myeloid leukemia. *Cell Stem Cell* **25**, 258–272.e9 (2019). 31374198 10.1016/j.stem.2019.07.001

[14] Wang, F. et al. Targeted inhibition of mutant IDH2 in leukemia cells induces cellular differentiation. *Science* **340**, 622–626 (2013). 23558173 10.1126/science.1234769

[15] Issa, G. C. et al. The menin inhibitor revumenib in KMT2A-rearranged or NPM1-mutant leukaemia. *Nature* **615**, 920–924 (2023). 36922593 10.1038/s41586-023-05812-3 PMC10060155

[16] Dafflon, C. Complementary activities of DOT1L and menin inhibitors in MLL-rearranged leukemia. *Leukemia* **31**, 1269–1277 (2017). 27840424 10.1038/leu.2016.327

[17] Højfeldt, J. W., Agger, K. & Helin, K. Histone lysine demethylases as targets for anticancer therapy. *Nat. Rev. Drug Discov.* **12**, 917–930 (2013). 24232376 10.1038/nrd4154

[18] Shi, Y. et al. Histone demethylation mediated by the nuclear amine oxidase homolog LSD1. *Cell* **119**, 941–953 (2004). 15620353 10.1016/j.cell.2004.12.012

[19] Harris, W. J. et al. The histone demethylase KDM1A sustains the oncogenic potential of MLL-AF9 leukemia stem cells. *Cancer Cell* **21**, 473–487 (2012). 22464800 10.1016/j.ccr.2012.03.014

[20] Hosseini, A. & Minucci, S. A comprehensive review of lysine-specific demethylase 1 and its roles in cancer. *Epigenomics* **9**, 1123–1142 (2017). 28699367 10.2217/epi-2017-0022

[21] Fang, Y., Liao, G. & Yu, B. LSD1/KDM1A inhibitors in clinical trials: advances and prospects. *J. Hematol. Oncol.* **12**, 129 (2019). 31801559 10.1186/s13045-019-0811-9 PMC6894138

[22] Roboz, G. J. et al. Phase I trials of the lysine-specific demethylase 1 inhibitor, GSK2879552, as mono- and combination-therapy in relapsed/refractory acute myeloid leukemia or high-risk myelodysplastic syndromes. *Leuk. Lymphoma* **63**, 463–467 (2022). 34927529 10.1080/10428194.2021.2012667

[23] Zee, B. M. et al. Combined epigenetic and metabolic treatments overcome differentiation blockade in acute myeloid leukemia. *iScience* **24**, 102651 (2021). 34151238 10.1016/j.isci.2021.102651 PMC8192696

[24] Sykes, D. B. et al. Inhibition of dihydroorotate dehydrogenase overcomes differentiation blockade in acute myeloid leukemia. *Cell* **167**, 171–186.e15 (2016). 27641501 10.1016/j.cell.2016.08.057 PMC7360335

[25] Zhan, T., Rindtorff, N. & Boutros, M. Wnt signaling in cancer. *Oncogene* **36**, 1461–1473 (2017). 27617575 10.1038/onc.2016.304 PMC5357762

[26] Wang, Z. et al. Glycogen synthase kinase 3 in MLL leukaemia maintenance and targeted therapy. *Nature* **455**, 1205–1209 (2008). 18806775 10.1038/nature07284 PMC4084721

[27] Wang, Z. et al. GSK-3 promotes conditional association of CREB and its coactivators with MEIS1 to facilitate HOX-mediated transcription and oncogenesis. *Cancer Cell* **17**, 597–608 (2010). 20541704 10.1016/j.ccr.2010.04.024 PMC2919232

[28] McCubrey, J. A. et al. GSK-3 as potential target for therapeutic intervention in cancer. *Oncotarget* **5**, 2881–2911 (2014). 24931005 10.18632/oncotarget.2037 PMC4102778

[29] Rizzieri, D. A. et al. An open-label phase 2 study of glycogen synthase kinase-3 inhibitor LY2090314 in patients with acute leukemia. *Leuk. Lymphoma* **57**, 1800–1806 (2016). 26735141 10.3109/10428194.2015.1122781

[30] Hiatt, J. B. et al. Inhibition of LSD1 with bomedemstat sensitizes small cell lung cancer to immune checkpoint blockade and T-cell killing. *Clin. Cancer Res.* **28**, 4551–4564 (2022). 35920742 10.1158/1078-0432.CCR-22-1128 PMC9844673

[31] Baba, R. et al. LSD1 enzyme inhibitor TAK-418 unlocks aberrant epigenetic machinery and improves autism symptoms in neurodevelopmental disorder models. *Sci. Adv.* **7**, eaba1187 (2021). 33712455 10.1126/sciadv.aba1187 PMC7954450

[32] Maiques-Diaz, A. Enhancer activation by pharmacologic displacement of LSD1 from GFI1 induces differentiation in acute myeloid leukemia. *Cell Rep.* **22**, 3641–3659 (2018). 29590629 10.1016/j.celrep.2018.03.012 PMC5896174

[33] Hartung, E. E., Singh, K. & Berg, T. LSD1 inhibition modulates transcription factor networks in myeloid malignancies. *Front. Oncol.* **13**, 1149754 (2023). 36969082 10.3389/fonc.2023.1149754 PMC10036816

[34] Smitheman, K. N. et al. Lysine specific demethylase 1 inactivation enhances differentiation and promotes cytotoxic response when combined with all-*trans* retinoic acid in acute myeloid leukemia across subtypes. *Haematologica* **104**, 1156–1167 (2019). 30514804 10.3324/haematol.2018.199190 PMC6545850

[35] Hsu, A. et al. Clinical activity of 9-ING-41, a small molecule selective glycogen synthase kinase-3β (GSK-3β) inhibitor, in refractory adult T-cell leukemia/lymphoma. *Cancer Biol Ther.* **23**, 417–423 (2022). 35815408 10.1080/15384047.2022.2088984 PMC9272832

[36] Banerji, V. et al. The intersection of genetic and chemical genomic screens identifies GSK-3α as a target in human acute myeloid leukemia. *J. Clin. Invest.* **122**, 935–947 (2012). 22326953 10.1172/JCI46465 PMC3287215

[37] Wagner, F. F. et al. Exploiting an Asp-Glu “switch” in glycogen synthase kinase 3 to design paralog-selective inhibitors for use in acute myeloid leukemia. *Sci. Transl. Med.* **10**, eaam8460 (2018). 29515000 10.1126/scitranslmed.aam8460 PMC6553635

[38] Jefferies, C. A. Regulating IRFs in IFN driven disease. *Front. Immunol.* **10**, 325 (2019). 30984161 10.3389/fimmu.2019.00325 PMC6449421

[39] Sheng, W. et al. LSD1 ablation stimulates anti-tumor immunity and enables checkpoint blockade. *Cell* **174**, 549–563.e19 (2018). 29937226 10.1016/j.cell.2018.05.052 PMC6063761

[40] Kaidi, A., Williams, A. C. & Paraskeva, C. Interaction between β-catenin and HIF-1 promotes cellular adaptation to hypoxia. *Nat. Cell Biol.* **9**, 210–217 (2007). 17220880 10.1038/ncb1534

[41] Yang, P. et al. The cytosolic nucleic acid sensor LRRFIP1 mediates the production of type I interferon via a β-catenin-dependent pathway. *Nat. Immunol.* **11**, 487–494 (2010). 20453844 10.1038/ni.1876

[42] Kan, W. L. et al. Distinct assemblies of heterodimeric cytokine receptors govern stemness programs in leukemia. *Cancer Discov.* **13**, 1922–1947 (2023). 37191437 10.1158/2159-8290.CD-22-1396 PMC10401075

[43] Coccia, E. M. et al. STAT1 activation during monocyte to macrophage maturation: role of adhesion molecules. *Int. Immunol.* **11**, 1075–1083 (1999). 10383940 10.1093/intimm/11.7.1075

[44] Jerke, U. et al. Stat1 nuclear translocation by nucleolin upon monocyte differentiation. *PLoS ONE* **4**, e8302 (2009). 20011528 10.1371/journal.pone.0008302 PMC2788426

[45] Ostojic, A., Vrhovac, R. & Verstovsek, S. Ruxolitinib: a new JAK1/2 inhibitor that offers promising options for treatment of myelofibrosis. *Future Oncol.* **7**, 1035–1043 (2011). 21919691 10.2217/fon.11.81 PMC5147419

[46] Gupta, K. et al. GSK3 is a regulator of RAR-mediated differentiation. *Leukemia* **26**, 1277–1285 (2012). 22222598 10.1038/leu.2012.2 PMC4458365

[47] Cai, S. F. et al. Leukemia cell of origin influences apoptotic priming and sensitivity to LSD1 inhibition. *Cancer Discov.* **10**, 1500–1513 (2020). 32606137 10.1158/2159-8290.CD-19-1469 PMC7584353

[48] Scheller, M. et al. Hotspot DNMT3A mutations in clonal hematopoiesis and acute myeloid leukemia sensitize cells to azacytidine via viral mimicry response. *Nat. Cancer* **2**, 527–544 (2021). 35122024 10.1038/s43018-021-00213-9

[49] Liu, Y. et al. Small-molecule inhibition of the acyl-lysine reader ENL as a strategy against acute myeloid leukemia. *Cancer Discov.* **12**, 2684–2709 (2022). 36053276 10.1158/2159-8290.CD-21-1307 PMC9627135

[50] Vick, B. et al. An advanced preclinical mouse model for acute myeloid leukemia using patients’ cells of various genetic subgroups and in vivo bioluminescence imaging. *PLoS ONE* **10**, e0120925 (2015). 25793878 10.1371/journal.pone.0120925 PMC4368518

[51] Ebinger, S. et al. Plasticity in growth behavior of patients’ acute myeloid leukemia stem cells growing in mice. *Haematologica* **105**, 2855–2860 (2020). 33256387 10.3324/haematol.2019.226282 PMC7716350

[52] Khan, A. K. et al. Human bone marrow organoids for disease modeling, discovery, and validation of therapeutic targets in hematologic malignancies. *Cancer Discov.* **13**, 364–385 (2023). 36351055 10.1158/2159-8290.CD-22-0199 PMC9900323

[53] Olijnik, A. A. et al. Generating human bone marrow organoids for disease modeling and drug discovery. *Nat. Protoc.* **19**, 2117–2146 (2024). 38532070 10.1038/s41596-024-00971-7

[54] Roulois, D. et al. DNA-demethylating agents target colorectal cancer cells by inducing viral mimicry by endogenous transcripts. *Cell* **162**, 961–973 (2015). 26317465 10.1016/j.cell.2015.07.056 PMC4843502

[55] Mehdipour, P. et al. Epigenetic therapy induces transcription of inverted SINEs and ADAR1 dependency. *Nature* **588**, 169–173 (2020). 33087935 10.1038/s41586-020-2844-1

[56] Hosseini, A. et al. Retroelement decay by the exonuclease XRN1 is a viral mimicry dependency in cancer. *Cell Rep.* **43**, 113684 (2024). 38261511 10.1016/j.celrep.2024.113684 PMC11724374

[57] Zhang, Y. & Wang, X. Targeting the Wnt/β-catenin signaling pathway in cancer. *J. Hematol. Oncol.* **13**, 165 (2020). 33276800 10.1186/s13045-020-00990-3 PMC7716495

[58] Tsuchiya, K. et al. Development of a penetratin-conjugated stapled peptide that inhibits Wnt/β-catenin signaling. *Bioorg. Med. Chem.* **73**, 117021 (2022). 36198218 10.1016/j.bmc.2022.117021

[59] Holmes, T. et al. Glycogen synthase kinase-3β inhibition preserves hematopoietic stem cell activity and inhibits leukemic cell growth. *Stem Cells* **26**, 1288–1297 (2008). 18323411 10.1634/stemcells.2007-0600

[60] Wang, Y. et al. The Wnt/β-catenin pathway is required for the development of leukemia stem cells in AML. *Science* **327**, 1650–1653 (2010). 20339075 10.1126/science.1186624 PMC3084586

[61] Yeung, J. et al. β-Catenin mediates the establishment and drug resistance of MLL leukemic stem cells. *Cancer Cell* **18**, 606–618 (2010). 21156284 10.1016/j.ccr.2010.10.032

[62] Gandillet, A. et al. Heterogeneous sensitivity of human acute myeloid leukemia to β-catenin down-modulation. *Leukemia* **25**, 770–780 (2011). 21339756 10.1038/leu.2011.17 PMC4289854

[63] Guezguez, B. et al. GSK3 deficiencies in hematopoietic stem cells initiate pre-neoplastic state that is predictive of clinical outcomes of human acute leukemia. *Cancer Cell* **29**, 61–74 (2016). 26766591 10.1016/j.ccell.2015.11.012

[64] Lee, G. et al. Loss of GSK3β in hematopoietic stem cells results in normal hematopoiesis in mice. *Blood Adv.* **7**, 7185–7189 (2023). 37922427 10.1182/bloodadvances.2022008094 PMC10698258

[65] Parameswaran, R. et al. Repression of GSK3 restores NK cell cytotoxicity in AML patients. *Nat. Commun.* **7**, 11154 (2016). 27040177 10.1038/ncomms11154 PMC4822012

[66] Nan, Y. et al. Interferon independent non-canonical STAT activation and virus induced inflammation. *Viruses* **10**, 196 (2018). 29662014 10.3390/v10040196 PMC5923490

[67] Cheon, H. & Stark, G. R. Unphosphorylated STAT1 prolongs the expression of interferon-induced immune regulatory genes. *Proc. Natl Acad. Sci. USA* **106**, 9373–9378 (2009). 19478064 10.1073/pnas.0903487106 PMC2688000

[68] Najjar, I. & Fagard, R. STAT1 and pathogens, not a friendly relationship. *Biochimie* **92**, 425–444 (2010). 20159032 10.1016/j.biochi.2010.02.009 PMC7117016

[69] He, L. et al. Methods for high-throughput drug combination screening and synergy scoring. *Methods Mol. Biol.* **1711**, 351–398 (2018). 29344898 10.1007/978-1-4939-7493-1_17 PMC6383747

[70] Karjalainen, R. et al. JAK1/2 and BCL2 inhibitors synergize to counteract bone marrow stromal cell-induced protection of AML. *Blood* **130**, 789–802 (2017). 28619982 10.1182/blood-2016-02-699363

[71] Schreurs, R. R. C. E., Baumdick, M. E., Drewniak, A. & Bunders, M. J. In vitro co-culture of human intestinal organoids and lamina propria-derived CD4^+^ T cells. *STAR Protoc.* **2**, 100519 (2021). 34036282 10.1016/j.xpro.2021.100519 PMC8138864

[72] Chen, E. Y. et al. Enrichr: interactive and collaborative HTML5 gene list enrichment analysis tool. *BMC Bioinformatics* **14**, 128 (2013). 23586463 10.1186/1471-2105-14-128 PMC3637064

[73] Kuleshov, M. V. et al. Enrichr: a comprehensive gene set enrichment analysis web server 2016 update. *Nucleic Acids Res.* **44**, W90–W97 (2016). 27141961 10.1093/nar/gkw377 PMC4987924

[74] Xie, Z. et al. Gene set knowledge discovery with Enrichr. *Curr Protoc.* **1**, e90 (2021). 33780170 10.1002/cpz1.90 PMC8152575

[75] Subramanian, A. et al. Gene set enrichment analysis: a knowledge-based approach for interpreting genome-wide expression profiles. *Proc. Natl Acad. Sci. USA* **102**, 15545–15550 (2005). 16199517 10.1073/pnas.0506580102 PMC1239896

[76] Mootha, V. K. et al. PGC-1α-responsive genes involved in oxidative phosphorylation are coordinately downregulated in human diabetes. *Nat. Genet.* **34**, 267–273 (2003). 12808457 10.1038/ng1180

[77] Huang, B. J. et al. Integrated stem cell signature and cytomolecular risk determination in pediatric acute myeloid leukemia. *Nat. Commun.* **13**, 5487 (2022). 36123353 10.1038/s41467-022-33244-6 PMC9485122

[78] Somervaille, T. C. P. et al. Hierarchical maintenance of MLL myeloid leukemia stem cells employs a transcriptional program shared with embryonic rather than adult stem cells. *Cell Stem Cell* **4**, 129–140 (2009). 19200802 10.1016/j.stem.2008.11.015 PMC2670853

[79] Ewels, P. A. et al. The nf-core framework for community-curated bioinformatics pipelines. *Nat. Biotechnol.* **38**, 276–278 (2020). 32055031 10.1038/s41587-020-0439-x

[80] Meers, M. P., Tenenbaum, D. & Henikoff, S. Peak calling by sparse enrichment analysis for CUT&RUN chromatin profiling. *Epigenetics Chromatin* **12**, 42 (2019). 31300027 10.1186/s13072-019-0287-4 PMC6624997

[81] Bottomly, D. et al. Integrative analysis of drug response and clinical outcome in acute myeloid leukemia. *Cancer Cell* **40**, 850–864.e9 (2022). 35868306 10.1016/j.ccell.2022.07.002 PMC9378589

[82] Foroutan, M. et al. Single sample scoring of molecular phenotypes. *BMC Bioinformatics* **19**, 404 (2018). 30400809 10.1186/s12859-018-2435-4 PMC6219008

[83] Liberzon, A. et al. Molecular signatures database (MSigDB) 3.0. *Bioinformatics* **27**, 1739–1740 (2011). 21546393 10.1093/bioinformatics/btr260 PMC3106198

[84] Liberzon, A. et al. The Molecular Signatures Database (MSigDB) hallmark gene set collection. *Cell Syst.* **1**, 417–425 (2015). 26771021 10.1016/j.cels.2015.12.004 PMC4707969

