# Supplementary material for: Perturbing LSD1 and WNT rewires transcription to synergistically induce AML differentiation
Source: Nature. 2025 Apr 16;642(8067):508–18. doi: 10.1038/s41586-025-08915-1 (PMC12158781; doi:10.1038/s41586-025-08915-1)

nature portfolio

https://doi.org/10.1038/s41586-025-08915-1

Supplementary information

Perturbing LSD1 and WNT rewires transcription to synergistically induce AML differentiation

In the format provided by the authors and unedited

Raw immunoblot images related to Extended data Fig.3f

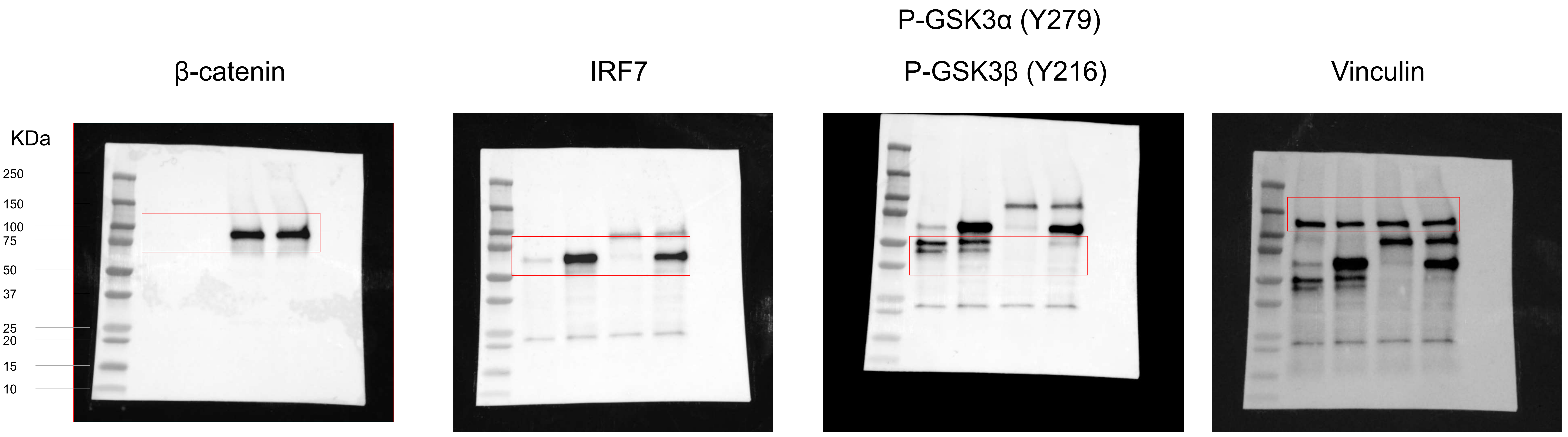

Raw immunoblot images related to Extended data Fig.5a

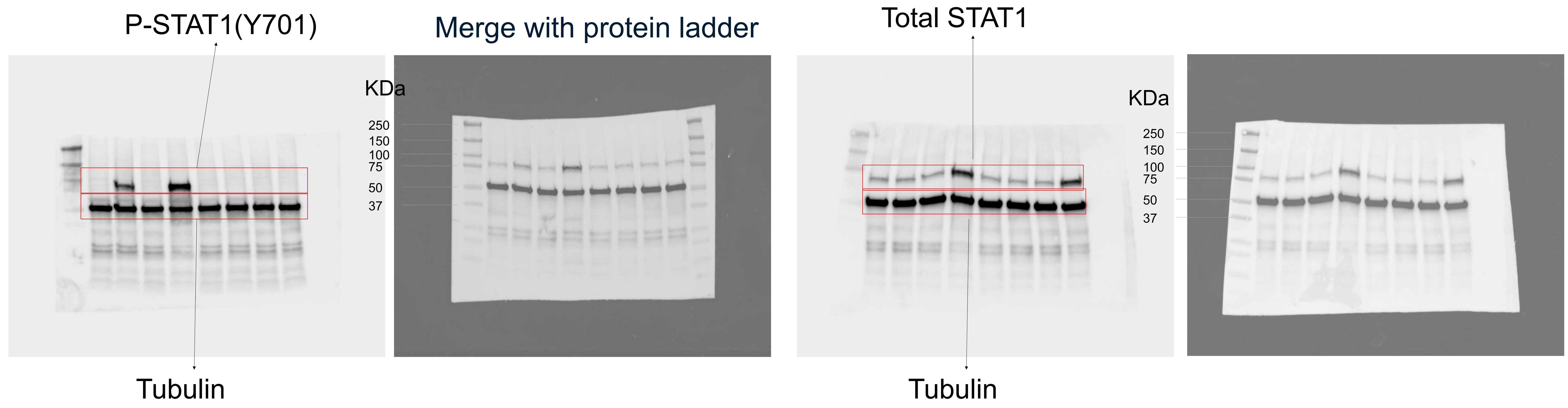

Raw immunoblot images related to Extended data Fig.5i

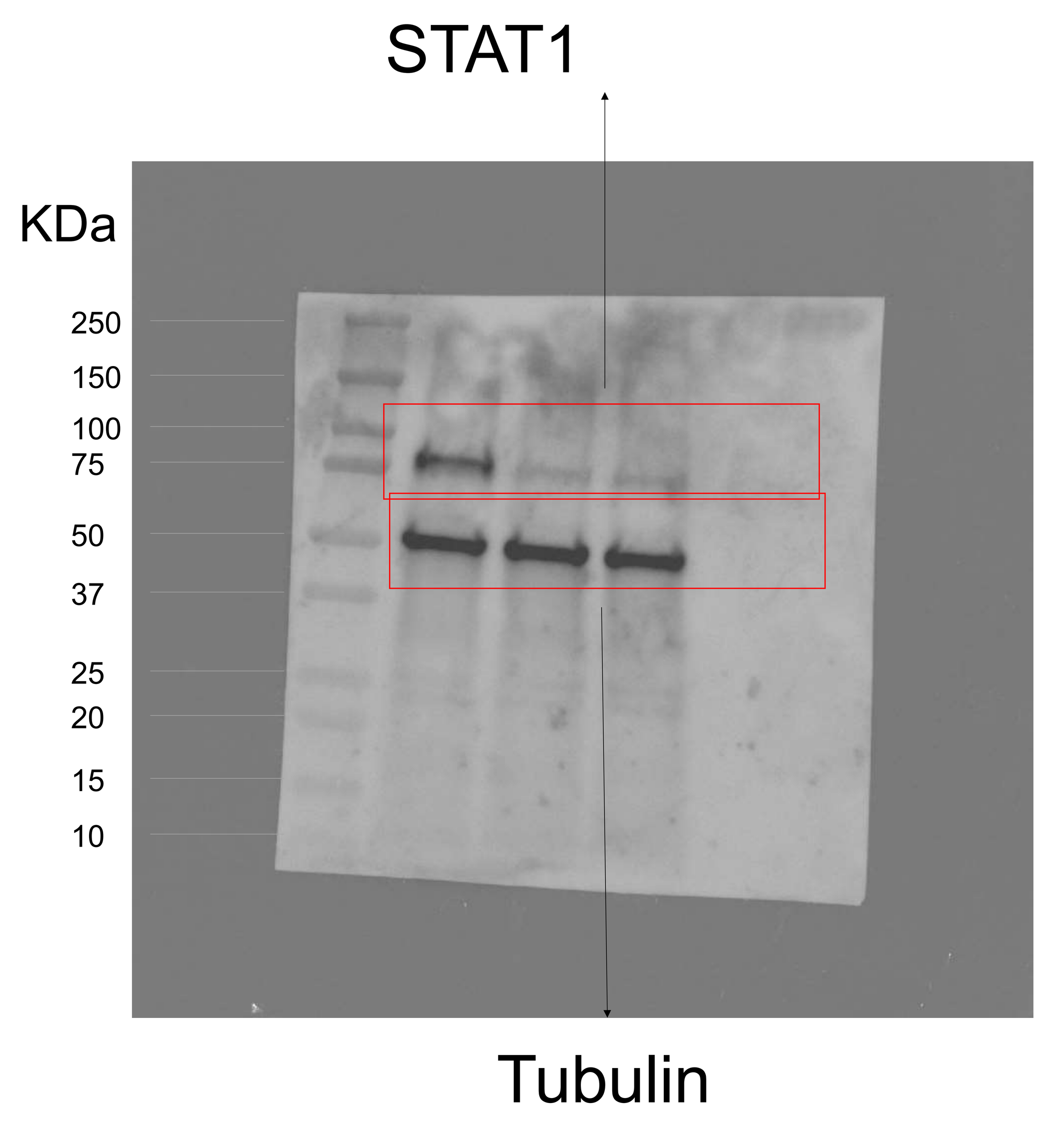

Raw immunoblot images related to Extended data Fig.5m

β-catenin

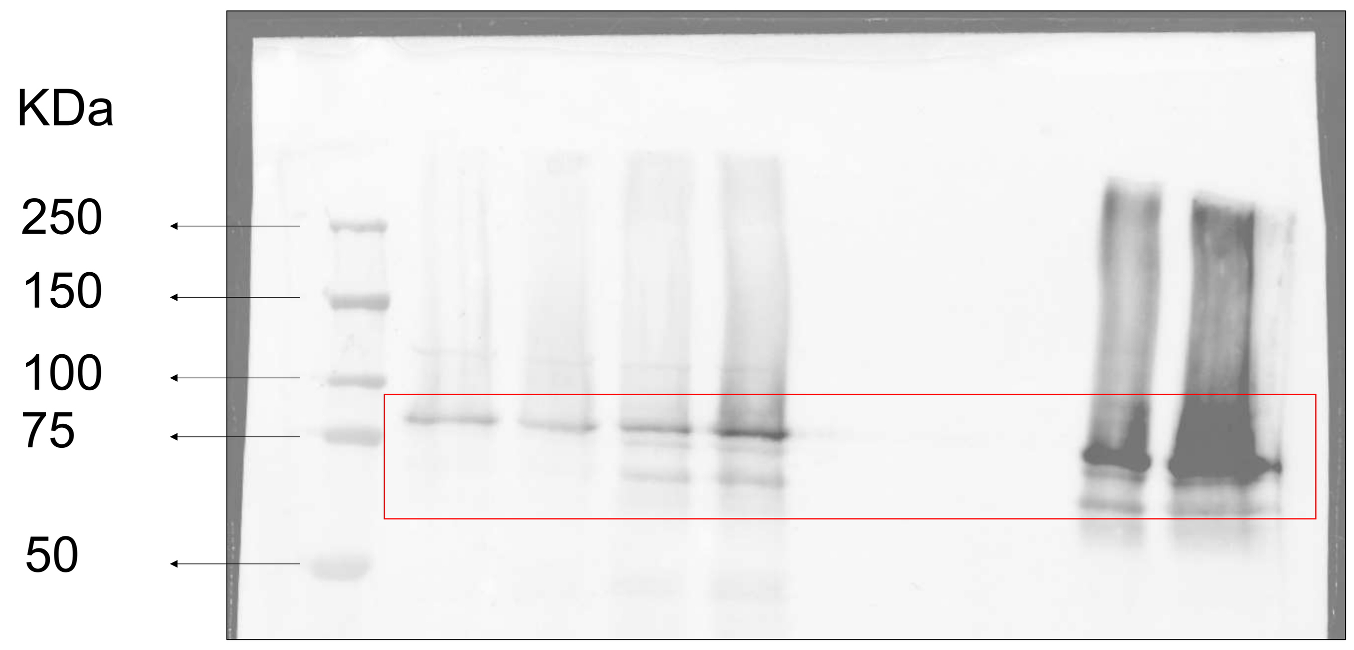

IRF7

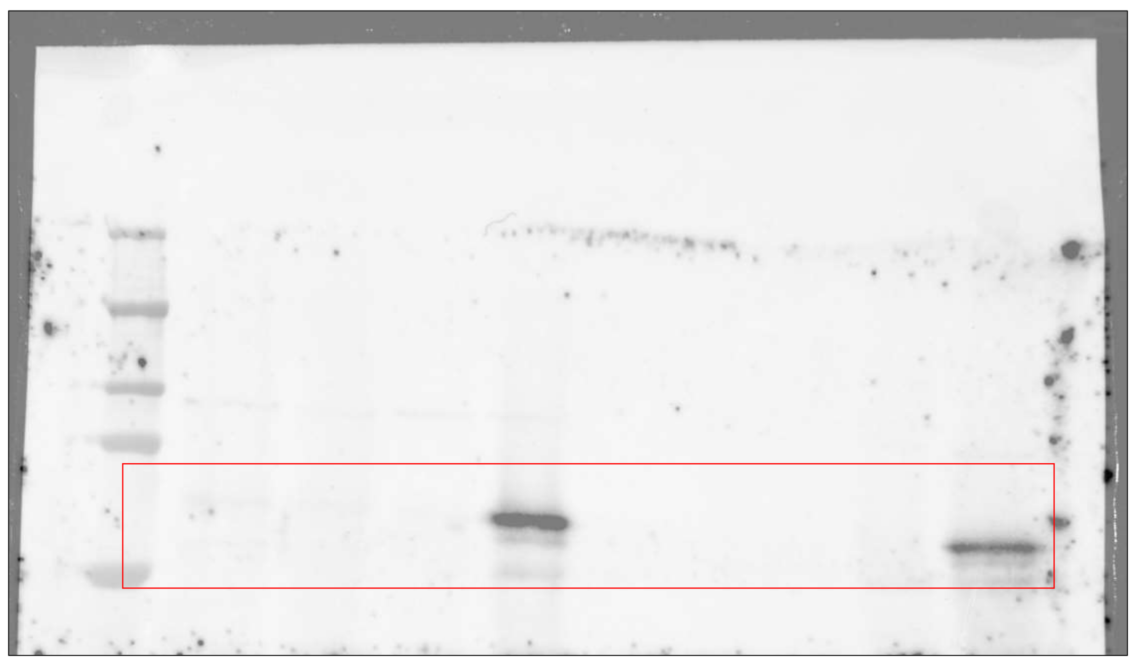

Vinculin

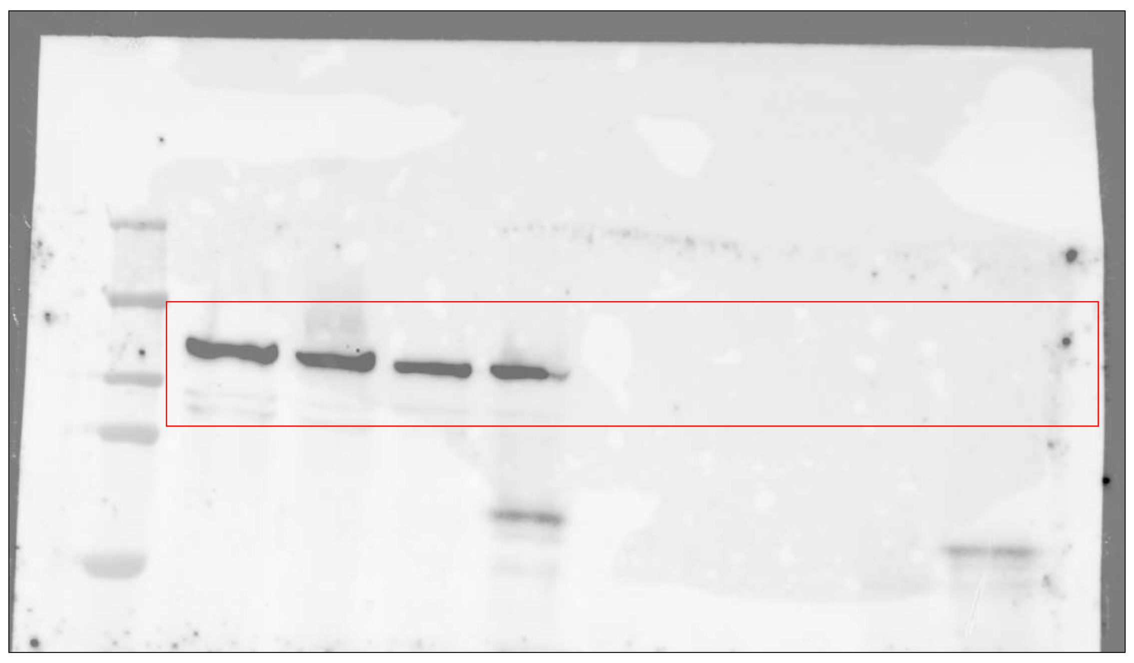

Raw immunoblot images related to Extended data Fig.6a

GSK3α
GSK3β

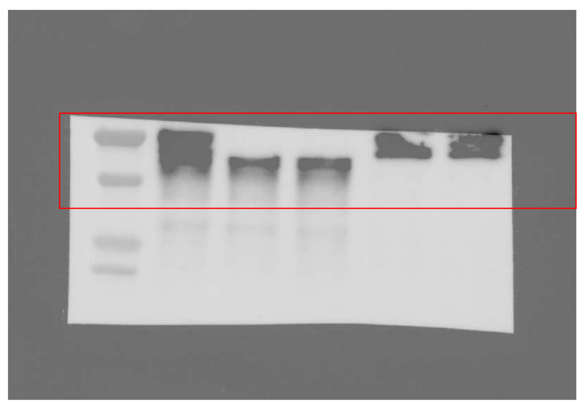

β-catenin

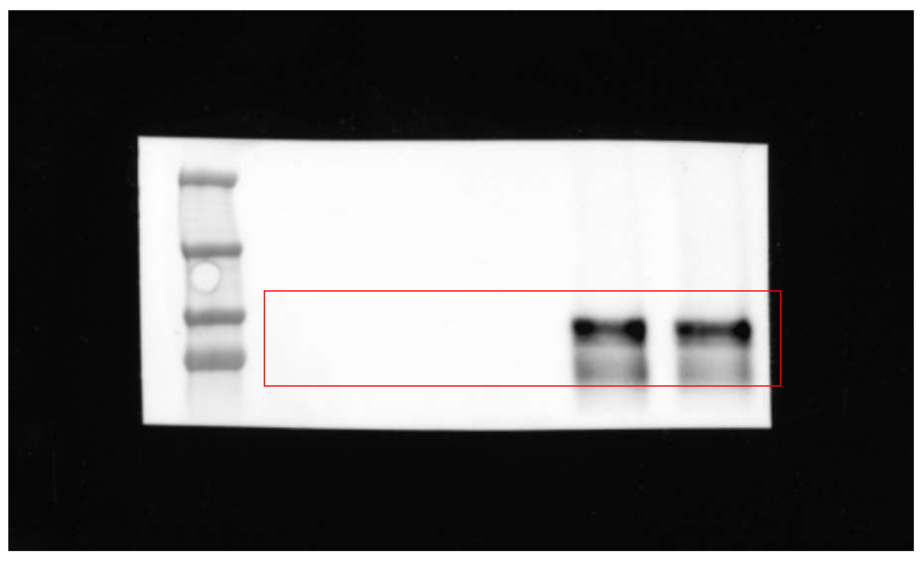

Merge with protein ladder

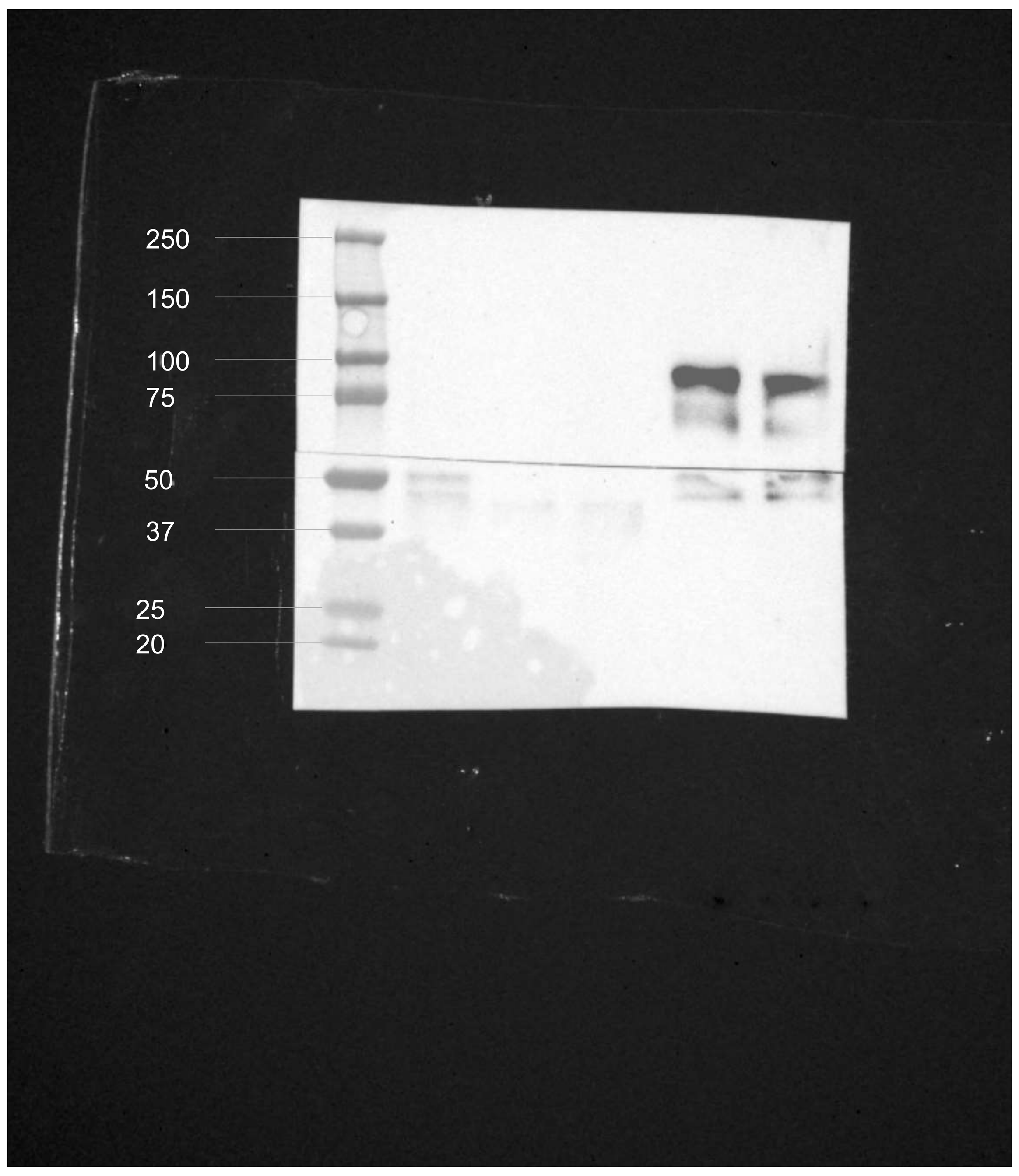

Vinculin

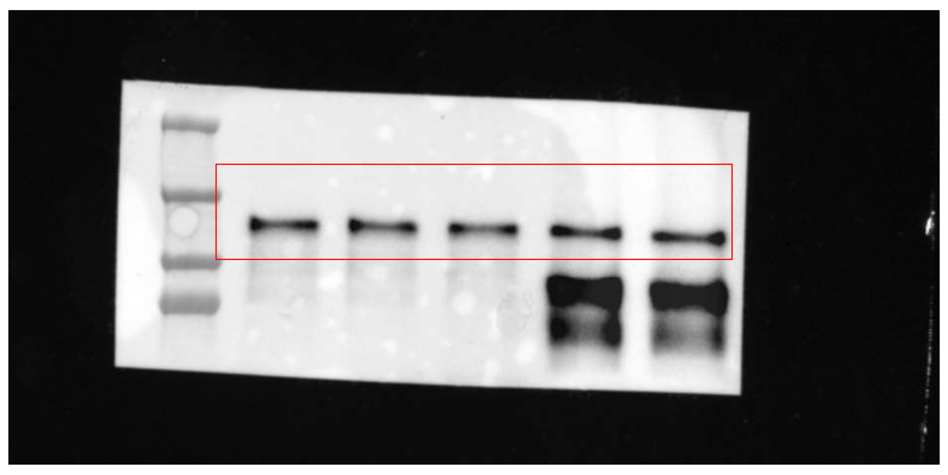

Raw immunoblot images related to Extended data Fig.6e

β-catenin

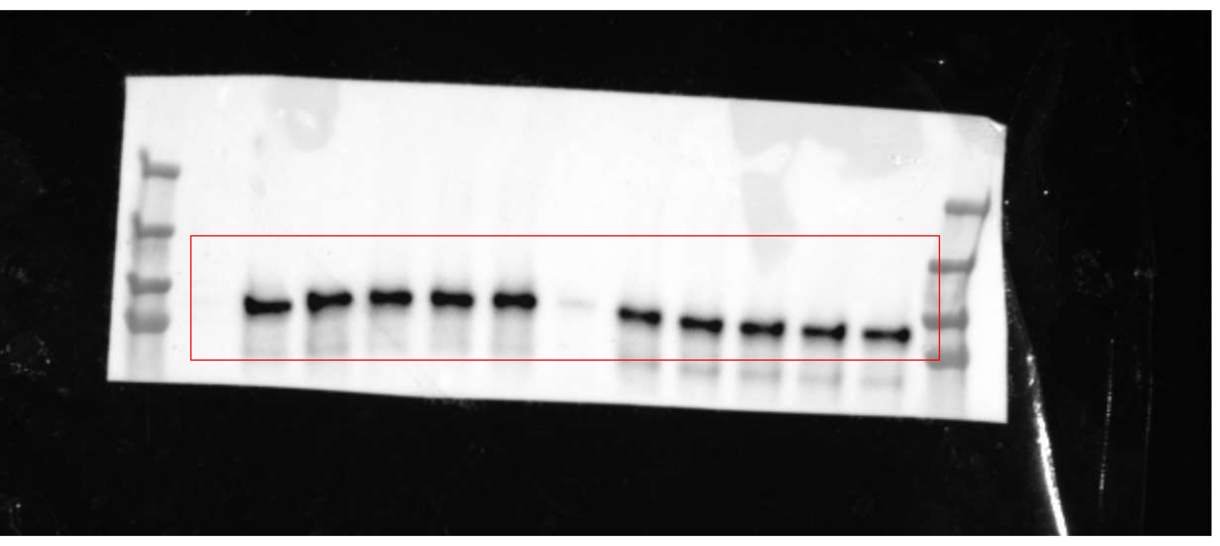

GSK3β

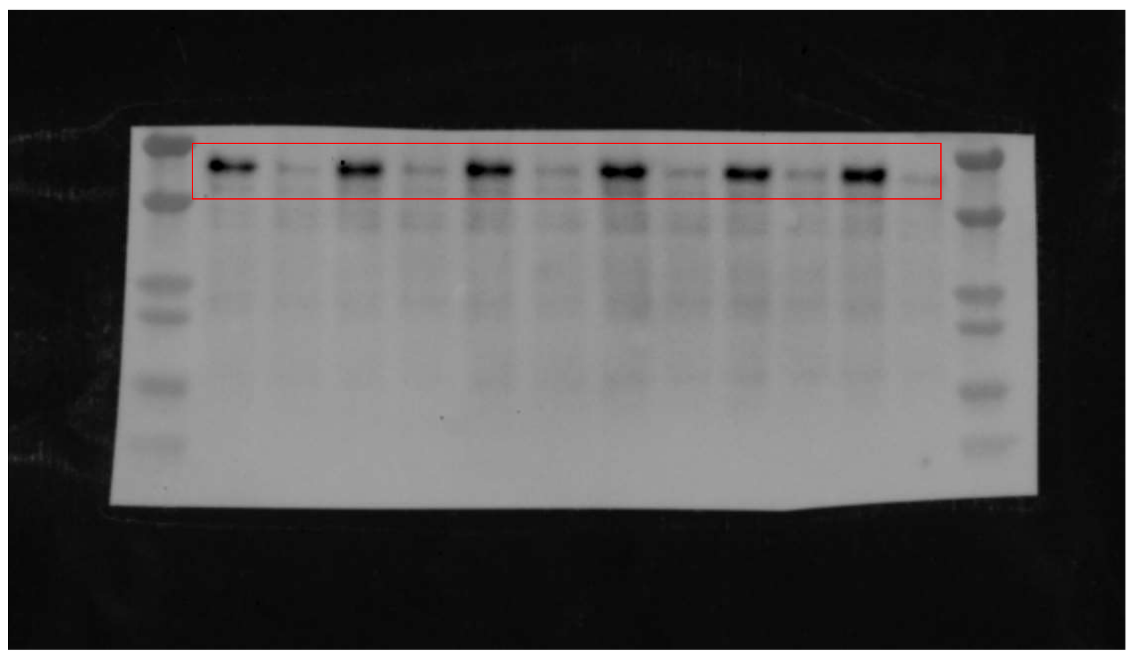

IRF7

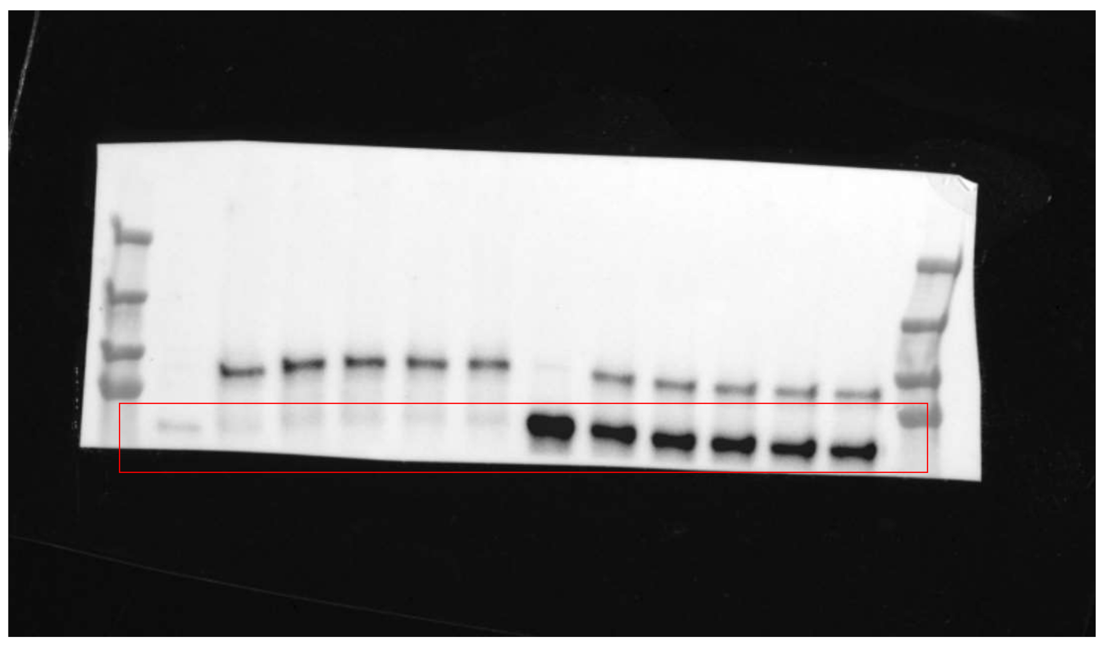

Vinculin

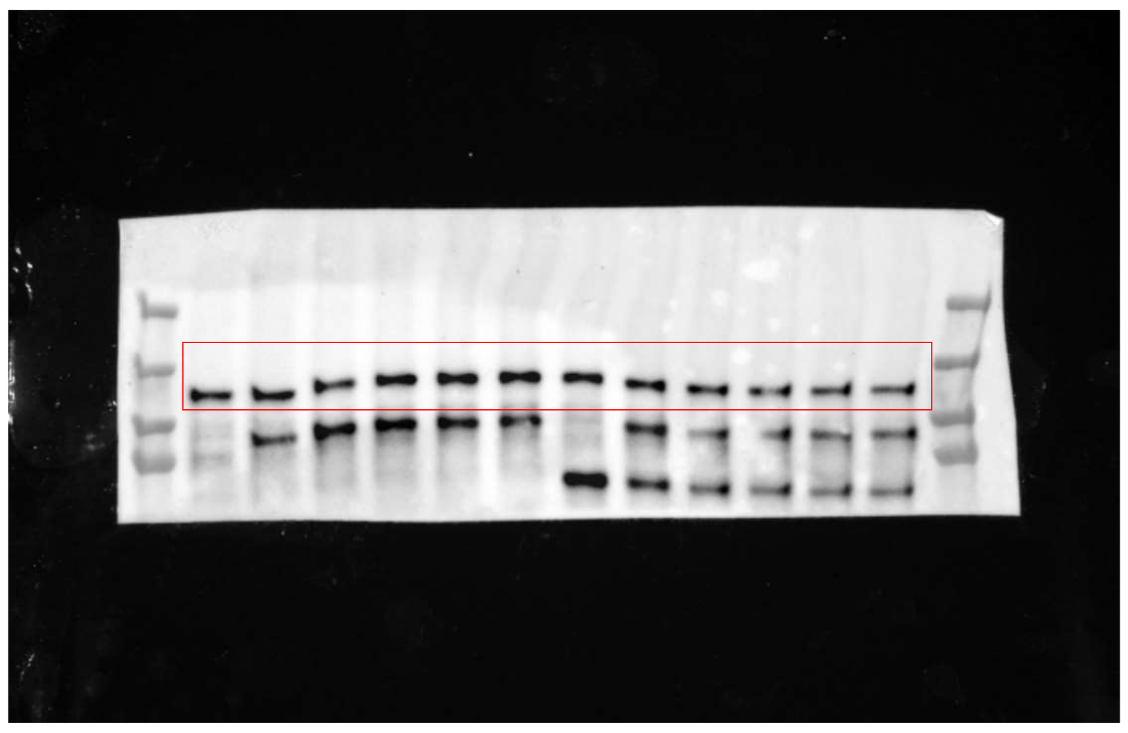

β -Actin

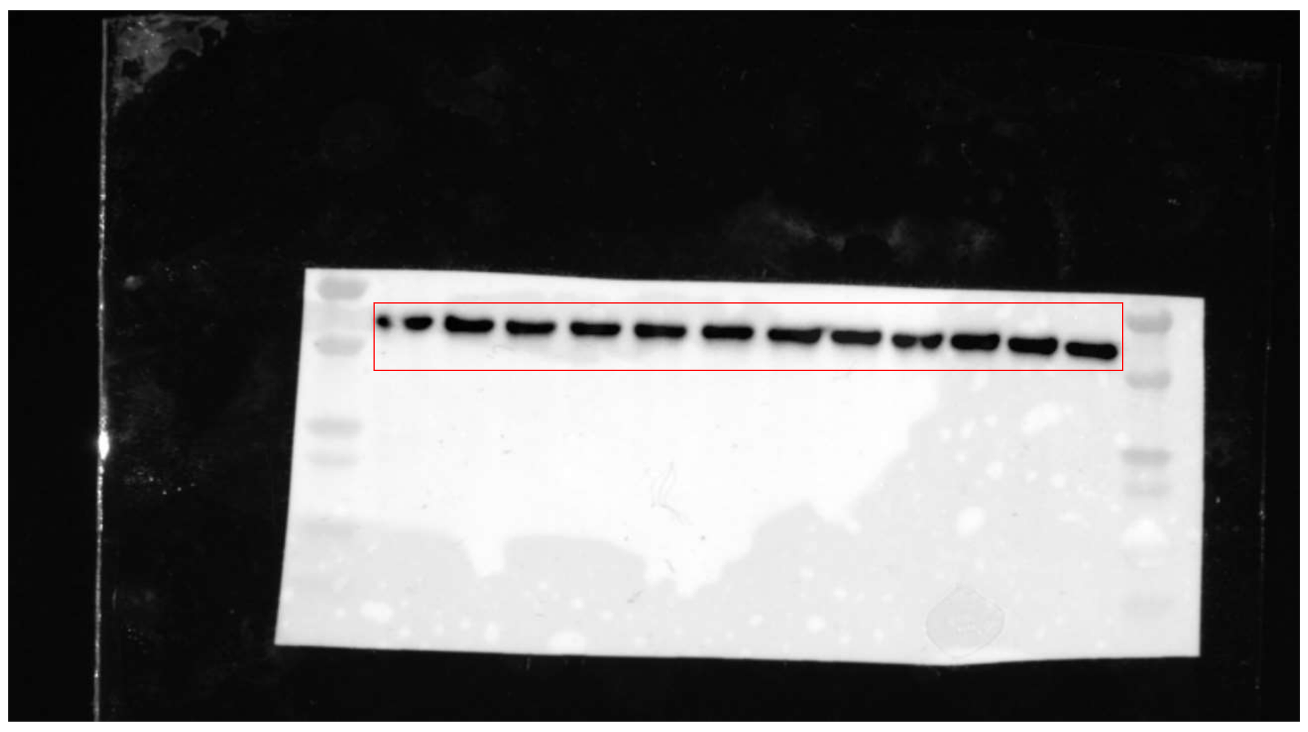

Merge with protein ladder

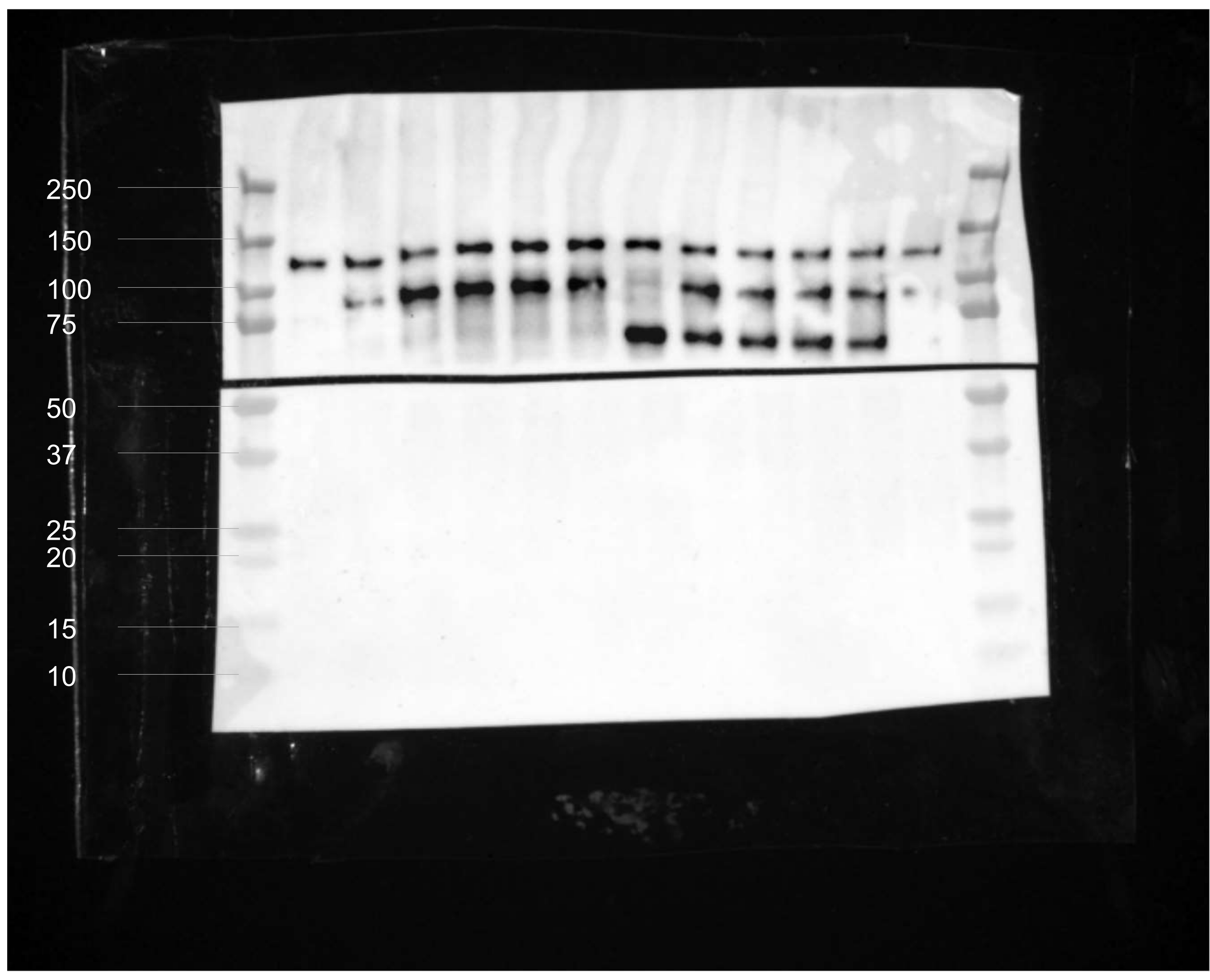

Raw immunoblot images related to Extended data Fig.6h

β-catenin

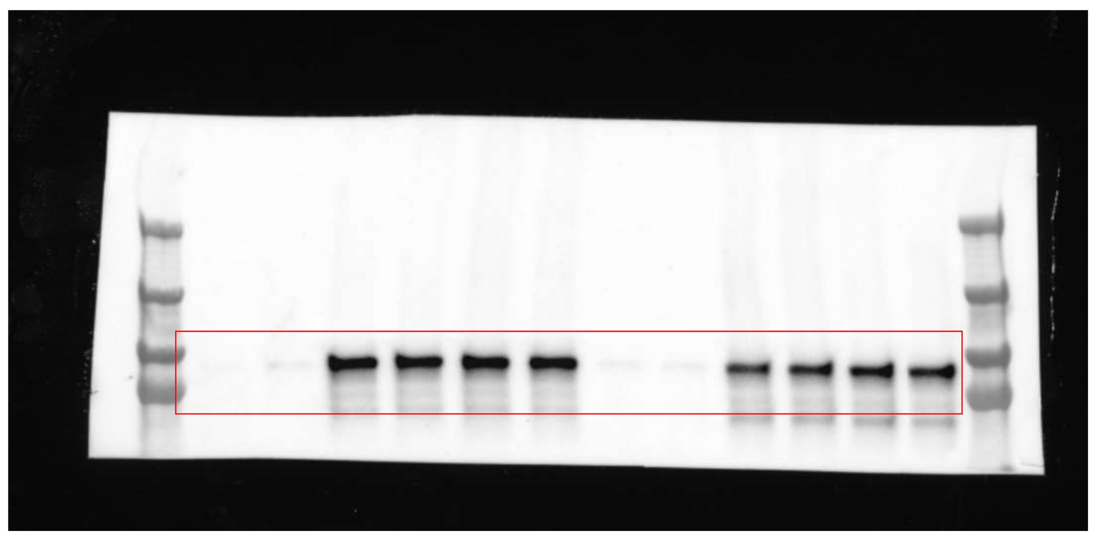

Vinculin

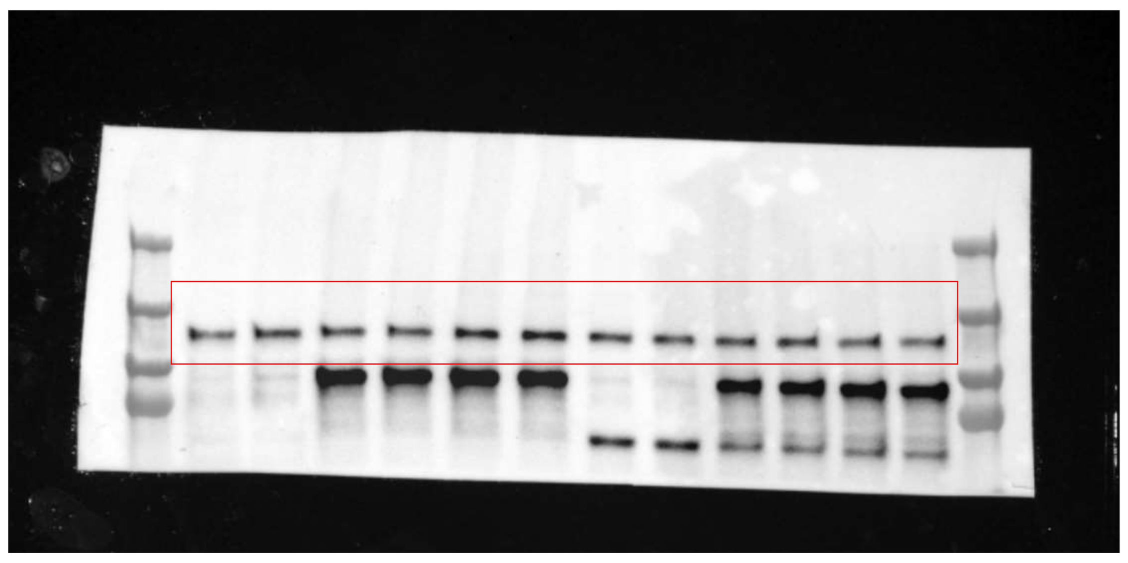

IRF7

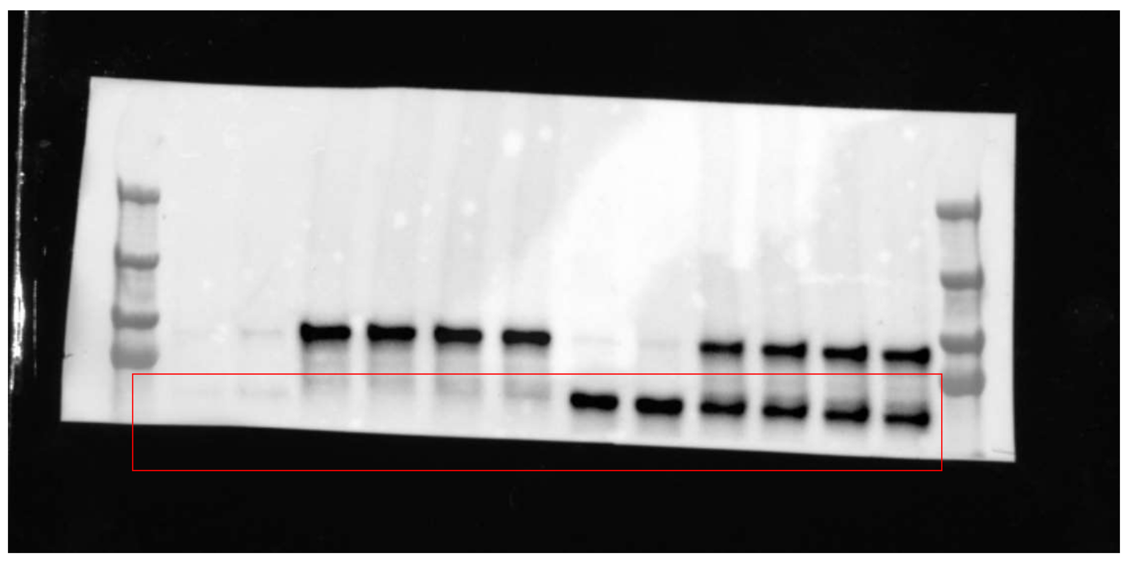

Merge with protein ladder

GSK3α
GSK3β

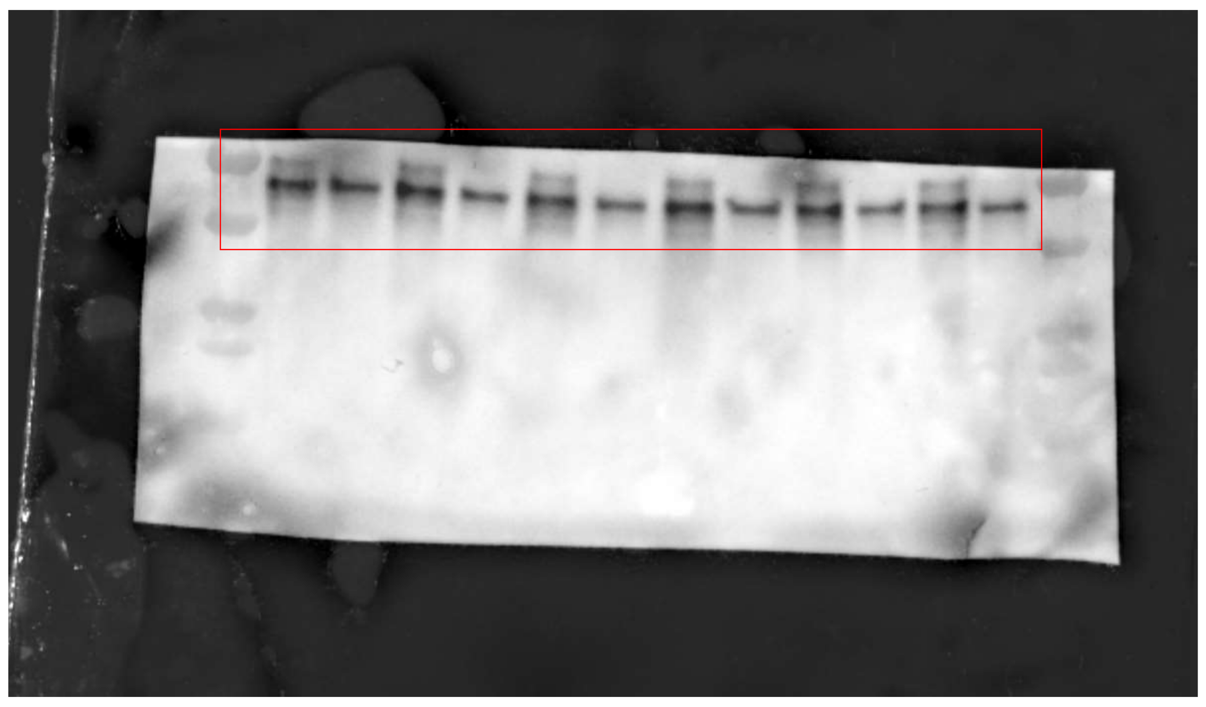

β -Actin

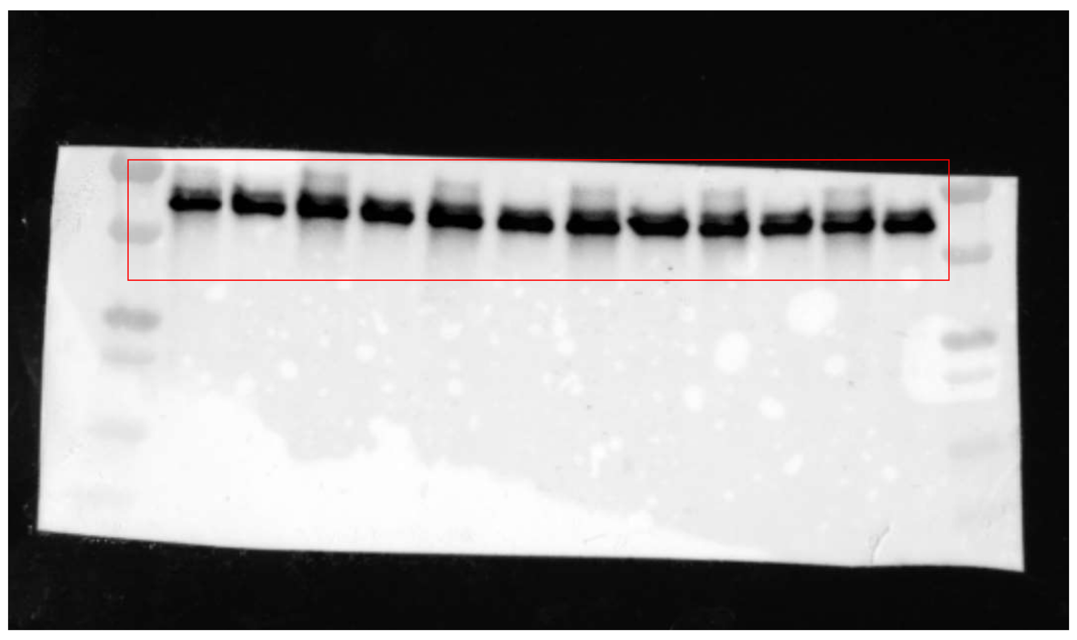

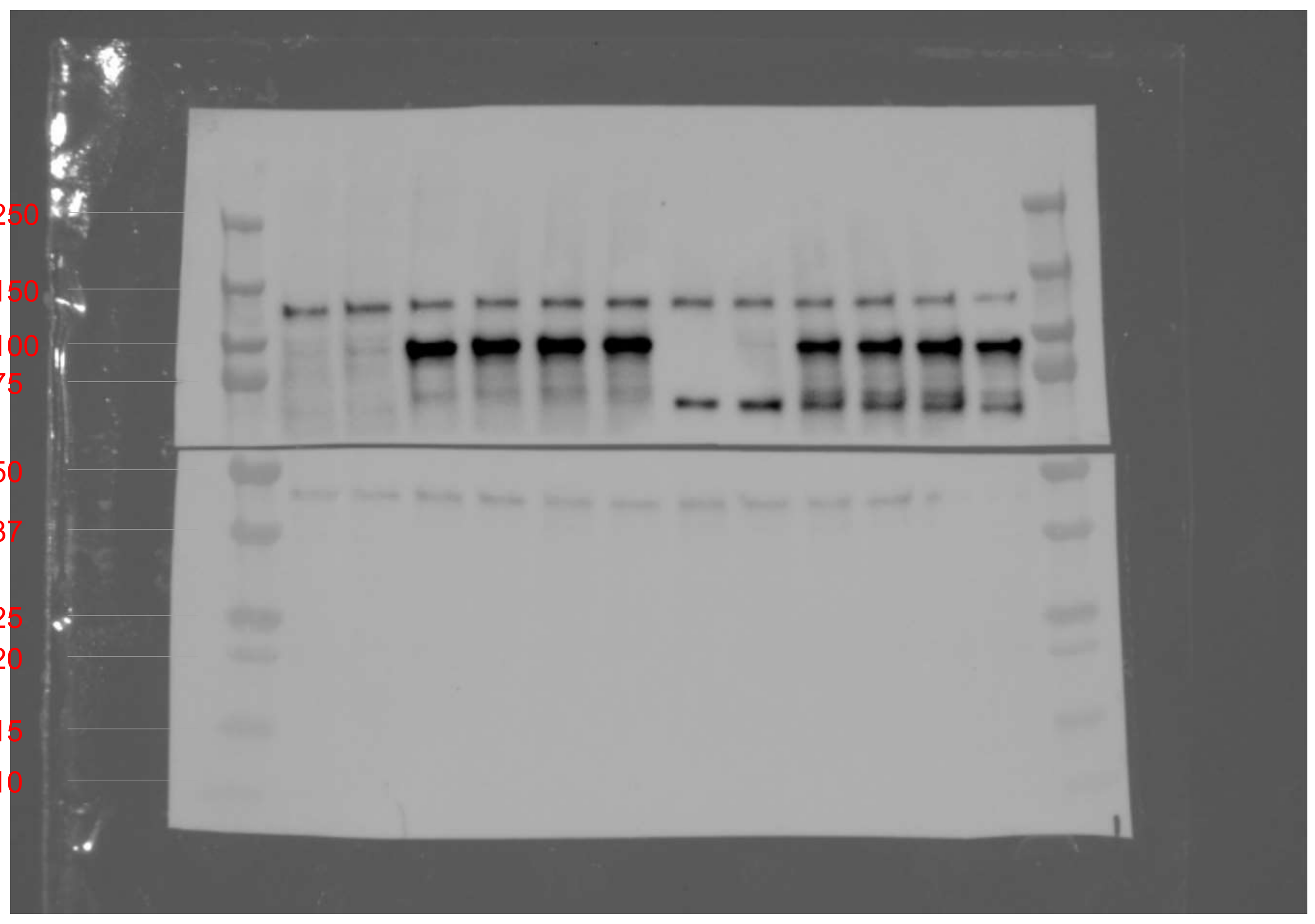

Supplementary Table 1: RNAseq-DEGs

Supplementary Table 2: Gene expression signatures used in study

Supplementary Table 3: Combo synergy genes

Supplementary Table 4: ER-HoxA9 ATAC-seq peaks-d1

Supplementary Table 5: ER-HoxA9 ATAC-seq peaks-d3

Supplementary Table 6: ER-HoxA9 ATAC-seq motif enrichments

Supplementary Table 7: THP-1 β-catenin CUT&RUN peaks

Supplementary Table 8: THP-1 IRF7 CUT&RUN peaks

Supplementary Table 9: THP-1 IRF7 and β-catenin peak intersections

Supplementary Table 10: THP-1 IRF7 and β-catenin CUT&RUN peak intersection motif enrichments

Supplementary Table 11: THP-1 IRF7 and β-catenin peak intersection Hallmark gene set database enrichments

Supplementary Table 12: Flow results GSK-LSD1 50 nM and LY2090314 100 nM in Primary samples

Supplementary Table 13: Guide RNAs used for CRISPR gene editing in this study.

Supplementary Table 14: shRNAs sequences used for knock down of genes in this study.

Supplementary Table 15: Primer sequences used in this study.

Supplementary Table 16: Primer sequences used for CUT&RUN-qPCR in this study.

Supplementary Table 13: Guide RNAs used for CRISPR gene editing in this study.

crRNA Name	Sequence
Hs.Cas9.STAT1#1	TGTGATAGGGTCATGTTCGT
Hs.Cas9.STAT1#2	CCACTAGTTCATCATTAATC

Supplementary Table 14: shRNAs sequences used for knock-down of genes in this study.

Name	Target sequence
shControl	CCTAAGGTTAAGTCGCCCTCGC
shGSK3B#1	CCCAAACTACACAGAATTTAA
shGSK3B#2	GCTGAGCTGTTACTAGGACAA
shGSK3A#1	GCTGGACCACTGCAATATTGT
shGSK3A#2	CCATAGCCCATCAAGCTCCTG

Supplementary Table 15: Primer sequences used in this study.

Primer name	Forward	Reverse
GAPDH	GCCTCAAGATCATCAGCAATGC	CCACGATACCAAAGTTGTCATGG
ITGAM(CD11b)	AACCCCTGGTTCACCTCCT	CATGACATAAGGTCAAGGCTGT
IRF7	GTGGACTGAGGGCTTGTAG	TCAACACCTGTGACTTCATGT
ISG15	GCCTCAGCTCTGACACC	CGAACTCATCTTTGCCAGTACA
MX1	GGCTGTTTACCAGACTCCGACA	CACAAAGCCTGGCAGCTCTCTA
DDX58	CCAGCATTACTAGTCAGAAGGAA	CACAGTGCAATCTTGTCATCC
ERVL	ATATCCTGCCTGGATGGGGT	GAGCTTCTTAGTCCTCCTGTGT
MER57B1	CCTCCTGAGCCAGAGTAGGT	ACCAGTCTGGCTGTTTCTGT
MER4D	CCCTAAAGAGGCAGGACACC	TCAAGCAATCGTCAACCAGA
MLT1C49	TATTGCCGTACTGTGGGCTG	TGGAACAGAGCCCTTCCTTG

Supplementary Table 16: Primer sequences used for CUT&RUN-qPCR in this study.

Region (Promoter)	Forward primer	Reverse primer
STAT1	TCTGCTCGGTCTGGGGTC	CGCTGCCTTTTCTCCTGC
STAT2	GATTAGGGTTGCAGTCCCCG	AGCTCATACTAGGGACGGGA
IFIH1	GCAGGCAGAAAGGTCAGGTA	CTTCAGGGCCAGGGTGAAAA

General flow cytometry gating for AML patient samples

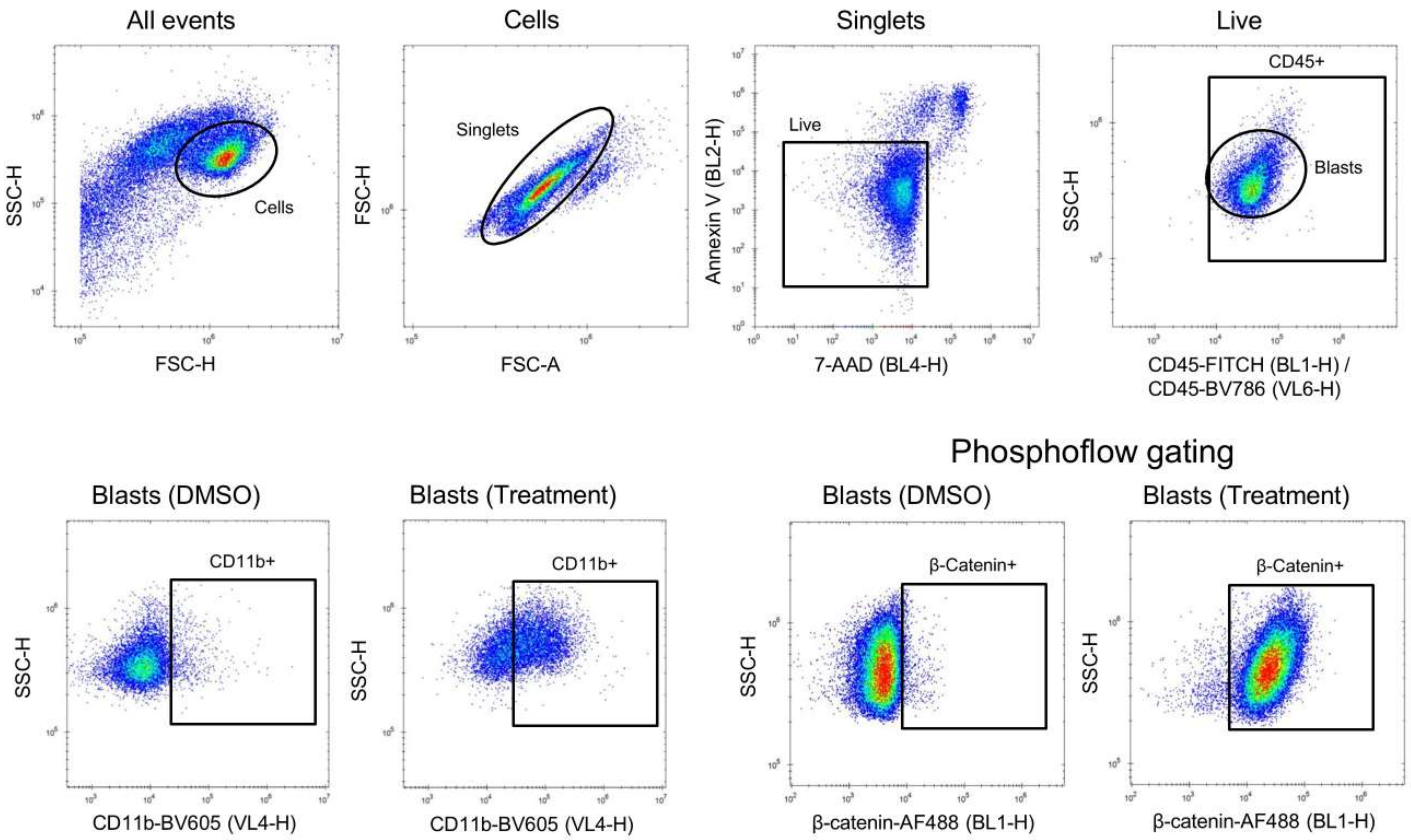

Gating strategies for LSK cell sorting

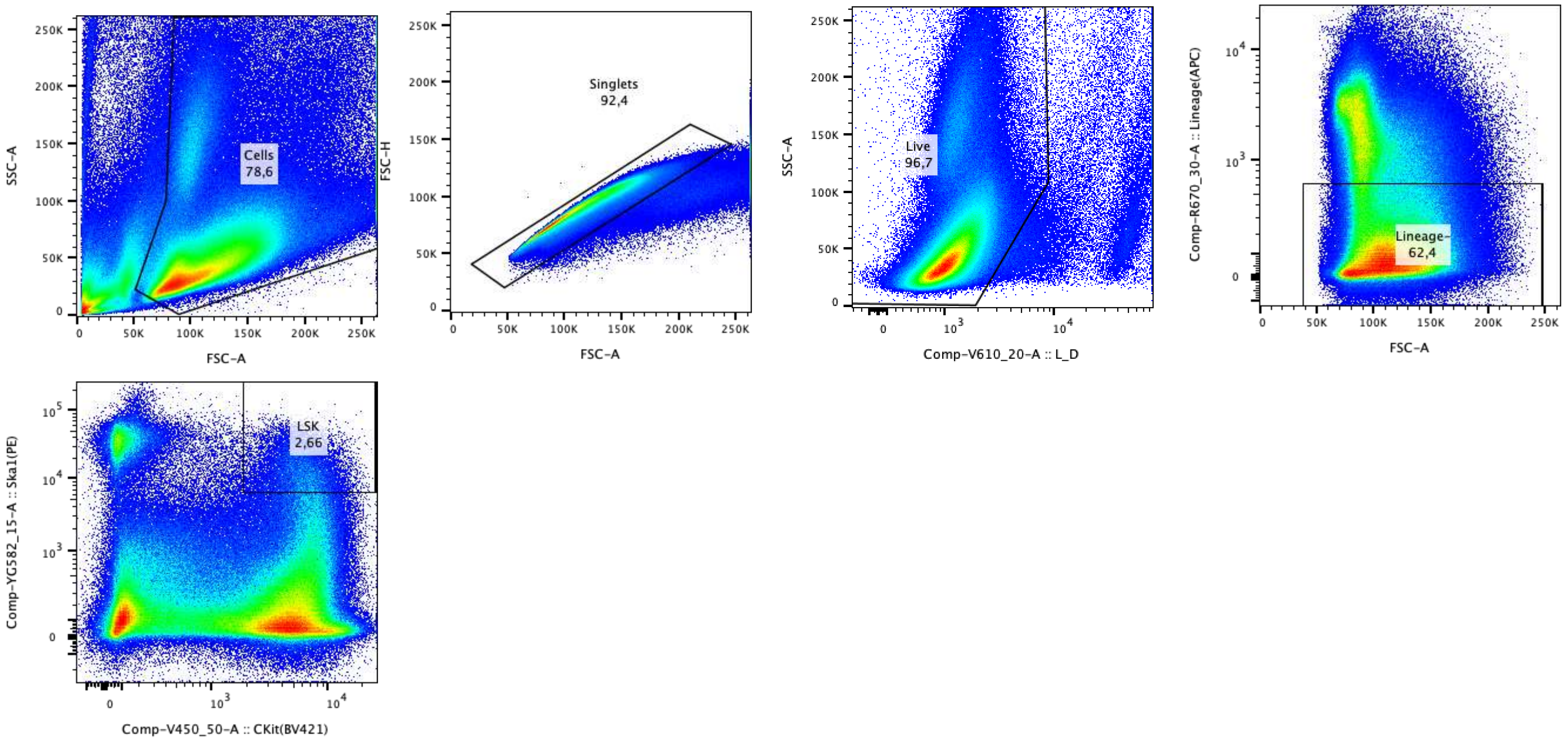

Supplement: Supplementary file 1 — This file contains the raw immunoblot images relating to Extended Data Figures 3, 5 and 6, Supplementary Tables 13–16, and flow cytometry gating/sorting strategies. [file 41586_2025_8915_MOESM1_ESM.pdf]
