# Supplementary material for: Perturbing LSD1 and WNT rewires transcription to synergistically induce AML differentiation
Source: Nature. 2025 Apr 16;642(8067):508–18. doi: 10.1038/s41586-025-08915-1 (PMC12158781; doi:10.1038/s41586-025-08915-1)
Supplement: Supplementary file 2 — Reporting Summary [file 41586_2025_8915_MOESM2_ESM.pdf]

nature portfolio

Corresponding author(s): Yang Shi, Andres Blanco, Caroline A. Heckman

Last updated by author(s): Feb 4, 2025

Reporting Summary

Nature Portfolio wishes to improve the reproducibility of the work that we publish. This form provides structure for consistency and transparency in reporting. For further information on Nature Portfolio policies, see our Editorial Policies and the Editorial Policy Checklist.

Statistics

For all statistical analyses, confirm that the following items are present in the figure legend, table legend, main text, or Methods section.

n/a	Confirmed	
☐	☒	The exact sample size (n) for each experimental group/condition, given as a discrete number and unit of measurement
☐	☒	A statement on whether measurements were taken from distinct samples or whether the same sample was measured repeatedly
☐	☒	The statistical test(s) used AND whether they are one- or two-sided<br>*Only common tests should be described solely by name; describe more complex techniques in the Methods section.*
☐	☒	A description of all covariates tested
☐	☒	A description of any assumptions or corrections, such as tests of normality and adjustment for multiple comparisons
☐	☒	A full description of the statistical parameters including central tendency (e.g. means) or other basic estimates (e.g. regression coefficient) AND variation (e.g. standard deviation) or associated estimates of uncertainty (e.g. confidence intervals)
☐	☒	For null hypothesis testing, the test statistic (e.g. F, t, r) with confidence intervals, effect sizes, degrees of freedom and P value noted<br>*Give P values as exact values whenever suitable.*
☒	☐	For Bayesian analysis, information on the choice of priors and Markov chain Monte Carlo settings
☒	☐	For hierarchical and complex designs, identification of the appropriate level for tests and full reporting of outcomes
☐	☒	Estimates of effect sizes (e.g. Cohen's d, Pearson's r), indicating how they were calculated

Our web collection on statistics for biologists contains articles on many of the points above.

Software and code

Policy information about availability of computer code

Data collection	No software was generated in this study. The Bio-Rad ChemiDoc was used for the acquisition of western blot images, and the Hamamatsu NanoZoomer S210 Slide Scanner for Cytospin slides scanning. Libraries for RNA-seq, CUT&RUN, and ATAC-seq were sequenced on the Illumina NextSeq 2000 and Illumina HiSeq 4000. Quantitative real-time PCR was conducted on the StepOnePlus instrument (Applied Biosystems). DNA/RNA concentration measurements were performed using the Thermo Fisher NanoDrop Lite (ND1000). Bioluminescent imaging was collected using the IVIS Lumina system. Ex vivo drug sensitivity of primary AML cells was analyzed using the iQue Screener Plus-VBR flow cytometer, with gating performed using ForeCyt software version 9.0 (Intellicyt). For phosphoflow analysis, cells were analyzed on the iQue PLUS flow cytometer, and data were processed using ForeCyt software version 9.0. LSK cells were sorted using a FACS Aria Fusion (BD Bioscience).
Data analysis	Graph Pad Prism 10 was used for analysis of in vivo and in vitro phenotypic assays and for most of graph production and for the statistical test.<br>Image J v1.54g was used for the analysis of colonies morphology images.<br>R v4.2 was used to analyze flow cytometry and phosphoflow results from AML patient samples.<br>For ER-Hoxa9 RNA-seq analysis, differential gene expression analysis was performed with DESeq2 (1.34.0). For THP-1 RNA-seq analysis, differential gene expression was performed with an EdgeR (3.50.3)-limma (3.36.0) workflow. Pathway analysis were performed with EnrichR and GSEA (4.3.3). For ATAC-seq, analysis was performed with FastQC (0.11.9), Cutadapt (2.1), Bowtie2 (2.4.4), samtools (1.15.1), bedtools (2.30.0), MACS2 (2.2.7.1), deeptools (3.5.1), ggplot2 (3.4.2), Homer (5.1), and the ChIPseeker (1.42.0) R (4.3.0) package. CUT&RUN samples were processed via Nextflow (21.10.6), using the nf-core CUT&RUN pipeline (v3.0.0) and further analyses were performed with Trim Galore (0.6.6), Bowtie2 (2.4.4), SEACR (1.3), deepTools (3.5.1), bedTools (2.30.0), and ChIPseeker (1.42.0). For clinical dataset survival analyses, the singscore (1.26.0) and Survival (3.8-3) R (4.3.0) packages were used.

For manuscripts utilizing custom algorithms or software that are central to the research but not yet described in published literature, software must be made available to editors and reviewers. We strongly encourage code deposition in a community repository (e.g. GitHub). See the Nature Portfolio guidelines for submitting code & software for further information.

Data

Policy information about availability of data

All manuscripts must include a data availability statement. This statement should provide the following information, where applicable:

- Accession codes, unique identifiers, or web links for publicly available datasets
- A description of any restrictions on data availability
- For clinical datasets or third party data, please ensure that the statement adheres to our policy

RNA-sequencing, ATAC-seq and CUT&RUN data have been deposited at the Gene Expression Omnibus (GEO) (https://www.ncbi.nlm.nih.gov/geo) under the accession numbers GSE249879, GSE249773 and GSE251860. The OHSU clinical AML patient dataset was downloaded from cBioPortal (https://www.cbioportal.org/study/summary?id=aml_ohsu_2022). Gene Set Enrichment Analyses (GSEAs) utilized the Molecular Signatures Database (MSigDB) (https://www.gsea-msigdb.org/gsea/msigdb). Additional pathway enrichment analyses utilized the EnrichR database (https://maayanlab.cloud/Enrichr/).

Research involving human participants, their data, or biological material

Policy information about studies with human participants or human data. See also policy information about sex, gender (identity/presentation), and sexual orientation and race, ethnicity and racism.

Reporting on sex and gender	Not reported in this study.
Reporting on race, ethnicity, or other socially relevant groupings	Not reported in this study.
Population characteristics	AML patient samples for ex vivo drug sensitivity analysis and phosphoflow experiments (n=17) were received from the Finnish Hematology Registry and Clinical Biobank. Samples were collected from patients whose disease status was either diagnosis (n=8), relapse (n=7) or refractory (n=2). Patients ages ranged from 40 to 76 years. Karyotype and mutation information was received from the hospital where the sample was collected, or it is based on whole exome sequencing data produced at the Institute for Molecular Medicine Finland. Karyotype and mutation data is provided in the supplemental Table S12. Only karyotype and mutation information were used to compare ex vivo drug sensitivity results.
Recruitment	Research material used in this project is based on an already existing cohort of samples from patients diagnosed with AML that was collected at the clinics in Finland and stored in liquid nitrogen at the Institute for Molecular Medicine Finland. Patient bone marrow or peripheral blood samples were taken in hospitals by skilled professionals after informed consent and using approved protocols in accordance with the Declaration of Helsinki. Samples were taken during routine diagnostic/treatment procedures to avoid additional procedures and inconvenience to the patients.
Ethics oversight	Samples were collected after informed consent from patients with AML using protocols approved by an Institutional Review Board at the Helsinki University Hospital (permit numbers 239/13/03/00/2010, 303/13/03/01/2011) in compliance with the Declaration of Helsinki.

Note that full information on the approval of the study protocol must also be provided in the manuscript.

Field-specific reporting

Please select the one below that is the best fit for your research. If you are not sure, read the appropriate sections before making your selection.

☒ Life sciences ☐ Behavioural & social sciences ☐ Ecological, evolutionary & environmental sciences

For a reference copy of the document with all sections, see nature.com/documents/nr-reporting-summary-flat.pdf

Life sciences study design

All studies must disclose on these points even when the disclosure is negative.

Sample size	Group sizes for the in vitro and in vivo validation experiments were selected based on prior knowledge of variation, leukemic cell engraftment amd treatment with GSK-LSD1 and LY2090314.<br>Sykes, D. B. et al. Inhibition of Dihydroorotate Dehydrogenase Overcomes Differentiation Blockade in Acute Myeloid Leukemia. Cell 167, 171-186.e15 (2016).<br>Zamek-Gliszczynski, M., J. et al. Pharmacokinetics, metabolism, and excretion of the glycogen synthase kinase-3 inhibitor LY2090314 in rats, dogs, and humans: a case study in rapid clearance by extensive metabolism with low circulating metabolite exposure. Drug Metab Dispos. 41:714-26(2013).<br>Zee, B. M. et al. Combined epigenetic and metabolic treatments overcome differentiation blockade in acute myeloid leukemia. iScience 24, 102651 (2021).<br><br>Samples from patients with AML were chosen for flow cytometry based drug sensitivity analysis based on availability of the sample vials from donors with genetic aberrations or mutations of interest.

Data exclusions	No data was excluded from the analyses.
Replication	All attempts of replication were successful and described in the legends and method section.
Randomization	For in vivo experiments mice were age-matched and randomized. No other randomization was performed, as the remaining experiments were conducted in vitro using leukemic cell lines but these cells were seeded from the same cell solution across all relevant conditions to control for seeding density and population.
Blinding	Data collection and analysis did not involve any blinding procedures. The investigators were not blinded since the collected data relied on quantitative analysis.

Reporting for specific materials, systems and methods

We require information from authors about some types of materials, experimental systems and methods used in many studies. Here, indicate whether each material, system or method listed is relevant to your study. If you are not sure if a list item applies to your research, read the appropriate section before selecting a response.

Materials & experimental systems

n/a	Involved in the study	
☐	☒	Antibodies
☐	☒	Eukaryotic cell lines
☒	☐	Palaeontology and archaeology
☐	☒	Animals and other organisms
☒	☐	Clinical data
☒	☐	Dual use research of concern
☒	☐	Plants

Methods

n/a	Involved in the study	
☒	☐	ChIP-seq
☐	☒	Flow cytometry
☒	☐	MRI-based neuroimaging

Antibodies

Antibodies used

β-Catenin (D10A8) (dilution 1:1000, Cat:8480S, Cell Signaling, Lot: 9), IRF-7 Antibody (F-1) (dilution 1:1000, Cat: sc-74471, Santa Cruz Biotechnology, Lot: I1609), α-Tubulin (DM1A) (dilution 1:1000, Cat: sc-32293, Santa Cruz Biotechnology, Lot: I1521), Vinculin (42H89L44) (dilution 1:1000, Cat: 700062 Thermo Fisher Scientific, Lot: 2616511), β-Actin (13E5) (dilution 1:1000, Cat: 4970S, Cell Signaling, Lot: 19), GSK-3α (dilution 1:1000, Cat: 9338, Cell Signaling, Lot: 6), GSK-3β (D5C5Z) (dilution 1:1000, Cat: 12456, Cell Signaling, Lot: 10), Anti-GSK3 (alpha + beta) (phospho Y216 + Y279) antibody [M132] (dilution 1:1000, Cat: ab45383, Abcam, Lot: 1089746-1), STAT1 (dilution 1:1000, Cat: 9172, Cell Signaling, Lot: 29), Phospho-STAT1 (Tyr701) Monoclonal Antibody (ST1P-11A5) (dilution 1:1000, Cat: 33-3400, Thermo Fisher scientific, Lot:YL389734), Anti-rabbit IgG, HRP-linked Antibody (dilution: 1:5000, Cat: 7074S, Cell Signaling, Lot: 33) and Anti-mouse IgG, HRP-linked Antibody (dilution: 1:5000, Cat: 7076S, Cell Signaling, Lot: 38).

Flow cytometry antibodies for ex vivo drug sensitivity testing of AML samples: CD45-FITC (1:100 dilution, HI30, BD Pharmingen, 555482/561865), CD34-APC (1:100, 8G12, BD Pharmingen, 555824), CD15-PE-Cy7 (1:80, W6D3, Biolegend, 323030), CD14-BV421 (1:50, M5E2, BD Biosciences, 565283), CD11b-BV605 (1:80, ICRF44,BD Horizon, 562721), Annexin V (1:50, BD Pharmingen, 556422), 7-AAD (1:50, BD Pharmingen, 559925).
Antibodies for phosphoflow analysis of AML samples: CD45-BV786 (1:100, HI30, BD Biosciences, 563716), CD38-BV421 (1:100, HIT2, BD Biosciences, 562444), CD34-APC-Cy7 (1:100, 581, BioLegend, 343513) and CD11b-BV605 (1:100 ICRF44, BD Horizon, 562721), Zombie Yellow (1:100, BioLegend, 423103), β-catenin-AF488 (1:25, 14/Beta-Catenin, BD Pharmingen, 562505).

Validation

For each western blot a control has been included beside knock out samples on the same blot, further manufacturer validation statements are listed as follow:
β-Catenin (Cell Signaling,8480):https://www.cellsignal.com/products/primary-antibodies/b-catenin-d10a8-xp-rabbit-mab/8480
IRF7 (Santa Cruz Biotechnology, SC-74471):https://www.scbt.com/p/irf-7-antibody-f-1
α-Tubulin (Santa Cruz Biotechnology, sc-32293):https://www.scbt.com/p/alpha-tubulin-antibody-dm1a
Vinculin (Thermo Fisher Scientific, 700062): https://www.thermofisher.com/antibody/product/Vinculin-Antibody-clone-42H89L44-Recombinant-Monoclonal/700062
STAT1 (Cell Signaling,9172):https://www.cellsignal.com/products/primary-antibodies/stat1-antibody/9172
Phospho-STAT1 (Tyr701) (Thermo Fisher scientific,33-3400): https://www.thermofisher.com/antibody/product/Phospho-STAT1-Tyr701-Antibody-clone-ST1P-11A5-Monoclonal/33-3400
β-Actin (Cell Signaling, 4970):https://www.cellsignal.com/products/primary-antibodies/b-actin-13e5-rabbit-mab/4970?srsltid=AfmBOordnCvdJ3IdlEw7zGXh7O7zefLyPIaftkRv0RPrAd-l2DMnW-x4
GSK-3α (Cell Signaling, 9338): https://www.cellsignal.com/products/primary-antibodies/gsk-3a-antibody/9338?srsltid=AfmBOoruqziJYeXHnFOFbTCaqDl_kIKpEhSz5uvswHfBuqHo7r3RcU-u
GSK-3β (D5C5Z) (Cell Signaling, 12456): https://www.cellsignal.com/products/primary-antibodies/gsk-3b-d5c5z-xp-rabbit-mab/12456?srsltid=AfmBOorVmJJ7mSEGjhR5EGo0i91VR0BrFsvoVeHopDmi_TYiFkNq8po2
GSK3 (alpha + beta) (phospho Y216 + Y279) antibody (Abcam, ab45383): https://www.abcam.com/en-us/products/primary-antibodies/gsk3-alpha-beta-phospho-y216-y279-antibody-m132-ab45383

Antibodies used for flow cytometry experiments with AML samples:
CD45-FITC: https://www.bdbiosciences.com/en-eu/products/reagents/flow-cytometry-reagents/research-reagents/single-color-

antibodies-ruo/fitc-mouse-anti-human-cd45.555482
CD34-APC: https://www.bdbiosciences.com/en-eu/products/reagents/flow-cytometry-reagents/research-reagents/single-color-antibodies-ruo/apc-mouse-anti-human-cd34.555824
CD15-PE-Cy7: https://www.biolegend.com/en-us/products/pe-cyanine7-anti-human-cd15-ssea-1-antibody-8259
CD14-BV421: https://www.bdbiosciences.com/en-eu/products/reagents/flow-cytometry-reagents/research-reagents/single-color-antibodies-ruo/bv421-mouse-anti-human-cd14.565283
CD11b-BV605: https://www.bdbiosciences.com/en-eu/products/reagents/flow-cytometry-reagents/research-reagents/single-color-antibodies-ruo/bv605-mouse-anti-human-cd11b.562721
Annexin V:https://www.bdbiosciences.com/en-eu/products/reagents/flow-cytometry-reagents/research-reagents/single-color-antibodies-ruo/pe-annexin-v.556422
7-AAD: https://www.bdbiosciences.com/en-eu/products/reagents/flow-cytometry-reagents/research-reagents/single-color-antibodies-ruo/7-aad.559925

Antibodies used for phosphoflow experiments with AML samples:
CD45-BV786: https://www.bdbiosciences.com/en-eu/products/reagents/flow-cytometry-reagents/research-reagents/single-color-antibodies-ruo/bv786-mouse-anti-human-cd45.563716
CD38-BV421: https://www.bdbiosciences.com/en-eu/products/reagents/flow-cytometry-reagents/research-reagents/single-color-antibodies-ruo/bv421-mouse-anti-human-cd38.562444
CD34-APC-Cy7: https://www.biolegend.com/en-us/products/apc-cyanine7-anti-human-cd34-antibody-6159
CD11b-BV605: https://www.bdbiosciences.com/en-eu/products/reagents/flow-cytometry-reagents/research-reagents/single-color-antibodies-ruo/bv605-mouse-anti-human-cd11b.562721
Zombie Yellow: https://www.biolegend.com/en-gb/products/zombie-yellow-fixable-viability-kit-8514
β-catenin-AF488: https://www.bdbiosciences.com/en-eu/products/reagents/flow-cytometry-reagents/research-reagents/single-color-antibodies-ruo/alexa-fluor-488-mouse-anti-catenin.562505

Eukaryotic cell lines

Policy information about cell lines and Sex and Gender in Research

Cell line source(s)	ER-HoxA9 cells were kindly provided by Dr.David T. Scadden. THP-1, MOLM-13, Kasumi-1, U937, OCI-AML2 and OCI-AML3 were obtained from Dana Farber Cancer Institute or Dr.David T. Scadden's group or purchased from DSMZ. HCT-116 cells were kindly provided by Dr. Parinaz Mehdipour, who purchased them from ATCC (ATCC-CCL-247).
Authentication	Authenticated using STR DNA profiling.
Mycoplasma contamination	The cells were confirmed to be negative for mycoplasma.
Commonly misidentified lines (See ICLAC register)	None.

Animals and other research organisms

Policy information about studies involving animals; ARRIVE guidelines recommended for reporting animal research, and Sex and Gender in Research

Laboratory animals	6 to 8 weeks old female C57BL/6J mice purchased from Jackson Laboratory. For OCI-AML3 and PDX samples, 6- to 8-week-old NSG (NOD.Cg-PrkdcSCID Il2rgtm1Wjl/SzJ) mice were purchased from Charles River. All cages were on a 12 h:12 h light: dark cycle (lights on, 07:00) in a temperature-controlled and humidity-controlled room. Room temperature was maintained at 19-23 °C, and room humidity was maintained at 45–65%.
Wild animals	No wild animal were used in this study.
Reporting on sex	All female mice were used to make sure the mouse model is truly syngeneic as the orgical ER-HoxA9/MEIS1 AML cells were derived from a female mouse. For OCI-AML3 cells both male and female mice were used.
Field-collected samples	No field-collected samples were used in this study.
Ethics oversight	Animals were maintained at Boston Children Hospital’s ARCH facility and treated according to all protocols approved by IACUC under protocol number 16-09-3230R. Invivo experiments by using OCI-AML3 and PDX samples were carried out in accordance with the terms of the UK Animals (Scientific Procedures) Act Project License (PPL) (PP4128654).

Note that full information on the approval of the study protocol must also be provided in the manuscript.

Plants

Seed stocks

Report on the source of all seed stocks or other plant material used. If applicable, state the seed stock centre and catalogue number. If plant specimens were collected from the field, describe the collection location, date and sampling procedures.

Novel plant genotypes

Describe the methods by which all novel plant genotypes were produced. This includes those generated by transgenic approaches, gene editing, chemical/radiation-based mutagenesis and hybridization. For transgenic lines, describe the transformation method, the number of independent lines analyzed and the generation upon which experiments were performed. For gene-edited lines, describe the editor used, the endogenous sequence targeted for editing, the targeting guide RNA sequence (if applicable) and how the editor was applied.

Authentication

Describe any authentication procedures for each seed stock used or novel genotype generated. Describe any experiments used to assess the effect of a mutation and, where applicable, how potential secondary effects (e.g. second site T-DNA insertions, mosiacism, off-target gene editing) were examined.

Flow Cytometry

Plots

Confirm that:

☒ The axis labels state the marker and fluorochrome used (e.g. CD4-FITC).

☒ The axis scales are clearly visible. Include numbers along axes only for bottom left plot of group (a 'group' is an analysis of identical markers).

☒ All plots are contour plots with outliers or pseudocolor plots.

☒ A numerical value for number of cells or percentage (with statistics) is provided.

Methodology

Sample preparation

AML sample preparation for flow cytometry based drug sensitivity testing and phoshpoflow experiments:
Bone marrow (BM) or peripheral blood (PB) samples were collected after informed consent from patients with AML. Mononuclear cells (MNCs) were isolated from BM or PB samples by Ficoll-Paque PREMIUM (GE Healthcare) density gradient separation and viably frozen and stored in liquid nitrogen prior to further analyses. Frozen MNCs were thawed and suspended in 12.5% conditioned medium composed of RPMI 1640 medium (Corning) supplemented with 12.5% HS-5 cell-derived conditioned medium, 10% FBS 2mM L-glutamine and penicillin-streptomycin (100U/ml) then treated with DENARASE (250U/µl, c-LEcta) to degrade DNA released from dead cells, and the cells left to recover for 4 h in 12.5% conditioned medium. The cells were plated onto pre-drugged plates at a density of 50,000 cells/well and incubated with the drugs for 5 days (37°C, 5% CO2).

Flow cytometry: After incubation, the cells were centrifuged (500 x g, 5 minutes) and resuspended in staining buffer (RPMI 1640, 10% FBS 2mM L-glutamine and penicillin-streptomycin (100U/ml)). The cells were stained with antibodies against CD45-FITC (BD Pharmingen), CD34-APC (BD Pharmingen), CD15-PE-Cy7 (Biolegend), CD14-BV421 BD Biosciences), and CD11b-BV605 (BD Horizon) for 30 minutes at room temperature in the dark. Subsequently, the cells were centrifuged (500 x g, 5 minutes) and excess antibodies were removed. The cells were resuspended and stained with PE Annexin V and 7-amino actinomycin D in Annexin V Binding Buffer (BD Pharmingen) for 15 minutes at room temperature in the dark.

Phosphoflow: After thawing and DENARASE treatment as described previously, MNCs from AML patient samples were plated onto pre-drugged Nunc™ 96-well V-bottom plate at a density of 200,000 cells/well and incubated with inhibitors for 5 days (37°C, 5% CO2). After incubation with the drugs, the cells were washed with PBS, centrifuged (1000 x g, 4 minutes) and stained with Zombie Yellow (BioLegend) viability marker for 30 minutes in the dark at room temperature. The cells were washed with staining buffer (5% FBS in DPBs) and stained with surface markers for CD45-BV786 (BD Biosciences), CD38-BV421 (BD Biosciences), CD34-APC-Cy7 (BioLegend) and CD11b-BV605 (BD Horizon) for 30 minutes at room temperature. The cells were fixed in 1.5% paraformaldehyde solution in PBS pre-warmed to 37°C, for 15 minutes at room temperature. Fixed cells were centrifuged (1000 x g for 4 minutes), washed with staining buffer, and centrifuged again with the same settings. The cells were resuspended in ice cold methanol and incubated at 4°C for 30 minutes, after which the cells were washed twice with staining buffer with centrifugation (1000g for 4 minutes). The cells were stained with β-catenin-AF488 (BD Pharmingen) for 1 hour at room temperature. After incubation, the cells were washed with staining buffer, centrifuged (1000g x 4 minutes) and resuspended for analysis.

Instrument

IntelliCyt® iQue Screener PLUS VBR, serial number 3025

Software

ForeCyt® Software v9.0.7822

Cell population abundance

Cells were only analyzed by flow cytometry, no cells were sorted.

Gating strategy

Flow cytometry for AML patient samples: Overall cell population was gated using FSC/SSC. Singlets were gated from overall cell population using FSC-A/FSC-H. Live cells were gated from singlets as Annexin V and 7-AAD negative. AML blasts were gated from CD45+dim and low SSC. CD11b+ blasts were gated from total blast population using SSC and CD11b-BV605. Unstained control was used to set the negative population. Phoshoflow analysis was done similarly, and the β-catenin positive blasts were gated with β-catenin-AF488 from total blast population.

☒ Tick this box to confirm that a figure exemplifying the gating strategy is provided in the Supplementary Information.
